# Gunacins: Novel Benzo[*g*]chromene Derivatives from the Fungus Exobasidium sp. and Their Potent Anti-Leishmania and Trypanosoma Activities

**DOI:** 10.1021/acsomega.5c01325

**Published:** 2025-05-30

**Authors:** Eva Stodůlková, Dominik Lovás, Miroslav Flieger, Alena Zíková, Jakub Zápal, Martin Štícha, Ivana Císařová, Jan Černý, Valéria Grobárová, Martina Slapničková, Tomáš Vomastek, Zuzana Klímová, Marek Kuzma, Jaroslav Semerád, Tomáš Cajthaml, Eva Cséfalvay, Winnie Cherotich Maritim, Adéla Wennrich, Marc Stadler, Tereza Ježková, Andrej Jašica, Miroslav Kolařík

**Affiliations:** † Laboratory of Fungal Genetics and Metabolism, Institute of Microbiology of the Czech Academy of Sciences, Vídeňská 1083, Prague 14220, Czechia; ‡ Laboratory of Molecular Structure Characterization, Institute of Microbiology of the Czech Academy of Sciences, Vídeňská 1083, Prague 14220, Czechia; § Department of Organic Chemistry, Faculty of Science, 37740 Charles University, Hlavova 2030/8, Prague 2 12843, Czechia; ∥ Department of Inorganic Chemistry, Faculty of Science, Charles University, Hlavova 2030/8, Prague 2 12843, Czechia; ⊥ Laboratory of Cell Immunology, Department of Cell Biology, Faculty of Science, Charles University, Viničná 1594/7, Praha 2 12843, Czechia; # Laboratory of Cell Signaling, Institute of Microbiology of the Czech Academy of Sciences, Vídeňská 1083, Prague 14220, Czechia; ¶ Institute of Parasitology, Biology Centre, Branišovská 31, České Budějovice 370 05, Czech Republic; ∇ Laboratory of Environmental Biotechnology, Institute of Microbiology of the Czech Academy of Sciences, Vídeňská 1083, Prague 14220, Czechia; ○ Institute of Microbiology of the Czech Academy of Sciences, Vídeňská 1083, Prague 14220, Czechia; ⧫ Department of Chemistry, Faculty of Sciences, 107852 Egerton University, P.O. Box 536, Egerton 20115, Kenya; †† Department of Microbial Drugs, Helmholtz Centre for Infection Research GmbH, Inhoffenstraße 7, Braunschweig 38124, Germany

## Abstract

Six new pyranonaphthoquinone derivatives, gunacin A–E (**2–7**), along with the known compounds gunacin (**1**) and the isocoumarin derivative (+) orthosporin (**8**), were isolated from the fungus Exobasidium sp. Their chemical structures were elucidated by X-ray crystallography, extensive spectroscopic analysis supported by ROESY experiments, and mass spectrometry. Two tested compounds (**1**, **5**) demonstrated high activity against Leishmania mexicana and four salivarian Trypanosoma species, with the lowest detected EC_50_ value of 0.02–0.24 μM, a value that is comparable to those of currently used drugs. In addition, compounds **1**, **3**, **5**, **6**, and **7** demonstrated antibacterial properties at micromolar concentrations, while **1**, **5**, **6**, and **7** exhibited moderate antifungal activity (MIC 33.3–66.7 μM). In cytotoxicity assays, the compounds exhibited a range of toxicity against mammalian Jurkat, RAT2, MDCK cell lines, HeLa cells, and fibroblasts, with inhibition levels varying from strong to minimal inhibition (EC_50_ = 0.03–125 μM). This study is among the first to explore Exobasidium, a genus of phytopathogenic fungi and highlights the untapped potential of smut fungi (Basidiomycota: Ustilaginomycetes). The discovery of gunacins, which exhibit potent antiprotozoal activity at submicromolar concentrations, suggests a promising avenue for the development of antiprotozoal agents.

## Graphical abstract

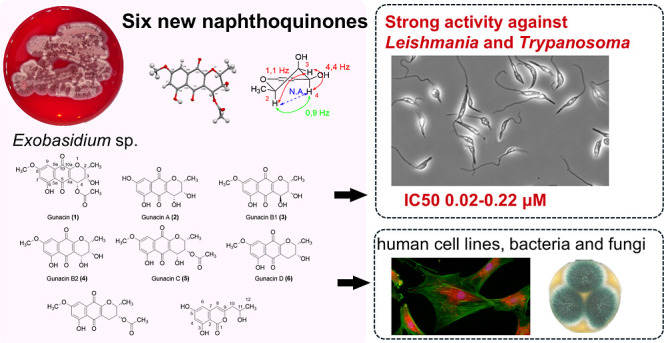

## 1 Introduction

Pyranonaphthoquinones are a diverse and widespread group of secondary metabolites found in plants,^1,2^ fungi,^3,4^ and bacteria^5,6^ with the majority possessing the 3,4-dihydro-1*H*-benzo­[*g*]­chromene-5,10-dione structural motif. Only a few pyranonaphthoquinones feature the 3,4-dihydro-2*H*-benzo­[*g*]­chromene-5,10-dione skeleton, including α-lapachone, constituents of “Lapacho tea”, used in traditional herbal medicine and believed to have anticancer effects.^7,8^ Other examples include rhinacanthin A and its derivatives from plant Rhinacanthus nasutus,^9^ α-caryopterone from Caryopteris clandonensis,^10^ and gunacin (**1**) isolated from the fungus Ustilago sp.^3^ Lapachones and rhinacanthins exhibit a wide range of biological activities, including cytotoxic,^9,11^ antibacterial,^1,12^ antifungal^13,14^ and antiprotozoal effects.^1^

Exobasidium species^15^ (Exobasidiales, Ustilaginomycotina, Basidiomycota) are worldwide distributed biotrophic plant pathogens, almost infesting plants within the Ericales order.^16^ The genus Exobasidium causes notable economic losses in tea and blueberry production.^17,18^ Despite their prevalence and importance, there have been very few studies on secondary metabolites produced by Exobasidium species. Moreover, a study describing the isolation of scopoletin and scopolin fails to distinguish whether the secondary metabolites originate from the plant host or the fungus itself.^19^ The only credible publications have described the production of auxins, 2-(1*H*-indol-3-yl) acetic acid, ethyl 2-(1*H*-indol-3-yl) acetate,^20^ and (*S*)-2-hydroxy-3-phenylpropanoic acid,^21^ by axenic Exobasidium cultures.

This study represents the first comprehensive analysis of Exobasidium secondary metabolites, their isolation, structure determination, and biological activities.

## 2 Results and Discussion

### 2.1 Structure Elucidation

In this study, we identified six new pyranonaphthoquinone derivatives, gunacin A–E (**2–7**), along with the known compounds gunacin (**1**) and the isocoumarin derivative (+) orthosporin (**8**) (Figure 1). Gunacin (**1**) (2*R*,3*S*,4*R*)-3,6-dihydroxy-8-methoxy-2-methyl-5,10-dioxo-3,4,5,10-tetrahydro-2*H*-benzo­[*g*]­chromen-4-yl acetate was obtained as orange-red needles which crystallized from the MeOH/CH_2_Cl_2_ mixture. With the molecular formula C_17_H_16_O_8_, determined by positive high-resolution electrospray ionization mass spectrometry (HRESIMS), data showed a protonated ion [M + H]^+^ at *m*/*z* 349.0920 (calcd for C_17_H_16_O_8_^+^, 349.0918).

**Figure 1:**
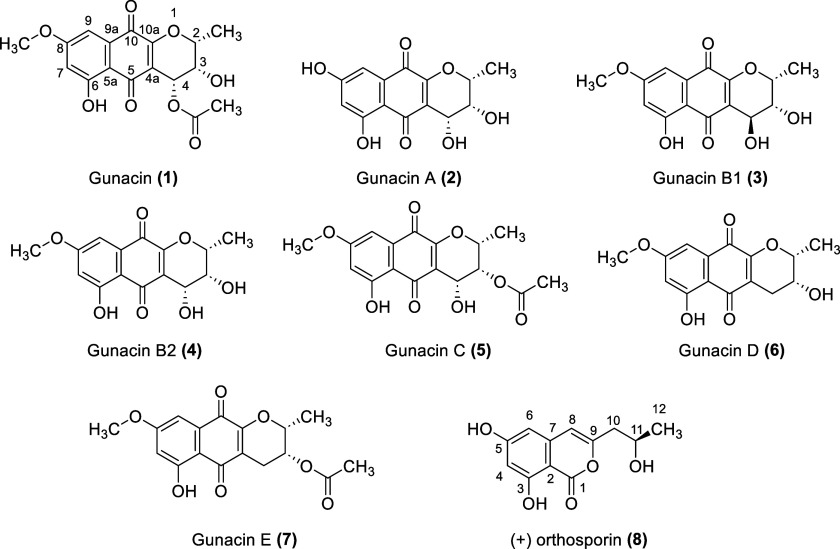
List of isolated compounds.

The ^1^H NMR spectrum of compound **1** (Table 1) displayed 16 signals of hydrogen atoms, which agrees with HRESIMS data, namely two methyls [δ_H_ 1.54 (d, *J* = 6.7 Hz), 2-Me], 2.15 (s, Ac), one methoxyl [δ_H_ 3.88, s, 8-OMe], three oxymethines [δ_H_ 4.45 (ddq, *J* = 2.1, 1.1, 6.7 Hz), H-2, 4.12 (ddd, *J* = 4.7, 2.9, 2.1 Hz), H-3, and 6.17 (dd, *J* = 4.7, 1.1 Hz), H-4], two aromatic methines [6.64 (d, *J* = 2.5 Hz), H-7 and 7.15 (d, *J* = 2.5 Hz), H-9], and two hydroxyls [2.51 (d, *J* = 2.9 Hz), 3-OH and 12.34 (s, 1H), 6-OH].

**Table 1:** ^1^H NMR Spectroscopic Data of Compounds **1–4** (700 MHz, CD_2_Cl_2_)­^c^

	**1** ^b^	**2** ^a^	**3** ^b^	**4** ^a^
position	δ_H,_ mult. (*J* in Hz)	δ_H_, mult. (*J* in Hz)	δ_H_, mult. (*J* in Hz)	δ_H_, mult. (*J* in Hz)
2	4.45 qdd (6.7, 2.1, 1.1) 1H	4.28 qdd (6.7, 1.1, 0.9) 1H	4.36 qd (6.7, 1.1) 1H	4.28 qddd (6.7, 1.3, 1.0, 0.9) 1H
3	4.12 ddd (4.7, 2.9, 2.1) 1H	4.02 dd (4.4, 1.1) 1H	3.88 dd (2.6, 1.1) 1H	4.02 m (4.4, 1.2, 1.0) 1H
4	6.17 dd (4.7, 1.1) 1H	4.98 dd (4.4, 0.9) 1H	4.71 d (2.6) 1H	4.98 m (4.4, 1.2, 0.9) 1H
7	6.64 d (2.5) 1H	6.62 d (2.5) 1H	6.63 d (2.5) 1H	6.65 d (2.5) 1H
9	7.15 d (2.5) 1H	7.09 d (2.5) 1H	7.13 d (2.5) 1H	7.17 d (2.5) 1H
2-Me	1.54 d (6.7) 3H	1.60 d (6.7) 3H	1.57 d (6.7) 3H	1.60 d (6.7) 3H
3-OH	2.51 d (2.9) 1H	n.d.	n.d.	2.94 dd (1.3, 1.2) 1H
4-OH		n.d.	n.d.	4.81 d (1.2) 1H
6-OH	12.34 s 1H	12.10 s 1H	12.33 s 1H	12.17 s 1H
8-OH		n.d.		
8-OMe	3.88 s 3H		3.88 s 3H	3.90 s 3H
Ac	2.15 s 3H			

a Measured at 20 °C.

b Measured at 5 °C.

c n.d.—signal not detected.

The ^13^C NMR and ^1^H–^13^C edited- heteronuclear single quantum correlation (HSQC) of compound **1** (Tables 1 and 2) revealed signals corresponding to 17 carbons, including two methyls (δ_C_ 15.5, 2-Me and 21.1, Ac), one methoxyl (δ_C_ 54.5, 8-Ome), five methines [including three oxygenated (δ_C_ 75.8, C-2; 66.2, C-3; 63.8, C-4) and two aromatic (δ_C_ 107.2, C-7, 108.1, C-9)], nine quaternary carbons (δ_C_ 116.4, C-4a; 108.8, C-5a; 164.02, C-6; 165.6, C-8; 132.5, C-9a; 156.7, C-10a), one acetyl (δ_C_ 170.9, 4-COO), and two ketone carbonyls (δ_C_ 178.6, C-10; 187.7, C-5).

**Table 2:** ^13^C NMR Data of Compounds **1–7** (177 MHz, CD_2_Cl_2_)

	**1** ^b^	**2** ^a^	**3** ^b^	**4** ^a^	**5** ^b^	**6** ^a^	**7** ^b^
position	δ_C_, type	δ_C_, type	δ_C_, type	δ_C_, type	δ_C_, type	δ_C_, type	δ_C_, type
2	75.8 CH	76.2 CH	73.4 CH	76.2 CH	75.2 CH	76.2 CH	74.6 CH
3	66.2 CH	67.2 CH	69.1 CH	67.2 CH	67.3 CH	64.6 CH	65.7 CH
4	63.8 CH	65.0 CH	62.9 CH	65.0 CH	63.8 CH	27.9 CH2	25.0 CH2
4a	116.4 C	118.2 C	119.5 C	118.2 C	119.0 C	118.8 C	118.3 C
5	187.7 C	191.4 C	189.5 C	191.4 C	190.7 C	189.3 C	188.9 C
5a	108.8 C	109.0 C	108.6 C	108.8 C	108.6 C	109.0 C	108.6 C
6	164.0 C	164.3 C	164.0 C	164.4 C	164.3 C	164.0 C	163.8 C
7	107.2 CH	109.3 CH	106.9 CH	107.1 CH	106.9 CH	106.8 CH	106.7 CH
8	165.6 C	163.1 C	165.7 C	166.2 C	166.0 C	165.7 C	165.5 C
9	108.1 CH	109.2 CH	108.4 CH	108.9 CH	108.9 CH	108.0 CH	108.0 CH
9a	132.5 C	133.2 C	132.7 C	132.7 C	132.5 C	133.0 C	132.7 C
10	178.6 C	178.6 C	179.1 C	178.6 C	178.5 C	178.7 C	178.6 C
10a	156.7 C	155.7 C	155.8 C	155.8 C	155.2 C	155.4 C	155.2 C
2-Me	15.5 CH_3_	16.6 CH_3_	15.9 CH_3_	16.6 CH_3_	16.5 CH_3_	16.4 CH_3_	16.7 CH_3_
8-OMe	56.5 CH_3_		56.5 CH_3_	56.6 CH_3_	56.6 CH_3_	56.5 CH_3_	56.4 CH_3_
Ac	21.1 CH_3_				20.8 CH_3_		21.1 CH_3_
3-COO					170.7 C		170.6 C
4-COO	170.9 C						

a Measured at 20 °C.

b Measured at 5 °C.

The HMBC correlations from 6-OH to C-7, C-6, and C-5a, from H-7 to C-6, C-5a, C-8, and C-9, and from H-9 to C-7, C-8, C-9a, and C-5a enabled the establishment of aromatic ring A. The HMBC correlations 8-Me to C-8 approved methoxyl at C-8.

The ^1^H–^1^H COSY correlations 2-Me/H-2, H-2/H-3, H-3/H-4, and H-3/3-OH allowed us to propose the aliphatic chain –CH_3_–CH­(O)–CH­(−OH)–CH­(O). Together with the HMBC correlations, H-2 to C-10a, H-3 to C-4a, and H-4 to C-10a helped us to build the C ring. The carboxyl carbon is coupled with H-4, and methyl of acetyl approved the acetylation at C-4.

The HMBC correlations from H-4 to C-5 and H-9 to C-10, as well as the degree of unsaturation and several long-range HMBC correlations, established ring B and finalized the structure of compound **1**. The relative configurations of C-2, C-3, and C-4 were based on NOE and coupling constants. The NOESY correlation of H-2/H-4 confirmed their orientation on the same side of the molecule (Figure 2). The given structure of gunacin (**1**) was confirmed by comparison with Werner^3^ data and by X-ray crystallography (Figure 3).

**Figure 2:**
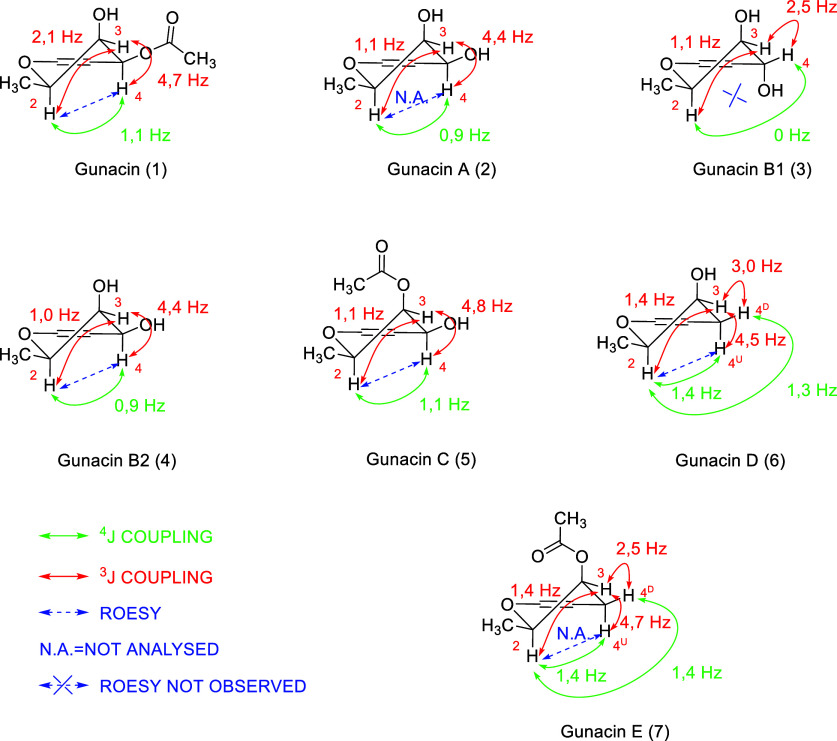
Key ^3^J and ^4^J coupling constants of 2*H*-pyran rings and ROESY correlations used to determine relative configuration.

**Figure 3:**
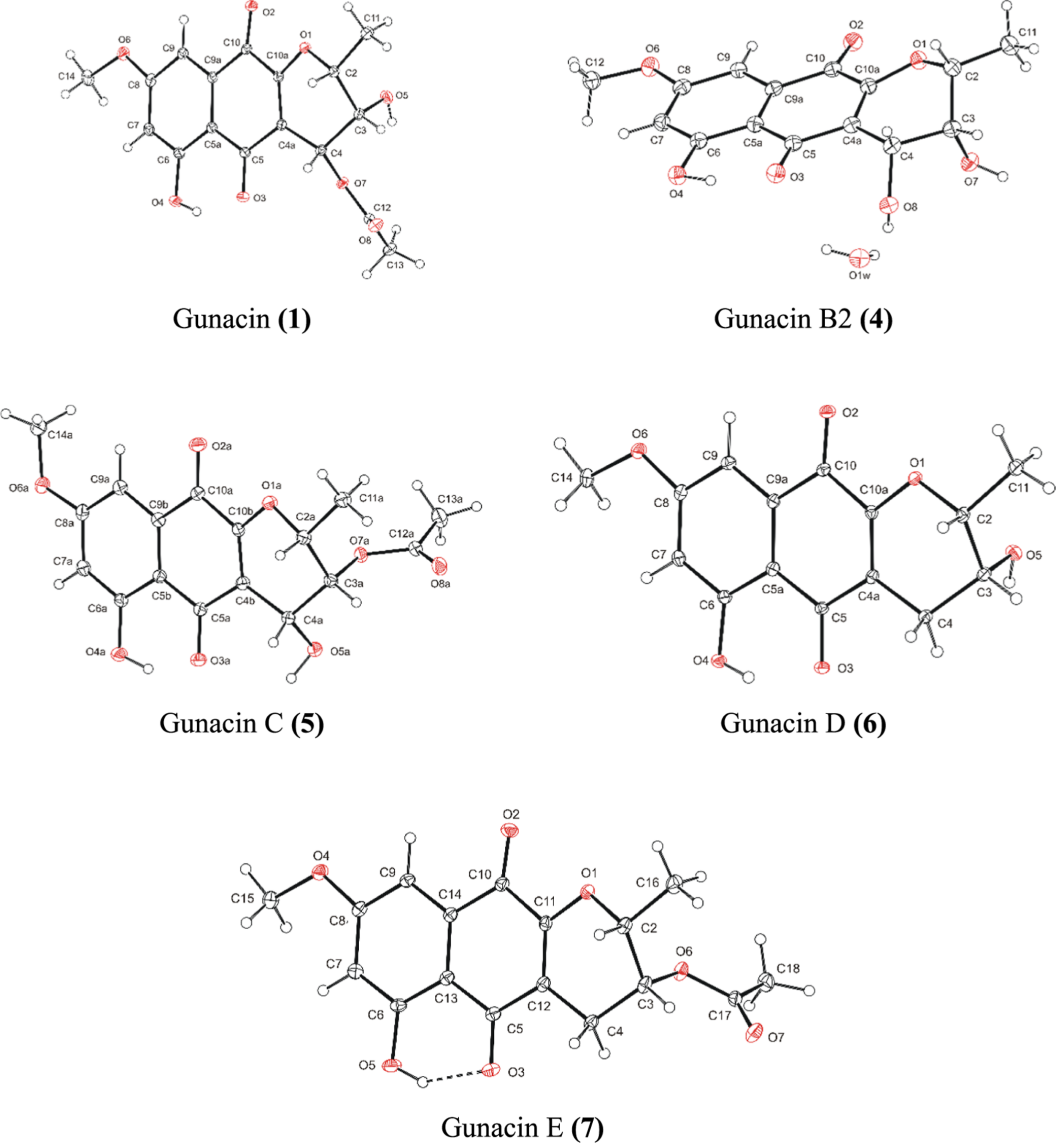
View on the molecules **1**, **4**, **5**, **6**, **7** with the atom numbering schema. Displacement ellipsoids are drawn on 30% probability level.

The structure elucidation of other molecules was based on an approach similar to that for compound **1**. The rotating frame Overhauser effect spectroscopy (ROESY) experiment and *J*-couplings determined the relative stereochemistry (Figure 2). Absolute configurations are only assumed based on comparison with data for molecule **1**.

Gunacin A (**2**), (2*R*,3*R*,4*R*)-3,4,6,8-tetrahydroxy-2-methyl-3,4-dihydro-2*H*-benzo­[*g*]­chromene-5,10-dione, was isolated as a light-yellow amorphous powder. With the molecular formula C_14_H_12_O_7_, determined by HRESIMS, data showed a protonated ion [M + H]^+^ at *m*/*z* 293.0657 (calcd for C_14_H_12_O_7_^+^, 293.0656). ^1^H and ^13^C NMR data (Tables 1 and 2) showed that **2** shares the same pyranonaphthoquinone skeleton as that of compound **1**. The absence of a proton singlet signal around δ_H_ 3.8 with 3H integral intensity in ^1^H NMR and CH_3_ signal at δ_C_ 56.5 in ^13^C NMR spectra indicates the absence of the methoxy group at position 8. Similarly, the absence of a singlet signal around δ_H_ 2.1 with 3H intensity in ^1^H NMR and CH_3_ signal at around δ_C_ 21 and carbonyl signal at δ_C_ 170 in ^13^C NMR spectra indicates the absence of an acetyl group at position 4. Given the number of oxygen and carbon atoms present in compound **2**, it was proposed that hydroxy groups replace the methoxy and acetyl groups in these positions. However, the corresponding hydrogen signals were not observed in the ^1^H NMR spectra.

Gunacin B1 (**3**) (2*R*,3*R*,4*S*)-3,4,6-trihydroxy-8-methoxy-2-methyl-3,4-dihydro-2*H*-benzo­[*g*]­chromene-5,10-dione was isolated as a yellow amorphous powder. The molecular formula C_15_H_14_O_7_ was determined by HRESIMS, and data showed a protonated ion [M + H]^+^ at *m*/*z* 307.0814 (calcd for C_15_H_14_O_7_^+^, 307.0812). ^1^H and ^13^C NMR data (Tables 1 and 2) indicate that **3** has a structure similar to compound **2**, with the key difference being the presence of a methoxy group, as evidenced by a singlet at δ_H_ 3.88 in the ^1^H NMR spectrum and a CH_3_ signal at δ_C_ 56.5 in the ^13^C NMR spectrum. However, similar to compound **2**, no hydrogen signals corresponding to the hydroxy groups at positions 3 and 4 were observed in the ^1^H NMR spectra.

Gunacin B2 (**4**) (2*R*,3*R*,4*R*)-3,4,6-trihydroxy-8-methoxy-2-methyl-3,4-dihydro-2*H*-benzo­[*g*]­chromene-5,10-dione was isolated as a yellow amorphous powder. With molecular formula C_15_H_14_O_7_, determined by HRESIMS, data showed a protonated ion [M + H]^+^ at *m*/*z* 307.0813 (calcd for C_15_H_14_O_7_^+^, 307.0812), the same as compound **3** but with slightly different fragmentation patterns. MS/MS analysis in positive mode revealed fragment ions for compound **3** at *m*/*z* 289, 271, 261, 243, 233, 219, 215, 205, 191, and 151, whereas compound **4** showed fragment ions at *m*/*z* 289, 271, 261, 235, 207, 198, and 103. In the ^1^H NMR spectrum, hydroxyl hydrogen signals for compound **4** appeared at δ_H_ 2.94 (position 3-OH) and δ_H_ 4.98 (position 4-OH). The key differences between compounds **3** and **4** include variations in the ^3^ *J* coupling constants between hydrogens at positions C-3 and C-4 (2.5 Hz in **3** vs 4.4 Hz in **4**), and the absence of a ROESY correlation between hydrogens at positions 2 and 4 in compound **4**. These observations suggest that the difference lies in the opposite configuration at position 4. The given structure of gunacin B2 (**4**) was confirmed by X-ray crystallography analysis (Figure 3).

Structures of gunacin C (**5**) (2*R*,3*R*,4*R*)-4,6-dihydroxy-8-methoxy-2-methyl-5,10-dioxo-3,4,5,10-tetrahydro-2*H*-benzo­[*g*]­chromen-3-yl acetate, gunacin D (**6**) (2*R*,3*R*)-3,6-dihydroxy-8-methoxy-2-methyl-3,4-dihydro-2*H*-benzo­[*g*]­chromene-5,10-dione, and gunacin E (**7**) (2*R*,3*R*)-6-hydroxy-8-methoxy-2-methyl-5,10-dioxo-3,4,5,10-tetrahydro-2*H*-benzo­[*g*]­chromen-3-yl acetate were determined using X-ray crystallographic analysis (Figure 2), due to their successful crystallization from MeOH/CH_2_Cl_2_ mixture. Structures were further confirmed by ^1^H and ^13^C NMR analysis (Tables 2 and 3).

**Table 3:** ^1^H NMR Data of Compounds **5–7** (700 MHz, CD_2_Cl_2_)­^c^

	**5** ^b^	**6** ^a^	**7** ^b^
position	δ_H_, mult. (J in Hz)	δ_H_, mult. (J in Hz)	δ_H_, mult. (J in Hz)
2	4.43 qdd (6.6, 1.1, 1.1) 1H	4.23 qddd (6.6, 1.4, 1.4, 1.3) 1H	4.31 qddd (6.6, 1.4, 1.4, 1.4) 1H
3	5.46 dd (4.8, 1.1) 1H	4.13 ddd (5.8, 4.5, 3.0, 1.4) 1H	5.27 ddd (4.7, 2.5, 1.4) 1H
4	5.12 ddd (4.8, 1.7, 1.1) 1H	2.77 ddd (18.8, 3.0, 1.3) 1H	2.80 ddd (19.2, 2.5, 1.4) 1H
		2.70 ddd (18.8, 4.5, 1.4) 1H	2.72 ddd (19.2, 4.7, 1.4) 1H
7	6.65 d (2.5) 1H	6.64 d (2.5) 1H	6.64 d (2.5) 1H
9	7.17 d (2.5) 1H	7.15 d (2.5) 1H	7.16 d (2.5) 1H
2-Me	1.46 d (6.6) 3H	1.51 d (6.6) 3H	1.45 d (6.6) 3H
3-OH		1.79 d (5.8) 1H	
4-OH	4.38 d (1.7) 1H		
5-OH			
6-OH	12.16 s 1H	12.43 s 1H	12.42 s 1H
11-OH			
8-OMe	3.89 s 3H	3.89 s 3H	3.88 s 3H
Ac	2.10 s 3H		2.05 s 3H

a Measured at 20 °C.

b Measured at 5 °C.

c n.d.—signal not detected.

(+) Orthosporin (**8**) 6,8-dihydroxy-3-((*S*)-2-hydroxypropyl) isochroman-1-one was isolated as a yellow, amorphous powder. With the molecular formula C_12_H_12_O_5_, determined by HRESIMS, data showed a protonated ion [M + H]^+^ at *m*/*z* 237.0754 (calcd for C_12_H_12_O_5_^+^, 237.0758). The ^1^H NMR (Table 4) spectrum revealed one methyl [δ_H_ 1.28 (d, *J* = 6.2 Hz), H-12], one methylene [δ_H_ 2.65 (dd, *J* = 14.6, 4.4 Hz), H-10d, 2.58 (dd, *J* = 14.6, 8.2 Hz), H-10u], one oxymethine [δ_H_ 4.22 (ddq, *J* = 8.2, 4.4, 6.2 Hz), H-11], three aromatic methines [δ_H_ 6.29 (s), H-8, 6.30 (d, *J* = 2.3 Hz), H-6, 6.39 (d, *J* = 2.3 Hz), H-4], and one phenolic hydroxyl [δ_H_ 11.083 (s), 11-OH].

**Table 4:** ^1^H NMR and ^13^C NMR Spectroscopic Data of Compound **8** (700 MHz for ^1^H and 177 MHz for ^13^C NMR, CD_2_Cl_2_) Measured at 20 °C

position	δ_H_, mult. (J in Hz)	δ_C_, type
1		166.5 C
2		100.6 C
3		164.1 C
4	6.39 d (2.3) 1H	102.2 CH
5		163.7 C
6	6.30 d (2.3) 1H	102.7 CH
7		140.1 C
8	6.29 s 1H	106.1 CH
9		155.6 C
10	2.65 dd (14.6, 4.4) 1H	43.3 CH_2_
	2.58 dd (14.6, 8,2) 1H	
11	4.22 qdd (6.2, 8.2, 4.4) 1H	65.9 CH
12	1.28 d (6.2) 3H	23.5 CH_3_
3-OH	11.08 s 1H	
5-OH	n.d.	
11-OH	n.d.	

The ^13^C NMR (Table 4) and ^1^H–^13^C edited-HSQC spectra showed the presence of 12 carbon signals with the following multiplicity: one methyl (δ_C_ 23.5, C-12), one methylene (δ_C_ 43.3, C-10), four methines [including three aromatic/olephinic (δ_C_ 102.2, C-4; 102.7, C-6 and 106.1, C-8)], one oxymethine (δ_C_ 65.9, C-11), five nonprotonated carbons (δ_C_ 100.5, C-2; 155.6, C-9; 140.1, C-7; 163.7, C-5; 164.1, C-3), and one lactone carbonyl (δ_C_ 166.5, C-1).

The HMBC correlations from 3-OH to C-2, C-3, and C-4, from H-4 to C-2, C-5, and C-6, and from H-6 to C-2 and C-5 completed the A ring description, with the exception of carbon C-7, which was only identified later by its weak HMBC correlation to neighboring H-8.

The ^1^H–^1^H COSY correlations of H-12/H-11 and H-11/H-10 built up the side chain. The HMBC correlations from H-11 to C-9 and H-10 to C-8 indicate the connection between the side chain and tri-substituted double bond. The HMBC correlations from H-8 to C-6 and C-2 connect it with the aromatic A ring.

Two hydrogens not observed in ^1^H NMR were proposed to be part of the two hydroxyl groups. The data indicate that the structure is consistent with the known isocoumarin compound orthosporin, as confirmed by comparison with published NMR data.^22^ Its absolute configuration at C-11 was determined as S through comparison with previously published optical rotation data.^22^

Additionally, exposure of **1** or **5** to an acidic 5% solution of MeOH/H_2_O led to the formation of **5** or **1**, respectively. The proposed mechanism of this reaction is intramolecular transacylation. A similar mechanism has been described for salvinorin E and salvinorin D, terpenes isolated from Salvia divinorum.^23^ Furthermore, prolonged exposure resulted in the formation of **4**. Formation of **3**, whose structure is the same as the structure of **4**, except for the configuration at position 4, was not observed. Therefore, the proposed mechanism for this reaction involves SN1 hydrolysis of the ester in an acidic MeOH solution.

### 2.2 Biological Activity

The antimicrobial activity of compounds **1**, **3**, **5**, **6**, and **7** was evaluated against model microorganisms, including Escherichia coli, Kocuria rhizophila, Cryptococcus neoformans, and Candida albicans. All tested compounds demonstrated better inhibitory activity against bacteria compared with the positive control, chloramphenicol. The activity against yeasts was approximately on par with the positive control, cycloheximide, except for **3**, which showed no inhibitory activity even at the highest tested concentration of 66.7 μM (Table 5).

**Table 5:** Minimal Inhibition Concentration (MIC) of Compounds Gunacin (**1**), Gunacin A (**2**), Gunacin B1 (**3**), Gunacin B2 (**4**), Gunacin C (**5**), Gunacin D (**6**), and Gunacin E (**7**) against Model Pathogenic Microorganisms E. coli, K. rhizophila, C. neoformans, and C. albicans ^a^

	MIC (μM)			
compounds	*E. coli*	*K. rhizophila*	*C. neoformans*	*C. albicans*
**1**	1.0	1.0	33.3	33.3
**2**	•	•	•	•
**3**	2.1	8.3	n.d. (>66.7)	n.d. (>66.7)
**4**	•	•	•	•
**5**	1.0	2.1	33.3	66.7
**6**	2.1	8.3	66.7	66.7
**7**	4.2	4.2	33.3	33.3
chloramphenicol	10.3	10.3	•	•
cycloheximide	•	•	35.5	35.5

a n.d. = activity not detected. • = activity not measured.

The cytotoxic activity of compounds **1**, **3**, **4**, **5**, **6**, and **7** was evaluated against the Jurkat T-lymphocyte cell line, RAT2 fibroblast cell line, Madin–Darby canine kidney (MDCK) epithelial cell line, and rainbow trout RTL-W1 cell line, revealing notable differences among the compounds. Compounds **1** and **5** demonstrated similar EC_50_ values, with approximately 1.5 μM for Jurkat cells, while their effects on RAT2 and MDCK cells were observed at submicromolar concentrations. The effects on the RTL-W1 cell line varied depending on the fluorescent indicators used: Alamar Blue (AB), 5-carboxyfluorescein diacetate acetoxymethyl ester (CFDA), and Neutral Red (NR). Compound **1** exhibited EC_50_ values in the submicromolar range, while compound **5** showed EC_50_ values in the low micromolar range. Compound **3** exhibited slightly lower toxicity against Jurkat cells, while compound **7** showed the lowest toxicity across all tested cell lines (Table 6). Compounds **3**, **4**, and **6** exhibited relatively low toxicity against the RAT2 cell line, with EC_50_ values of 5.1, 5.1, and 6.7 μM, respectively, and against the MDCK cell line, with EC_50_ values of 4.7, 6.0, and 7.2 μM, respectively.

**Table 6:** EC_50_ Values (Mean ± SEM of Three Measurements) of Compounds **1**, **3**, **4**, **5**, **6**, and **7** for Cytotoxicity against the Jurkat T-Lymphocyte Cell Line, Measured by Flow Cytometry (FACS LSRII), RAT2 Fibroblast and MDCK Kidney Cell Lines, Determined by Crystal Violet Assay and OD^590^ Measurement, and Toxicity against RTL-W1 Cell Line Were Evaluated Using Three Fluorescent Indicators: AB, 5-Carboxyfluorescein Diacetate Acetoxymethyl Ester (CFDA), and NR, with Fluorescence Measurements Performed at Excitation/Emission Wavelengths of 535/630 nm^a^

	EC_50_ ± SEM (μM)					
compounds	Jurkat	RAT2	MDCK	RTL-W1 (AB)	RTL-W1 (CFDA)	RTL-W1 (NR)
**1**	1.7 ± 0.1	0.06 ± 0.0	0.13 ± 0. 0	0.5 ± 0.0	0.8 ± 0.1	0.3 ± 0.0
**2**	•	•	•	•	•	•
**3**	16.5 ± 1.3	5.1 ± 0.7	4.7 ± 0.6	•	•	•
**4**	•	5.1 ± 0.1	6.0 ± 1.0	•	•	•
**5**	1.6 ± 0.1	0.03 ± 0.0	0.1 ± 1.0	2.5 ± 0.3	2.1 ± 0.1	0.9 ± 0.1
**6**	•	6.7 ± 0.8	7.2 ± 0.5	n.d.	n.d.	n.d.
**7**	93.2 ± 5.9	6.0 ± 0.30	5.6 ± 1.1	n.d.	n.d.	n.d.

a • = activity not measured. n.d. = activity not detected at highest tested concentration 150 μM.

In terms of the morphological effects, HeLa cells and human fibroblasts were examined. The efficacy of **5** on primary fibroblasts is evidenced by the complete disappearance of the MitoTracker Red CMXRos mitochondrial signal at a concentration of 5 μM, which is indicative of mitochondrial dysfunction, particularly with regard to the mitochondrial membrane potential. At higher concentrations, the cells lose their adherence to the surface. At lower concentrations, the actin cytoskeleton remains unaffected, showing the compound’s selectivity for mitochondria. In contrast, HeLa cells are more resistant, with effects on mitochondrial physiology only observed at the highest tested concentration of 125 μM, while the actin skeleton remains unchanged. Compound **1** has similar activity as **5**, HeLa cells are significantly more resistant, with notable alterations in cellular morphology only at 125 μM. Effects on fibroblasts were observed at 5 μM, including a loss of polymerized actin and mitochondrial signal. Compound **7** did not show significant toxicity even at 125 μM on Hela cells, except for a slight decrease in the green signal for actin. The concentration 25 μM did not show any notable effect on fibroblast cell line but concentration 125 μM caused complete loss of live adherent cells. Compound **3** was more potent against HeLa cells, showing a weaker morphological effect at 25 μM but caused complete loss of live adherent cells at 125 μM, with only a weak effect on mitochondrial signal in fibroblast cells at 125 μM (Figure 4).

**Figure 4:**
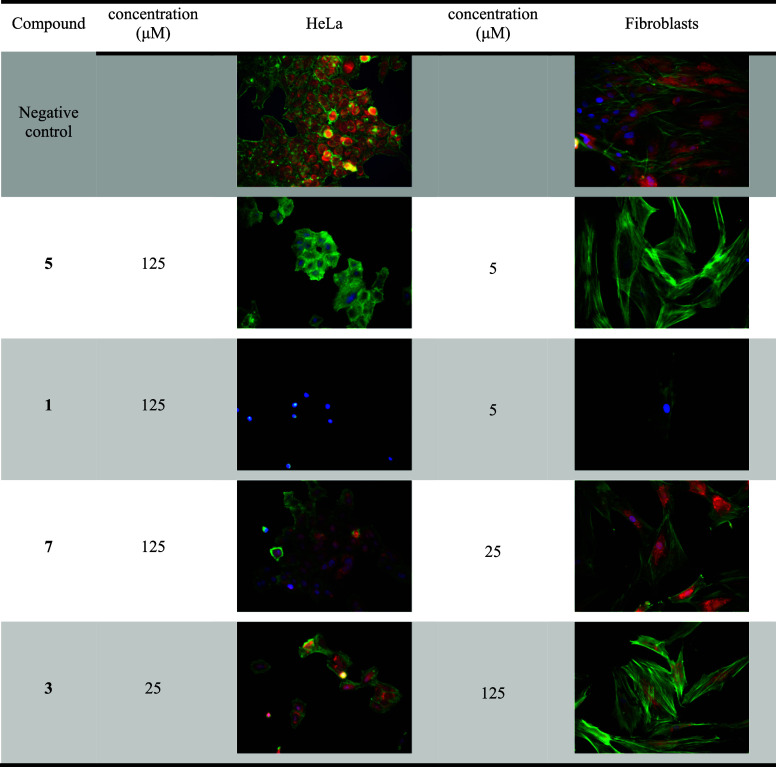
Visualization of the effects of compound **1**, **3**, **5**, **7** on the morphology of HeLa adenocarcinoma cell line (left) and primary human skin fibroblasts (right). DMSO was used as a solvent and as a control. Mitochondria are visualized using MitoTracker Red CMXRos (red), the actin cytoskeleton with Phalloidin (green), and nuclei with DAPI (blue).

The ability of compounds **1**, **3**, **5**, **6**, and **7** to inhibit the proliferation of the bloodstream mammalian form of several salivarian Trypanosoma species, as well as Leishmania mexicana promastigotes and amastigotes, was evaluated. The compound cytotoxicity was tested on parasites grown axenically. Compounds **1** and **5** demonstrated the strongest antiprotozoal activity, effective at submicromolar concentrations, markedly lower than those required for reference compounds amphotericin B and hygromycin across all tested strains. The promising lead compounds should be evaluated in future studies for their effects on parasites in infection-relevant settings. For example, in the case of Leishmania species, demonstrating that the compounds inhibit the parasites’ ability to infect and replicate within macrophages as amastigotes would further support their potential. In contrast, compounds **3**, **6**, and **7** exhibited activity weaker or comparable to that of the positive controls (Table 7).

**Table 7:** Activity of Gunacin (**1**), Gunacin B1 (**3**) Gunacin C (**5**), Gunacin D (**6**), Gunacin E (**7**) against Leishmania and Trypanosoma Species

	EC_50_ ± SEM (μM)						
compound	**1**	**3**	**5**	**6**	**7**	amphotericin B	hygromycin
T. brucei	0.06	4.3	0.06	5.1	0.9	•	1.26
	±0.01	±2.3	±0.01	±1.8	±0.5		±0.37
T. brucei evansi	0.03	4.2	0.04	2.4	0.7	•	0.76
	±0.01	±1.7	±0.01	±0.4	±0.2		±0.09
T. brucei gambiense	0.04	4.1	0.03	8.6	5.7	•	0.64
	±0.004	±0.8	±0.005	±3.0	±0.3		±0.22
T. congolense	0.02	5.8	0.02	9.2	2.4	•	0.075
	±0.01	±0.5	±0.005	±1.04	±1.1		±0.01
L. mexicana promastigote	0.22	8.6	0.24	25.2	3.6	0.1	•
	±0.01	±0.5	±0.02	±3.1	±0.5		
L. mexicana amastigote	0.01	5.6	0.01	5.5	2.5	0.3	•
	±0.005	±0.6	±0.006	±0.6	±0.1	±0.03	

The only previously published work on compound **1** is by Werner,^3^ who described the isolation of **1** from the fungus Ustilago sp. The strain of Ustilago sp., isolated from soil, was identified solely based on morphology. This morphology is similar to that of the strain of Exobasidium sp. we studied, and we do not rule out the possibility that it could be the same fungus. In addition, **1**, **4**, and **5** appear to be enantiomeric forms of the compounds isolated from the ascomycete fungus Scytalidium flavobrunneum and presented in the patent of Elson et al.^24^ However, that study lacks sufficient data, including missing optical rotation values, to unambiguously confirm their identity.

The 3,4-dihydro-2*H*-benzo­[g]­chromene-5,10-dione structural motif is recognized for its antibacterial, antifungal, and cytotoxic properties.^25^ This study presents a new class of compounds with this motif, exhibiting antibacterial effects at micromolar concentrations consistent with the findings of Werner.^3^ The compounds related or identical to **1**, **4**, and **5** presented by Elson et al.^24^ were found to inhibit monocyte chemoattractant protein 1 (MCP-1). The proposed applications include the treatment of atherosclerosis and other diseases involving excessive monocyte infiltration and macrophage-mediated tissue damage, which highlight additional potential uses for gunacins. Notably, these compounds display significant antiprotozoal activity, with compound gunacin (**1**) showing an EC_50_ of 0.01 μM against L. mexicana AMA, surpassing the efficacy of current antiprotozoal drugs like amphotericin B, which has an EC_50_ of 0.03 μM against the same strain. While previous research by Al Nasr^26^ reported antiprotozoal activities of synthetic pyranonaphthoquinone derivatives against Leishmania major and Trypanosoma brucei at micromolar levels. However, **1** as the most promising compound also exhibits cytotoxicity against the Jurkat (EC_50_ = 1.7 μM), RAT2 (EC_50_ = 0.1 μM), and MDCK (EC_50_ = 0.1 μM) cell lines, warranting further evaluation of its selectivity and therapeutic potential.

Our study is also one of the few focusing on metabolites from smut fungi (specifically the order Exobasidiales), demonstrating that these overlooked fungi are potent producers of secondary metabolites. Little is known about the ecological role of the compounds they produce. The species we studied is not known to develop a pathogenic phase, which is otherwise typical for all members of the genus Exobasidium. Thus, it remains unclear whether these metabolites are involved in plant interactions. Instead, they might play a role in microbial competition within their environment.

## 3 Experimental Procedures

### 3.1 General Experimental Procedures

Optical rotations were determined using a Rudolph Research Analytical Autopol III automatic polarimeter in MeOH or acetone. NMR spectra were obtained using a Bruker Avance III 700 MHz spectrometer (700 MHz for ^1^H, 176 MHz for ^13^C) and a Bruker Avance III 600 MHz spectrometer (600 MHz for ^1^H, 151 MHz for ^13^C). All samples were measured in CD_2_Cl_2_. The spectra were referenced by the residual signal of the solvent (CD_2_Cl_2_: δ_H_ 5.323 ppm, δ_C_ 53.87 ppm). HRESIMS analysis and tandem mass spectrometry were performed on a Bruker qTOF Compact instrument with Agilent 1290 Infinity II high-performance liquid chromatography (HPLC) instrument equipped with Kinetex biphenyl columns (100 × 2.1; 100 Å). Semipreparative HPLC was carried out on a Waters instrument equipped with a 2487 UV detector and Gemini C18 column (5 μm, 110 Å, 250 × 10.00 mm). Column chromatography (CC) was performed on Sephadex LH-20 (150 g, GE Healthcare Bio-Science, Sweden), STRATA C18 column (20 g, 55 μm, 70 Å Phenomenex); silica gel (0.035–0.070 mm, 70 Å, Lachner) fractions were analyzed by high-performance liquid chromatography (HPLC) with UV–vis detection using an Alliance HPLC 2988 dual photodiode array (PDA) detector equipped with a Gemini C18 analytical column (5 μm, 110 Å, 250 × 4.6 mm). The melting point was determined using CNYST micromelting point measuring instrument and are uncorrected.

### 3.2 Fungal Material

The production strain was isolated from the surface of healthy leaves of Tilia cordata (Marianka, Slovakia, 48°14′49″, N 17°04′02″ E, T. Ježová 6. 11. 2022). The medium used for isolation was a combination of modified DRBC (Dichloran Rose-Bengal Chloramphenicol) medium and Christensen’s urea agar (glucose 10 g, peptone 5 g, KH_2_PO_4_ 2 g, MgSO_4_ 0.5 g, phenol red 0.012 g, maltose extract 2 g, urea 20 g, chloramphenicol 0.1 g, 1 M HCl 1.5 mL, dichloran 0.002 g, agar 15 g, and 948.5 L of distilled water). The isolated strain was classified as Exobasidium sp. based on the ITS rDNA sequence (Genbank Accession no. PV253747) and morphology using the procedures described in Kolařík et al.^27^ Based on the BlastN similarity search in NCBI Genbank, the ITS barcode has the best hits to described species 96.4% (EU692771, Exobasidium canadense) and 96.5% (KY424480, Exobasidium japonicum). The strain is deposited in the Culture Collection of Fungi (Department of Botany, Faculty of Sciences, Charles University) under the number CCF 7021.

### 3.3 Fermentation, Extraction, and Isolation

Exobasidium sp. was cultivated on modified YM6.3 liquid medium (maltose extract 10 g, glucose 4 g, yeast extract 4 g, NaCl 50 g, and 1 L of distilled water) on a rotary shaker (200 rpm) for 30 days at 24 °C to a total volume of 6 L. The suspension was separated into fermentation broth and biomass by centrifugation (4000*g*, 15 °C, 20 min) and subsequent filtration. Both the broth and biomass were then extracted stepwise with toluene, dichloromethane, ethyl acetate, and ethyl acetate acidified with acetic acid to pH 3. The individual organic phases were separated, dried over sodium sulfate, and concentrated using a rotary vacuum evaporator to obtain crude extracts.

Individual extracts were diluted in CH_2_Cl_2_ and subjected to column chromatography on Sephadex LH-20 (150 g, GE Healthcare Bio-Science, Sweden) and equilibrated in the same solvent. Elution was performed first with CH_2_Cl_2_, followed by a stepwise gradient of CH_2_Cl_2_/MeOH at ratios of 100:0.5, 100:1, 100:2, 100:6.25, and 100:12.5 (v/v). Collected fractions were combined based on HPLC analysis. Pure compounds were isolated from the toluene extract/gunacin (17.6 mg) (100:0.5), gunacin D (3.2 mg) (100:1), and gunacin B1 (9.7 mg) (100:6.25). The combined fractions from Sephadex LH-20 chromatography were diluted in MeOH, subjected to a STRATA C18 column (20 g, 55 μm, 70 Å Phenomenex) activated with MeOH, and eluted using a gradient of MeOH–H_2_O (0%, 10%, 20%, ... 100%). Fractions were again collected and combined based on HPLC analysis, resulting in the isolation of pure compounds gunancin A (2.1 mg) (50%) and gunacin C (21.3 mg) (70%). In the final step, the combined fractions were subjected to isolation using semipreparative HPLC, elution was performed isocratically with mobile phases A: 5% MeOH +0.1% (v/v) TFA and B: MeOH + 0.1% (v/v) TFA at a flow rate of 2 mL/min, which led to the isolation of gunacin B2 (45% A) (8.3 mg), gunacin E (35% A) (6.7 mg), and orthosporin (45% A) (1.8 mg).

### 3.4 Spectroscopic Data

Gunacin (**1**) (2*R*,3*S*,4*R*)-3,6-dihydroxy-8-methoxy-2-methyl-5,10-dioxo-3,4,5,10-tetrahydro-2*H*-benzo­[g]­chromen-4-yl acetate, orange-red needles (CH_2_Cl_2_), mp 198 °C, [α]_*D*_^25^ + 222 (c 0.13; MeOH), ^1^H and ^13^C NMR, see Tables 1 and 2, MS/MS (37.4 eV, positive mode) 289, 271, 261, 243, 228, 215, 187, 151, HRESIMS *m*/*z* 349.0920 [M + H]^+^ (calcd for C_17_H_16_O_8_^+^ 349.092).

Gunacin A (**2**) (2*R*,3*R*,4*R*)-3,4,6,8-tetrahydroxy-2-methyl-3,4-dihydro-2*H*-benzo­[*g*]­chromene-5,10-dione, light yellow amorphous powder, mp n.d., [α]_*D*_^25^ + 100 (c 0.13; MeOH), ^1^H and ^13^C NMR, see Tables 1 and 2, MS/MS (37.4 eV, positive mode) 275; 257; 247; 229; 219; 201; 191; 173; 137, HRESIMS *m*/*z* 293.066 [M + H]^+^ (calcd for C_14_H_12_O_7_^+^ 293.066).

Gunacin B1 (**3**) (2*R*,3*R*,4*S*)-3,4,6-trihydroxy-8-methoxy-2-methyl-3,4-dihydro-2*H*-benzo­[*g*]­chromene-5,10-dione, yellow amorphous powder, mp n.d., [α]_*D*_^25^ = +11 (c 0.23; MeOH), ^1^H and ^13^C NMR, see Tables 1 and 2, MS/MS (37.4 eV, positive mode) 289, 271, 261, 243, 233, 219, 215, 205, 191, 151, HRESIMS *m*/*z* 307.0814 [M + H]^+^ (calcd for C_15_H_14_O_7_^+^ 307.081).

Gunacin B2 (**4**) (2*R*,3*R*,4*R*)-3,4,6-trihydroxy-8-methoxy-2-methyl-3,4-dihydro-2*H*-benzo­[*g*]­chromene-5,10-dione, yellow amorphous powder, mp n.d., [α]_*D*_^25^ = +144 (c 0.04; MeOH), ^1^H and ^13^C NMR, see Tables 1 and 2, MS/MS (37.4 eV, positive mode) 289, 271, 261, 235, 207, 198, 103, HRESIMS *m*/*z* 307.0813 [M + H]^+^ (calcd for C_15_H_14_O_7_^+^ 307.081).

Gunacin C (**5**) (2*R*,3*R*,4*R*)-4,6-dihydroxy-8-methoxy-2-methyl-5,10-dioxo-3,4,5,10-tetrahydro-2*H*-benzo­[*g*]­chromen-3-yl acetate, orange needles (CH_2_Cl_2_), mp 162 °C [α]_*D*_^25^ = +238 (c 0.25; MeOH), ^1^H and ^13^C NMR, see Tables 1 and 2, MS/MS (37.4 eV, positive mode) 289, 271, 261, 235, 207, 198, 103, HRESIMS *m*/*z* 349.0920 [M + H]^+^ (calcd for C_17_H_16_O_8_^+^ 349.092).

Gunacin D (**6**) (2*R*,3*R*)-3,6-dihydroxy-8-methoxy-2-methyl-3,4-dihydro-2*H*-benzo­[*g*]­chromene-5,10-dione, orange needles (CH_2_Cl_2_), mp n.d., [α]_*D*_^25^ = −113 (c 0.06; Acetone), ^1^H and ^13^C NMR, see Tables 1 and 2, MS/MS (37.4 eV, positive mode) 235, 233, 217, 205, 177, 151, 83, HRESIMS *m*/*z* 291.0864 [M + H]^+^ (calcd for C_15_H_14_O_6_^+^ 291.086).

Gunacin E (**7**) (2*R*,3*R*)-6-hydroxy-8-methoxy-2-methyl-5,10-dioxo-3,4,5,10-tetrahydro-2*H*-benzo­[*g*]­chromen-3-yl acetate, orange needles (CH_2_Cl_2_), mp 216 °C, [α]_*D*_^25^ = +12 (c 0.05; MeOH), ^1^H and ^13^C NMR, see Tables 1 and 2, MS/MS (37.4 eV, positive mode) 291; 273; 255; 245; 233; 227; 217; 203; 189; 175; 151, HRESIMS *m*/*z* 333.0967 [M + H]^+^ (calcd for C_17_H_16_O_7_^+^ 333.097).

Orthosporin (**8**) 6,8-dihydroxy-3-((*S*)-2-hydroxypropyl)­isochroman-1-one, colorless [α]_*D*_^25^ + 46.1 (c 0.13; MeOH), ^1^H and ^13^C NMR, see Table 4, MS/MS (37.4 eV, positive mode) 219, 201, 191, 177, 163, 149, 135, 121, 107, HRESIMS *m*/*z* 237.0754 [M + H]^+^ (calcd for C_12_H_12_O_5_^+^ 237.076).

### 3.5 X-ray Crystal Structure Analysis

X-ray diffraction data were collected on Bruker D8 VENTURE Kappa Duo PHOTONIII by IμS microfocus sealed tube CuKα (λ = 1.54178 Å). The structures were solved by direct methods (XT^28^) and refined by full matrix least-squares based on F2 (SHELXL2019).^29^ The hydrogen atoms in the hydroxy groups were identified with difference electron density maps and refined under presumption of rigid-body movements. Hydrogens on carbon atoms were placed in calculated positions. The displacement parameters of all hydrogen atoms were derived from the temperature movements of their corresponding pivot atoms. The determination of the absolute structures of **6** and **7** was based on the anomalous dispersion of oxygen atoms.^30^ Whereas for structures **1**, **2**, and **5** with the high standard deviation of the chirality parameter, the assignment of the absolute structure was based on the chirality of the C2 carbon, which is expected to be preserved in the whole series of gunacins.

The crystallographic data have been deposited into the Cambridge Crystallographic Data Centre with CCDC numbers 2404168, 2404169, 2404170, 2404171, and 2292108; **1**, **4**, **5**, **6**, and **7**, respectively. It is available free of charge from the Cambridge Crystallographic Data Centre, 12 Union Road, Cambridge CB2 1 EZ, UK; at www.ccdc.cam.ac.uk/structures/

### 3.6 Crystallographic Data

Gunacin (**1**): C_17_H_16_O_8_, *M*_w_ = 348.30; monoclinic, *P*12_1_1, *a* = 12.4852(4) Å, *b* = 4.6313(1) Å, *c* = 14.0155(4) Å, β = 110.872 (1)° *V* = 757.23(4) Å^3^, *Z* = 2, *D*_*x*_ = 1.528 g/m^3^, temperature of sample 120(2) K, orange-red prism of dimensions 0.89 × 0.20 × 0.14 mm, multi-scan absorption correction (μ = 1.05 mm^–1^) *T*_min_ = 0.57, *T*_max_ = 0.86; a total of 24024 measured reflections (θ_max_ = 77.2°), from which 3151 were unique (*R*_int_ = 0.038) and 3102 observed according to the *I* > 2σ­(*I*) criterion. The refinement converged (Δ/σ_max_ < 0.001) to *R* = 0.034 for observed reflections and w*R*(*F* ^2^) = 0.094, GOF = 1.04 for 229 parameters and all 3151 reflections. The final difference map displayed no peaks of chemical significance (Δρ_max_ = 0.26, and Δρ_min_ = −0.22 e/Å^3^). Absolute structure parameter: 0.18 (16).

Gunacin B2 (**4**): C_15_H_14_O_7_·H_2_O, *M*_w_ = 324.28; monoclinic, *P*12_1_1, *a* = 10.9217 (4) Å, *b* = 4.6601 (2) Å, *c* = 13.4899 (4) Å, β = 93.901 (3)°, *V* = 684.99 (4) Å^3^, *Z* = 2, *D*_*x*_ = 1.572 g/m^3^, temperature of sample 120(2) K, yellow needle of dimensions 0.43 × 0.02 × 0.02 mm, multiscan absorption correction (μ = 1.11 mm^–1^) *T*_min_ = 0.78, *T*_max_ = 0.98; a total of 22519 measured reflections (θ_max_ = 74.8°), from which 2613 were unique (*R*_int_ = 0.095) and 2140 observed according to the *I* > 2σ­(*I*) criterion. The refinement converged (Δ/σ_max_ < 0.001) to *R* = 0.064 for observed reflections and w*R*(*F* ^2^) = 0.148, GOF = 1.08 for 210 parameters and all 2613 reflections. The final difference map displayed no peaks of chemical significance (Δρ_max_ = 0.25 and Δρ_min_ = −0.29 e/Å^3^). Absolute structure parameter: −0.3 (5).

Gunacin C (**5**): 2­(C_17_H_16_O_8_)·CH_4_O, *M*_w_ = 728.64, orthorhombic, *P*2_1_2_1_2_1_, *a* = 7.2067(2) Å, *b* = 14.5880(4) Å, *c* = 30.9566(9) Å, *V* = 3254.51(16) Å^3^, *Z* = 4, *D*_*x*_ = 1.487 g/m^3^, temperature of sample 120(2) K, orange needle of dimensions 0.27 × 0.03 × 0.02 mm, multiscan absorption correction (μ = 1.02 mm^–1^) *T*_min_ = 0.70, *T*_max_ = 0.98; a total of 24815 measured reflections (θ_max_ = 77.3°), from which 6774 were unique (*R*_int_ = 0.090) and 5666 observed according to the *I* > 2σ­(*I*) criterion. The refinement converged (Δ/σ_max_ < 0.001) to *R* = 0.048 for observed reflections and w*R*(*F* ^2^) = 0.115, GOF = 1.01 for 476 parameters and all 6774 reflections. The final difference map displayed no peaks of chemical significance (Δρ_max_ = 0.23, Δρ_min_ = −0.37 e/Å^3^). Absolute structure parameter: −0.14 (16).

Gunacin D (**6**): C_15_H_14_O_6_, *M*_w_ = 290.26, triclinic, *P*1, *a* = 4.7911(2) Å, *b* = 5.6136(2) Å, *c* = 12.0015(5) Å, α = 97.079 (2)°, β = 98.421 (2)°, γ = 98.784 (2)°, *V* = 312.04(2) Å^3^, *Z* = 1, *D*_*x*_ = 1.545 g/m^3^, temperature of sample 120(2) K, orange plate of dimensions 0.24 × 0.09 × 0.04 mm, multiscan absorption correction (μ = 1.02 mm^–1^) *T*_min_ = 0.84, *T*_max_ = 0.96; a total of 6373 measured reflections (θ_max_ = 72.0°), from which 2306 were unique (*R*_int_ = 0.025) and 2278 observed according to the *I* > 2σ­(*I*) criterion. The refinement converged (Δ/σ_max_ < 0.001) to *R* = 0.028 for observed reflections and w*R*(*F* ^2^) = 0.082, GOF = 1.08 for 193 parameters and all 2306 reflections. The final difference map displayed no peaks of chemical significance (Δρ_max_ = 0.25, Δρ_min_ = −0.17 e/Å^3^). Absolute structure parameter: 0.03 (9).

Gunacin E (**7**): C_17_H_16_O_7_, *M*_w_ = 332.30, orthorhombic, *P*2_1_2_1_2_1_, *a* = 7.4150(3) Å, *b* = 9.2420(3) Å, *c* = 21.7346(8) Å, *V* = 1489.46(9) Å^3^, *Z* = 4, *D*_*x*_ = 1.482 g/m^3^, temperature of sample 120(2) K, orange bar of dimensions 0.55 × 0.07 × 0.04 mm, multiscan absorption correction (μ = 0.99 mm^–1^) *T*_min_ = 0.78, *T*_max_ = 0.96; a total of 12829 measured reflections (θ_max_ = 79.0°), from which 3149 were unique (*R*_int_ = 0.033) and 3105 observed according to the *I* > 2σ­(*I*) criterion. The refinement converged (Δ/σ_max_ < 0.001) to R = 0.031 for observed reflections and w*R*(*F* ^2^) = 0.084, GOF = 1.04 for 220 parameters and all 3149 reflections. The final difference map displayed no peaks of chemical significance (Δρ_max_ = 0.21, Δρ_min_ = −0.24 e/Å^3^). Absolute structure parameter: 0.02 (6).

### 3.7 Cytotoxic Assays

To evaluate the cytotoxicity of the isolated compounds, the immortalized T-lymphocyte cell line Jurkat was cultured in 96-well polypropylene U-bottom plates at a density of approximately 2 × 10^5^ cells per well in RPMI1640 medium, with a total volume of 300 μL per well. Cells cultured in RPMI1640 medium alone and in RPMI1640 medium with DMSO were used as negative controls. After 24 h of incubation with the isolated compounds, dissolved in 10 mM DMSO, the cells were washed with PBS solution containing 0.02% gelatin and 0.01% sodium azide. Following this, the cells were stained with the fluorescent dye Hoechst 33258 and analyzed by flow cytometry using a FACS LSRII instrument (BD Biosciences, San Jose, USA) and FlowJo 10 software (Tree Star, Ashland, USA). Initial assays were conducted at concentrations of 5, 25, 50, 100, and 125 μM, with subsequent measurements at concentrations adjusted based on the results of the initial assays, each performed in triplicate. EC_50_ values were determined using Quest Graph software and the dose–response four parameters regression model.^31^ The EC_50_ values were determined from three independent experiments.

MDCK and RAT cells were cultured in DMEM with 10% FBS at 37 °C in a 5% CO_2_ atmosphere as described previously.^32,33^ To assess the toxic effects of the compounds on proliferating cells, cells were seeded in 24-well plates for 24 h to reach ∼15% confluence, and cells were treated in triplicate with increasing concentrations of the isolated compounds for an additional 24 h. Cells were washed with 1× PBS, fixed in staining solution (0.5% crystal violet, 20% methanol) for 10 min, and gently washed five times in 1× PBS. The fixed cells were then dissolved in lysis solution (0.1 M sodium citrate, 25% ethanol, pH 4.2) for 30 min, transferred to a 96-well plate in H_2_O, and the OD^590^ was determined. EC_50_ values were determined using GraphPad Prism software and the dose–response fit equation. The EC_50_ values were determined from two independent experiments. Additionally, the impact of the isolated substances on the morphology of the HeLa adenocarcinoma cell line and primary human skin fibroblasts was examined by using fluorescence microscopy. Cells were cultured in DME medium supplemented with 10% FCS (Gibco, Invitrogen, Carlsbad, USA) on glass coverslips in 24-well plates until reaching approximately 50% confluence. The cells were treated with varying concentrations of isolated compounds dissolved in DMSO. Wells containing DMEM with DMSO served as the negative control. After 24 h cultivation, the cells were incubated with MitoTracker Red CMXRos (10 min) to label mitochondria, then fixed with 3.7% paraformaldehyde in PBS (20 min, room temperature), permeabilized with 0.1% Triton X-100 in PBS, and blocked with 1% BSA in PBS. The cells were then stained with Phalloidin-Alexa Fluor488 conjugate. All fluorescence reagents were purchased from Molecular Probes, Invitrogen, Carlsbad, CA, USA. Nuclei were stained with Fluoroshield DAPI (Sigma-Aldrich), and cells were examined using an IX71 microscope equipped with a DP70 camera and a 40× objective. Assays were performed at concentrations of 5, 25, 50, 100, and 125 μM.

### 3.8 Viability Assay

Cell viability assay^34^ was determined on cell line RTL-W1 (obtained from Eawag, Switzerland; liver tissue) of rainbow trout (Oncorhynchus mykiss) according to the modified procedure of Dayeh (2005).^35^ A combination of three fluorescent indicators, alamarBlue (AB), 5-carboxyfluorescein diacetate acetoxymethyl ester (CFDA-AM), and NR was used to assess different mechanisms of toxic action, evaluate the general cellular response and increase the sensitivity of the cytotoxicity tests.^36^ AB tests cellular metabolic activity, CFDA-AM assay assesses cell membrane integrity, and NR displays the integrity of lysosomal membranes. After 24 h of incubation in microculturing plates, the cells were exposed to concentration series of isolated compounds in 0.5% DMSO for another 24 h. After the exposition, the solutions were removed from individual wells followed by the washing step and the addition of 100 μL of a dye solution containing 0.625% AB and 0.4 μM CFDA-AM in L15ex. The cells were incubated at room temperature for 30 min, and the fluorescence was measured at excitation/emission wavelengths of 535/590 nm for the AB assay and 485/535 nm for the CFDA-AM assay. Afterward, the dye solution was removed, the cells were rinsed, and 100 μL of the NR solution was added (0.03 mg/mL in L15ex). After 60 min of incubation, the cells were rinsed twice, and NR was extracted from the cells using 150 μL of a solution consisting of 1% (v/v) glacial acetic acid in 50% (v/v) ethanol. The content of each well was repeatedly homogenized using a pipet, and the fluorescence was measured at excitation/emission wavelengths of 535/630 nm.

### 3.9 Antimicrobial Assays

Minimum inhibitory concentration (MIC) testing of the extracts was performed using 96-well polypropylene plates against four different microorganisms: the Gram-positive bacterium K. rhizophila ATCC 9341, the Gram-negative bacterium E. coli ATCC 3988, and the yeasts C. albicans CCM 8215 and C. neoformans CCF 1081.

A working inoculum of each microorganism was prepared as follows: for E. coli and K. rhizophila, the inoculum was grown in LB medium (LB Broth, 25 g/L, incubated at 36 °C); for C. albicans and C. neoformans, YM6.3 medium (maltose extract 10 g, glucose 4 g, yeast extract 4 g per 1 L distilled water, incubated at 24 °C) with a cell concentration of approximately 5 × 10^5^ cells/mL for all microorganisms was used.

Chloramphenicol served as a positive control for bacteria, while cycloheximide was used as a positive control for fungi. The gradient of the test substances was established by serial binary dilution across the wells. The MIC was determined by identifying the lowest concentration at which no live microorganisms were present. Cultivation was performed in the dark for 24 h.

### 3.10 In Vitro Activity against T. b. gambiense, T. b. brucei, T. brucei evansi, and T. congolense bloodstreams

The bloodstream forms of T. b. gambiense LiTat 1.3, T. b. brucei 427, and T. b. evansi AnTat 3/3 were grown in HMI-11 medium pH 7.3 supplemented with 36 mM sodium bicarbonate, 100 U/mL penicillin/streptomycin, and 10% fetal bovine serum at 37 °C and 5% CO_2_.^37^ The bloodstream form of Trypanosoma congolense IL3000 (generous gift from Liam Morrison) was cultured in TcBSF-1 medium pH 7.3 supplemented with 26 mM sodium bicarbonate, 25 mM HEPES, 5.5 mM glucose, 1 mM sodium pyruvate, 0.04 mM adenosine, 0.1 mM hypoxanthine, 0.02 mM thymidine, 0.02 mM bathocuproine, 2 mM l-glutamine, 0.2 mM 2-mercaptoethanol, 100 U/ml penicillin/streptomycin, and 20% goat serum New Zealand origin (Gibco) at 34 °C, 5% CO_2_.^38^ The assay for anti-trypanosomal activity was performed using the resazurin sodium salt dye (AB Assay) according to the published protocol^39^ in a 96-well plate format. Parasites at a number of 5 × 10^3^ per well (T. b. brucei and T. b. gambiense) or 1 × 10^4^ per well (T. b. evansi) were incubated with different drug concentrations (2-fold serial dilutions) in a volume of 200 μL of the medium. Medium with 1% DMSO (the highest concentration of the solvent used in the assay) was used to confirm no effect of the solvent on the cell growth. The plates were incubated for 48 h at the appropriate temperature. Then, 20 μL of resazurin sodium salt solution (0.125 mg/mL in 1× PBS, pH 7. 4) was added to each well, and the cells were incubated for another 24 h under the same conditions. The fluorescence signal was quantified using a Tecan Infinite M200 plate reader at excitation and emission wavelengths of 560 and 590 nm, respectively. The EC_50_ values were calculated using GraphPad Prism 9.5.1 by nonlinear regression with a variable slope. Each EC_50_ value is the mean ± standard error of the mean of three separate experiments performed in duplicate.

### 3.11 In Vitro Activity against L. mexicana Promastigote and Amastigote Life Cycle Stages

Promastigote stages of L. mexicana (MNYC/BZ/1962/M379), a generous gift from Vyacheslav Yurchenko, were cultured in M199 medium pH 7.4 supplemented with 2 μg/mL biopterin, 2 μg/mL hemin, 25 mM HEPES, 100 U/mL penicillin/streptomycin, and 10% heat-inactivated FBS (BioSera) pH 7.4 at 25 °C.^40^ The amastigotes were differentiated in vitro from late log/stationary promastigote cultures and cultivated in Schneider’s Drosophila Medium (SIM) at pH 5.5, supplemented with 1.5 μg/mL hemin, 100 U/mL penicillin/streptomycin, and 20% heat-inactivated FBS (BioSera) at pH 5.5, at 32 °C, and 5% CO_2_.^41^ Following a three-day period, the promastigotes underwent a transformation into amastigotes, which were then maintained in SIM.

To estimate the EC_50_, the AB assay, as previously described, was employed. The results are expressed as EC_50_, representing the dose of the compound required to inhibit cellular growth by 50%. Each EC_50_ value is the mean ± the standard error of the mean derived from at least three separate experiments, each performed in duplicate.

## Supplementary Material



## Notes

HPLC chromatograms, MS/MS, HRESIMS and NMR spectra; and detailed results from bioassays (PDF)
