# Supplementary material for: Gunacins: Novel Benzo[g]chromene Derivatives from the Fungus Exobasidium sp. and Their Potent Anti-Leishmania and Trypanosoma Activities
Source: ACS Omega. 2025 May 30;10(22):23222–34. doi: 10.1021/acsomega.5c01325 (PMC12163684; doi:10.1021/acsomega.5c01325)
Supplement: Supplementary file 1 [file ao5c01325_si_001.pdf]

Gunacins: Novel Benzo[g]chromene Derivatives from the fungus *Exobasidium* sp. and Their Potent Anti-Leishmania and Trypanosoma Activities

Eva Stodůlková[†], Dominik Lovás [†], Miroslav Flieger, Alena Zíková, Jakub Zápal, Martin Šťícha, Ivana Císařová, Jan Černý, Valéria Grobárová, Martina Slapničková, Tomáš Vomastek, Zuzana Klímová, Marek Kuzma, Jaroslav Semerád, Tomáš Cajthaml, Eva Cséfalvay, Winnie Cherotich Maritim, Adéla Wennrich, Marc Stadler, Tereza Ježková, Andrej Jašica, Miroslav Kolařík*

[†] – shared first author position

Eva Stodůlková – Laboratory of Fungal Genetics and Metabolism, Institute of Microbiology of the Czech Academy of Sciences, Vídeňská 1083, 14220, Prague, Czechia

Dominik Lovás – Laboratory of Fungal Genetics and Metabolism, Institute of Microbiology of the Czech Academy of Sciences, Vídeňská 1083, 14220, Prague, Czechia

Miroslav Flieger – Laboratory of Fungal Genetics and Metabolism, Institute of Microbiology of the Czech Academy of Sciences, Vídeňská 1083, 14220, Prague, Czechia

Jakub Zápal – Laboratory of Structural Biology and Cell Signalling, Institute of Microbiology of the Czech Academy of Sciences, Vídeňská 1083, 14220, Prague, Czechia

Martin Šťícha – Department of Organic Chemistry, Faculty of Science, Charles University, Hlavova 2030/8, 12843, Prague 2, Czechia

Ivana Císařová – Department of Inorganic Chemistry, Faculty of Science, Charles University, Hlavova 2030/8, 12843, Prague 2, Czechia

Jan Černý – Laboratory of Cell Immunology, Department of Cell Biology, Faculty of Science, Charles, Viničná 1594/7, Praha 2, Czechia

Valéria Grobárová – Laboratory of Cell Immunology, Department of Cell Biology, Faculty of Science, Charles, Viničná 1594/7, Praha 2, Czechia

Martina Slapničková – Institute of Parasitology, Biology Centre, Branišovská 31, České Budějovice, Czech Republic

Alena Zíková – Institute of Parasitology, Biology Centre, Branišovská 31, České Budějovice, Czech Republic

Tomáš Vomastek – Laboratory of Cell Signalling, Institute of Microbiology of the Czech Academy of Sciences, Vídeňská 1083, 14220, Prague, Czechia

Marek Kuzma – Laboratory of Structural Biology and Cell Signalling, Institute of Microbiology of the Czech Academy of Sciences, Vídeňská 1083, 14220, Prague, Czechia

Jaroslav Semerád – Laboratory of Environmental Biotechnology, Institute of Microbiology of the Czech Academy of Sciences, Vídeňská 1083, 14220, Prague, Czechia

Zuzana Klímová – Laboratory of Cell Signalling, Institute of Microbiology of the Czech Academy of Sciences, Vídeňská 1083, 14220, Prague, Czechia

Tomáš Cajthaml – Institute of Microbiology of the Czech Academy of Sciences, Vídeňská 1083, 14220, Prague, Czechia

Eva Caéfalvay - Institute of Microbiology of the Czech Academy of Sciences, Vídeňská 1083, 14220, Prague, Czechia

Winnie Cherotich Maritim – Department of Chemistry, Faculty of Sciences, Egerton University, P.O. Box 536, 20115, Egerton, Kenya

Adéla Čmoková – Laboratory of Fungal Genetics and Metabolism, Institute of Microbiology of the Czech Academy of Sciences, Vídeňská 1083, 14220, Prague, Czechia

Marc Stadler – Department of Microbial Drugs, Helmholtz Centre for Infection Research GmbH, Inhoffenstraße 7, 38124, Braunschweig, Germany

Tereza Ježková – Laboratory of Fungal Genetics and Metabolism, Institute of Microbiology of the Czech Academy of Sciences, Vídeňská 1083, 14220, Prague, Czechia

Andrej Jašica – Laboratory of Fungal Genetics and Metabolism, Institute of Microbiology of the Czech Academy of Sciences, Vídeňská 1083, 14220, Prague, Czechia

Corresponding Author

Miroslav Kolařík – Laboratory of Fungal Genetics and Metabolism, Institute of Microbiology of the Czech Academy of Sciences, Vídeňská 1083, 14220, Prague, Czechia

List of supplementary material

HPLC 7

Figure S1 Chromatograph of crude extract 7

Mass spectroscopy data of compounds 1-8 8

Figure S2 Negative MS/MS spectrum of **1** 8

Figure S3 Positive MS/MS spectrum of **1** 9

Figure S4 Negative HRESIMS spectrum of **1** 10

Figure S5 Positive HRESIMS spectrum of 1 11

Figure S6 Negative MS/MS spectrum of 2 12

Figure S7 Positive MS/MS spectrum of **2** 13

Figure S8 Negative HRESIMS spectrum of **2** 14

Figure S9 Positive HRESIMS spectrum of **2** 15

Figure S10 Negative MS/MS spectrum of **3** 16

Figure S11 Positive MS/MS spectrum of **3** 17

Figure S12 Negative HRESIMS spectrum of **3** 18

Figure S13 Positive HRESIMS spectrum of **3** 19

Figure S14 Negative MS/MS spectrum of **4** 20

Figure S15 Positive MS/MS spectrum of **4** 21

Figure S16 Negative HRESIMS spectrum of **4** 22

Figure S17 Positive HRESIMS spectrum of **4** 23

Figure S18 Positive MS/MS spectrum of **5** 24

Figure S19 Positive HRESIMS spectrum of **5** 25

Figure S20 Positive MS/MS spectrum of **6** 26

Figure S21 Positive HRESIMS spectrum of **6** 27

Figure S22 Positive MS/MS spectrum of **7** 28

Figure S23 Positive HRESIMS spectrum of **7** 29

Figure S24 Negative MS/MS spectrum of **8** 30

Figure S25 Positive MS/MS spectrum of **8** 31

Figure S26 Negative HRESIMS spectrum of **8** 32

Figure S27 Positive HRESIMS spectrum of **8** 33

NMR data of compounds 1-8 34

Figure S28 ^{1}H NMR spectrum of **1** (CD_2Cl_2, 278.2 K, 600.23 MHz) 34

Figure S29 ^{13}C NMR spectrum of **1** (CD_2Cl_2, 278.2 K, 150.93 MHz) 35

Figure S30 ^{1}H NMR spectrum of **2** (CD_2Cl_2, 293.2 K, 600.23 MHz) 36
Figure S31 ^{13}C NMR spectrum of **2** (CD_2Cl_2, 293.2 K, 150.93 MHz) 37
Figure S32 ^{1}H NMR spectrum of **3** (CD_2Cl_2, 278.2 K, 700.13 MHz) 38
Figure S33 ^{13}C NMR spectrum of **3** (CD_2Cl_2, 278.2 K, 176.05 MHz) 39
Figure S34 ^{1}H NMR spectrum of **4** (CD_2Cl_2, 293.2 K, 700.13 MHz) 40
Figure S35 ^{13}C NMR spectrum of **4** (CD_2Cl_2, 293.2 K, 176.05 MHz) 41
Figure S36 ^{1}H NMR spectrum of **5** (CD_2Cl_2, 278.2 K, 700.13 MHz) 42
Figure S37 ^{13}C NMR spectrum of **5** (CD_2Cl_2, 278.2 K, 176.05 MHz) 43
Figure S38 ^{1}H NMR spectrum of **6** (CD_2Cl_2, 293.2 K, 600.23 MHz) 44
Figure S39 ^{13}C NMR spectrum of **6** (CD_2Cl_2, 293.2 K, 150.93 MHz) 45
Figure S40 ^{1}H NMR spectrum of **7** (CD_2Cl_2, 278.2 K, 700.13 MHz) 46
Figure S41 ^{13}C NMR spectrum of **7** (CD_2Cl_2, 278.2 K, 176.05 MHz) 47
Figure S42 ^{1}H NMR spectrum of **8** (CD_2Cl_2, 293.2 K, 700.13 MHz) 48
Figure S43 ^{13}C NMR spectrum of **8** (CD_2Cl_2, 293.2 K, 176.05 MHz) 49
Biological activity data of compounds 1, 5, 7 50
Figure S44 Cytotoxic activity of **5** against MDCK cell line 50
Figure S45 Cytotoxic activity of **7** against MDCK cell line 50
Figure S46 Cytotoxic activity of **5** against RAT2 cell line 51
Figure S47 Cytotoxic activity of **7** against RAT2 cell line 51
Figure S48 Antitrypanosomal activity of **1** against *Trypanosoma brucei brucei* 52
Figure S49 Antitrypanosomal activity of **5** against *Trypanosoma brucei brucei* 52
Figure S50 Antitrypanosomal activity of **1** against *Trypanosoma evansi* 53
Figure S51 Antitrypanosomal activity of **5** against *Trypanosoma evansi* 53
Figure S52 Antileishmanial activity of **1** against *Leishmania mexicana promastigots* 54
Figure S53 Antileishmanial activity of **5** against *Leishmania mexicana promastigots* 54
Figure S54 Antileishmanial activity of **1** against *Leishmania mexicana amastigots* 55
Figure S55 Antileishmanial activity of **5** against *Leishmania mexicana amastigots* 55
Figure S57 Cytotoxic activity of **5** against Jurkat cell line 57
Figure S58 Effect on fibroblasts morphology of **5** at 5μM. Mitochondria are visualized using the actin cytoskeleton with Phalloidin (green), and nuclei with DAPI (blue). 58
Figure S59 Effect on HeLa morphology of **5** at 125μM. Mitochondria are visualized using MitoTracker Red CMXRos (red), the actin cytoskeleton with Phalloidin (green), and nuclei with DAPI (blue). 59
Figure S60 Cytotoxic activity of **1** against Jurkat cell line 60
Figure 61 Cytotoxic activity of **1** against Jurkat cell line 61;

Figure S62 Effect on fibroblasts morphology of **1** at 5µM. Mitochondria are visualized using the actin cytoskeleton with Phalloidin (green), and nuclei with DAPI (blue). 62

Figure S63 Effect on HeLa morphology of **1** at 125µM. Mitochondria are visualized using MitoTracker Red CMXRos (red), the actin cytoskeleton with Phalloidin (green), and nuclei with DAPI (blue)... 63

Figure S64 Cytotoxic activity of **3** against Jurkat cell line 64

Figure S65 Cytotoxic activity of **3** against Jurkat cell line 65

Figure S66 Effect on fibroblasts morphology of **3** at 125µM. Mitochondria are visualized using MitoTracker Red CMXRos (red), the actin cytoskeleton with Phalloidin (green), and nuclei with DAPI (blue)... 66

Figure S67 Effect on HeLa cells morphology of **3** at 25µM. Mitochondria are visualized using MitoTracker Red CMXRos (red), the actin cytoskeleton with Phalloidin (green), and nuclei with DAPI (blue)... 67

Figure 68 Cytotoxic activity of **7** against Jurkat cell line 68

Figure 69 Cytotoxic activity of **7** against Jurkat cell line 69

Figure S70 Effect on fibroblasts morphology of **7** at 25µM. Mitochondria are visualized using MitoTracker Red CMXRos (red), the actin cytoskeleton with Phalloidin (green), and nuclei with DAPI (blue)... 70

Figure S71 Effect on HeLa cells morphology of **3** at 125µM. Mitochondria are visualized using MitoTracker Red CMXRos (red), the actin cytoskeleton with Phalloidin (green), and nuclei with DAPI (blue)... 71

Figure S72 RTL-W1 cell line viability after treatment with **5** and almarBlue (AB) fluorescent indicator. 72

Figure S73 RTL-W1 cell line viability after treatment with **1** and almarBlue (AB) fluorescent indicator. 73

Figure S74 RTL-W1 cell line viability after treatment with **5** and 5-Carboxyfluorescein Diacetate Acetoxymethyl Ester (CFDA-AM) fluorescent indicator 74

Figure S75 RTL-W1 cell line viability after treatment with **1** and 5-Carboxyfluorescein Diacetate Acetoxymethyl Ester (CFDA-AM) fluorescent indicator 75

Figure S76 RTL-W1 cell line viability after treatment with **5** and neutral red (NR) fluorescent indicator 76

Figure S77 RTL-W1 cell line viability after treatment with **1** and neutral red (NR) fluorescent indicator 77

HPLC

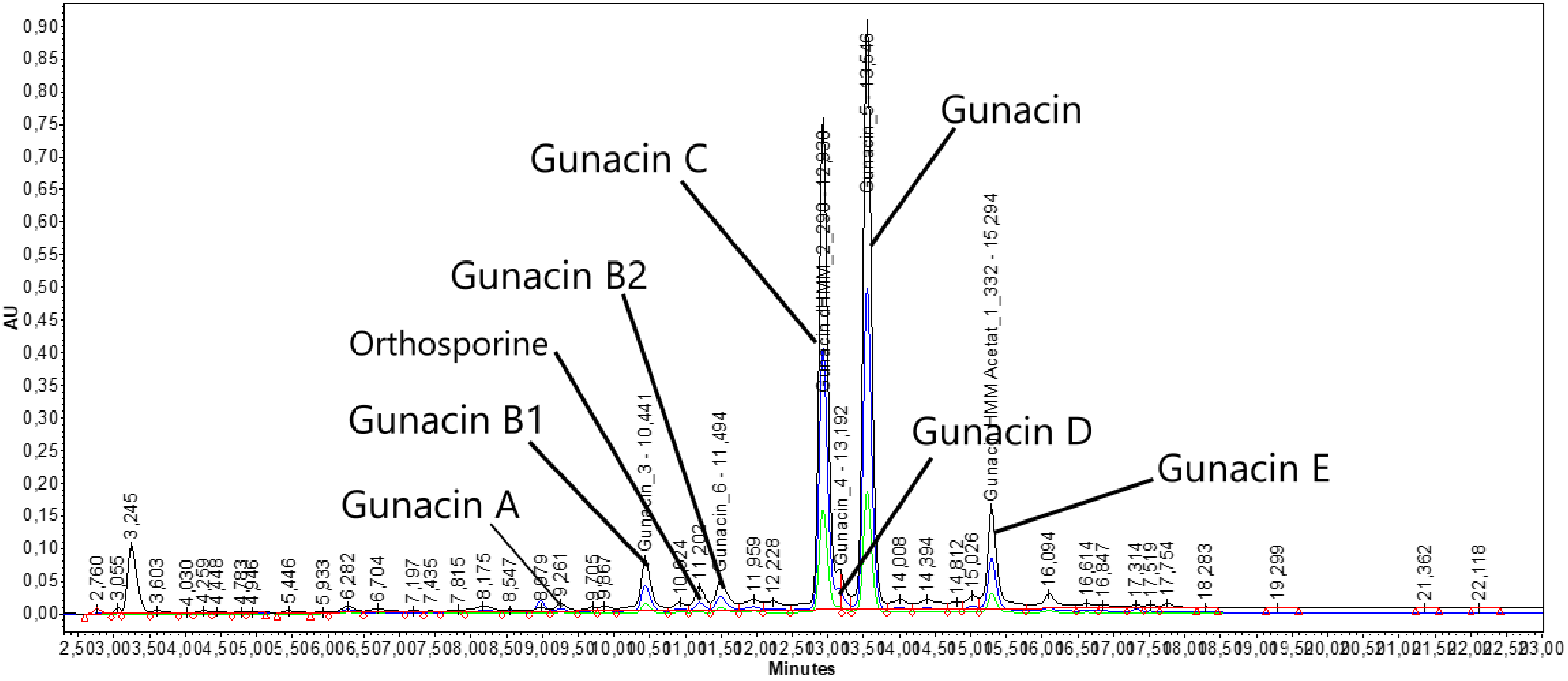

Figure S1 Chromatograph of crude extract

Mass spectroscopy data of compounds 1-8

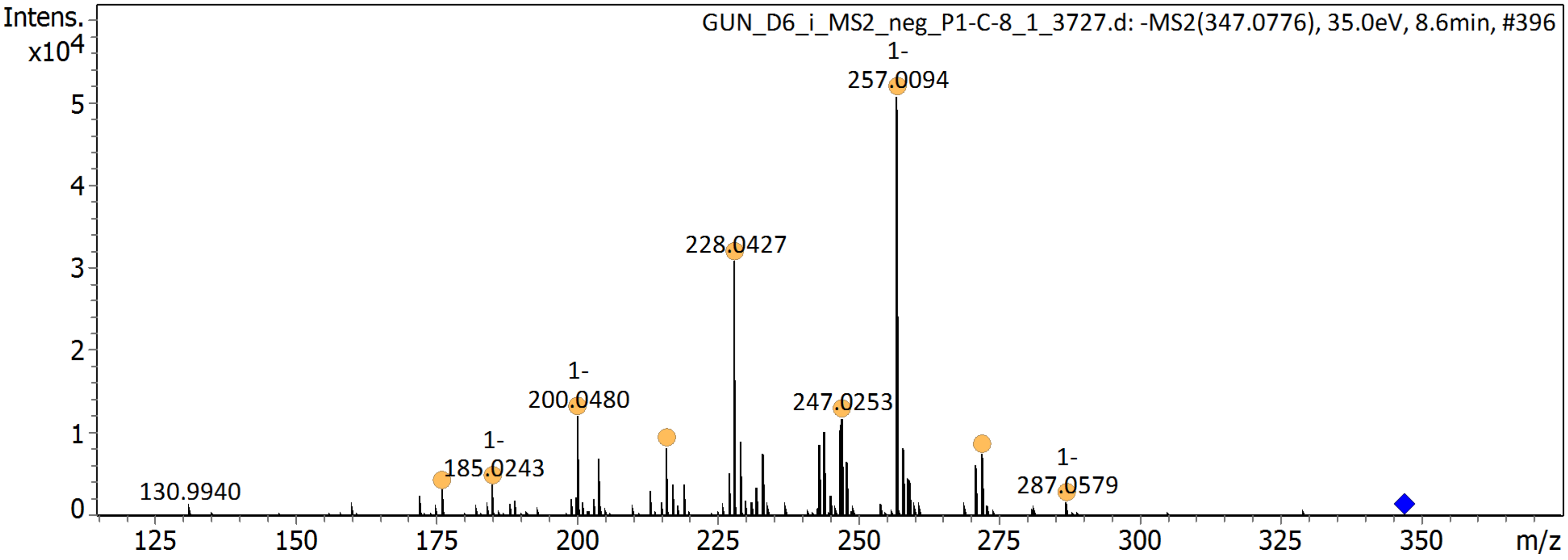

Figure S2 Negative MS/MS spectrum of **1**

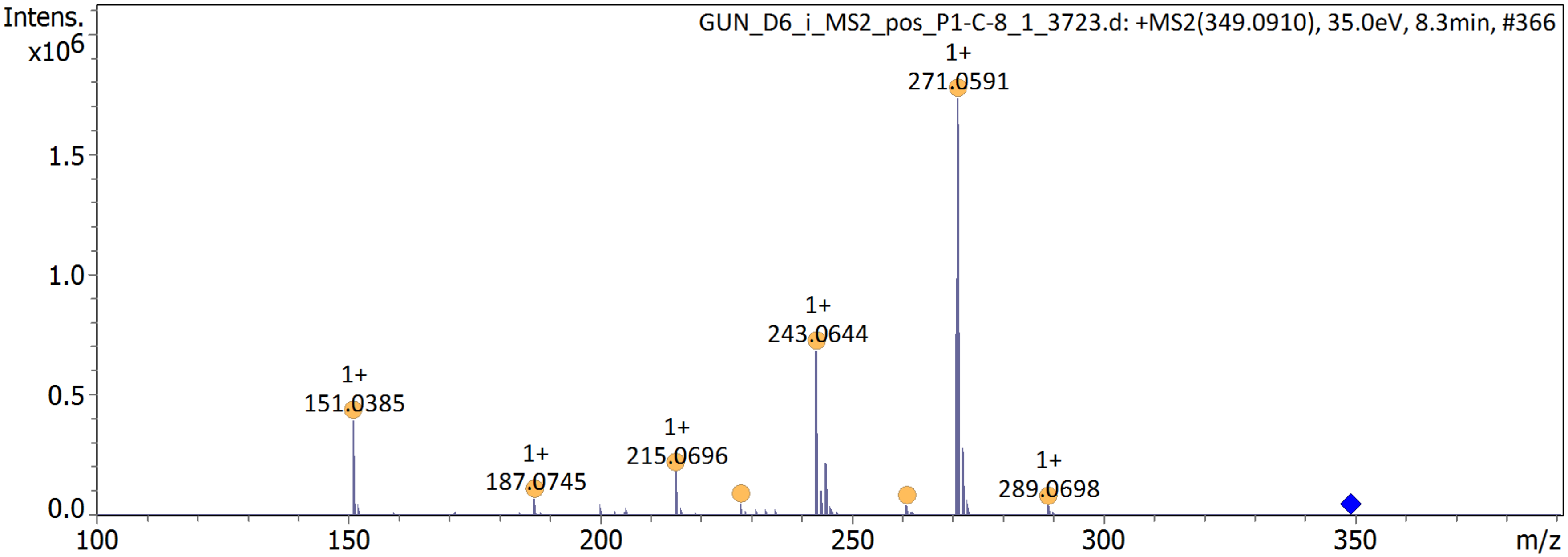

Figure S3 Positive MS/MS spectrum of **1**

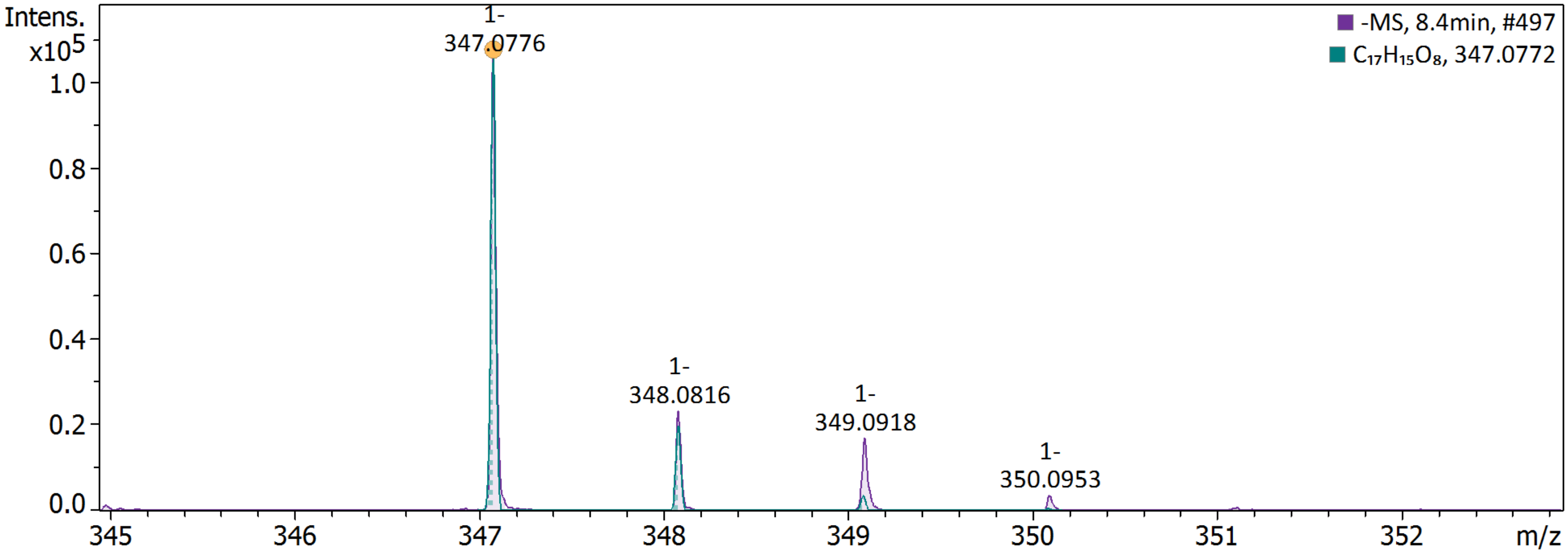

Figure S4 Negative HRESIMS spectrum of **1**

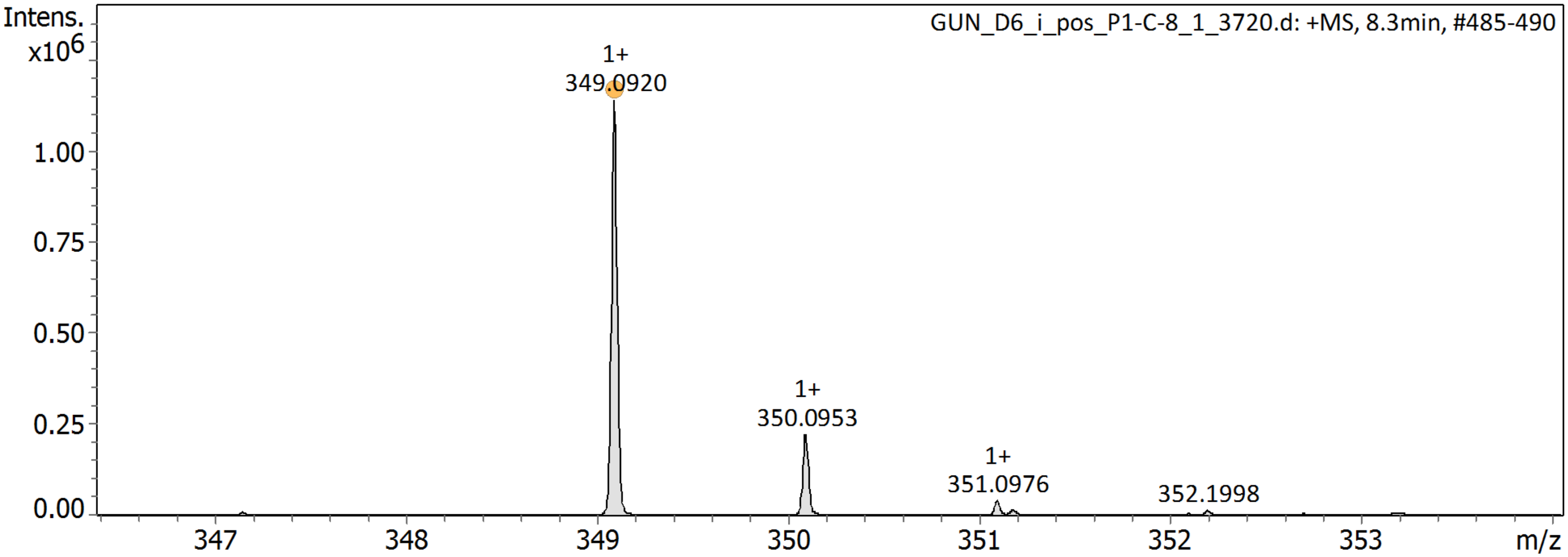

Figure S5 Positive HRESIMS spectrum of 1

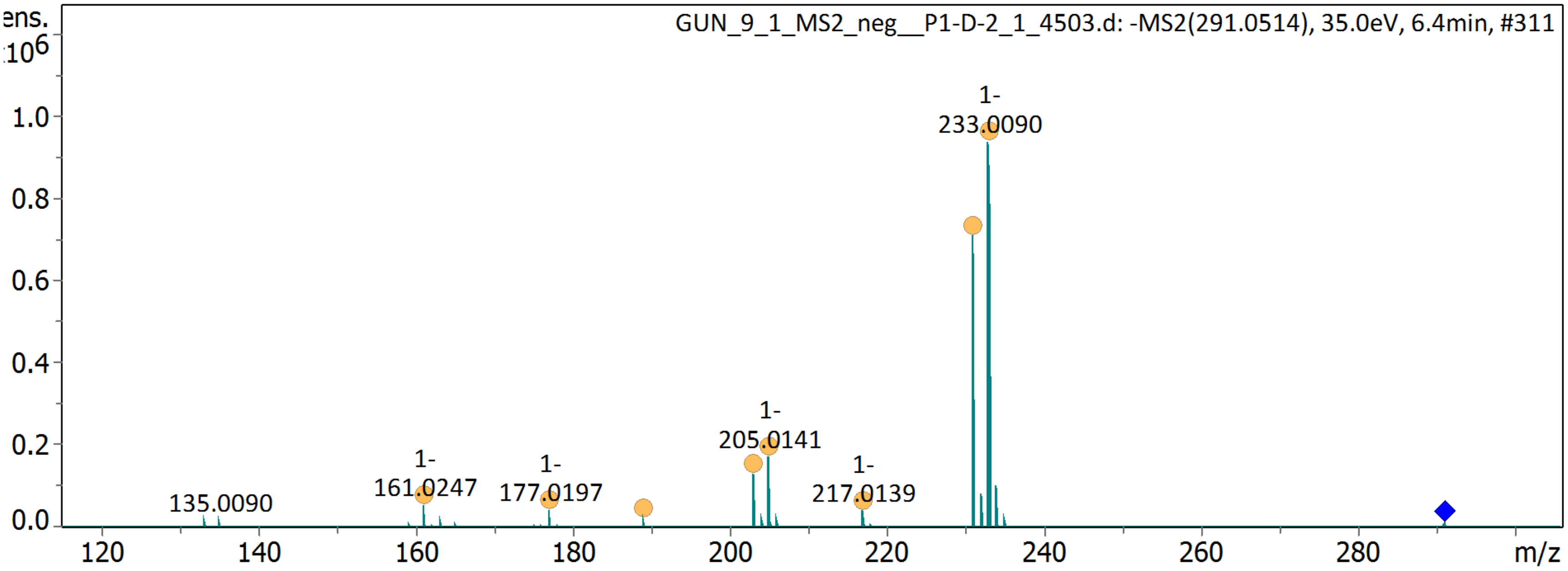

Figure S6 Negative MS/MS spectrum of 2

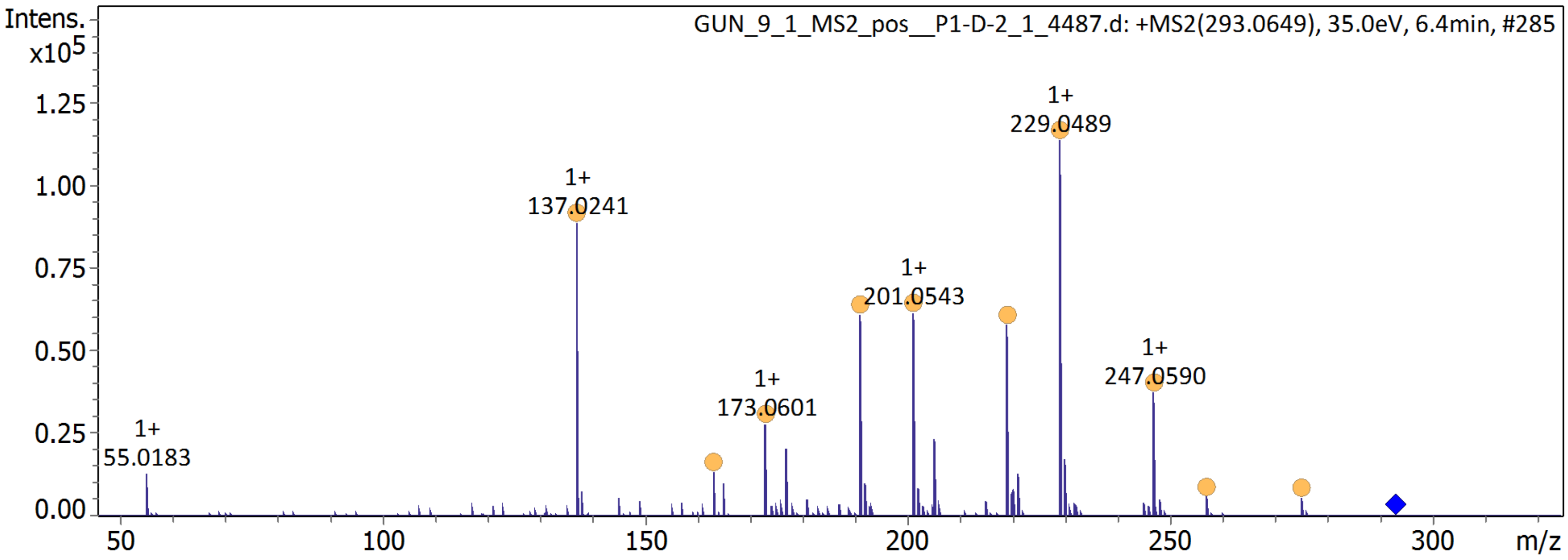

Figure S7 Positive MS/MS spectrum of **2**

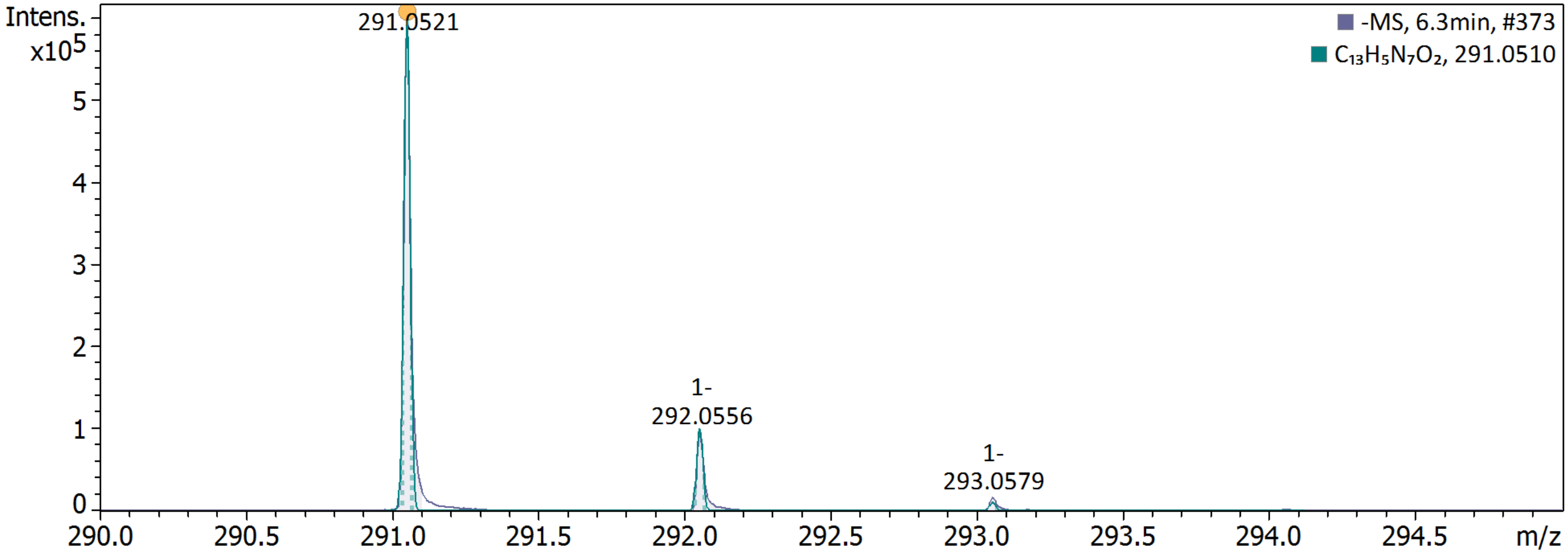

Figure S8 Negative HRESIMS spectrum of **2**

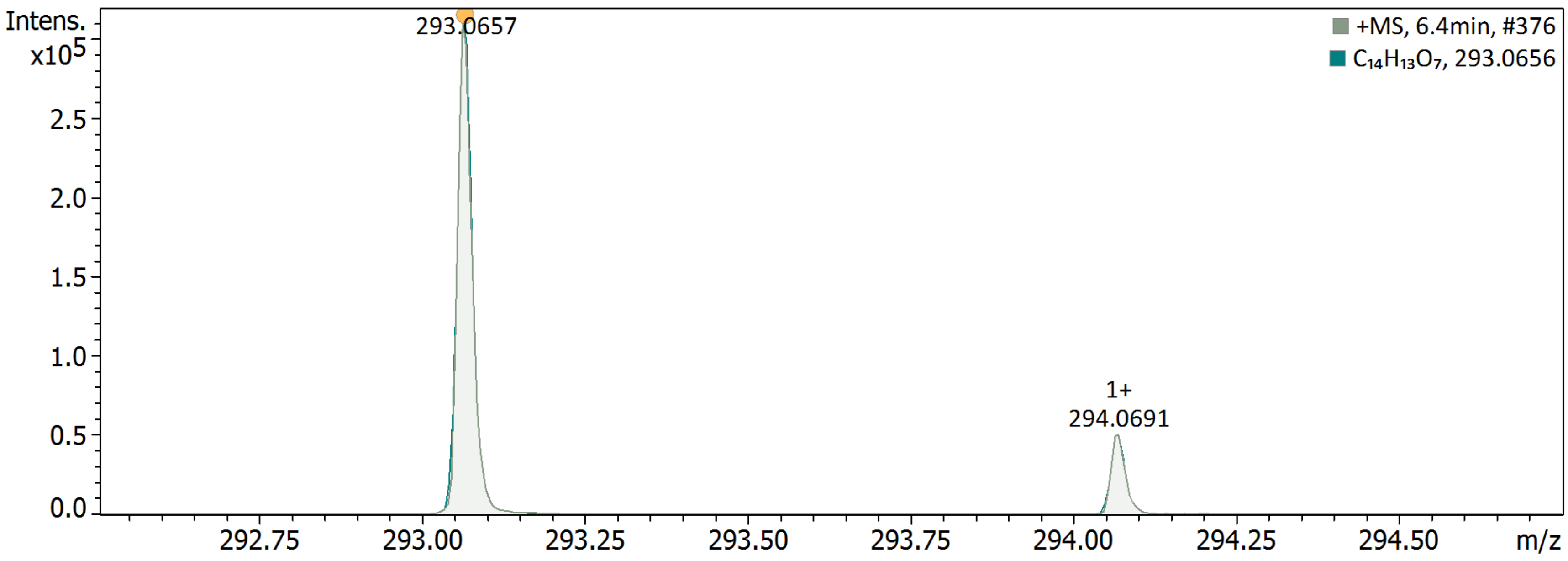

Figure S9 Positive HRESIMS spectrum of **2**

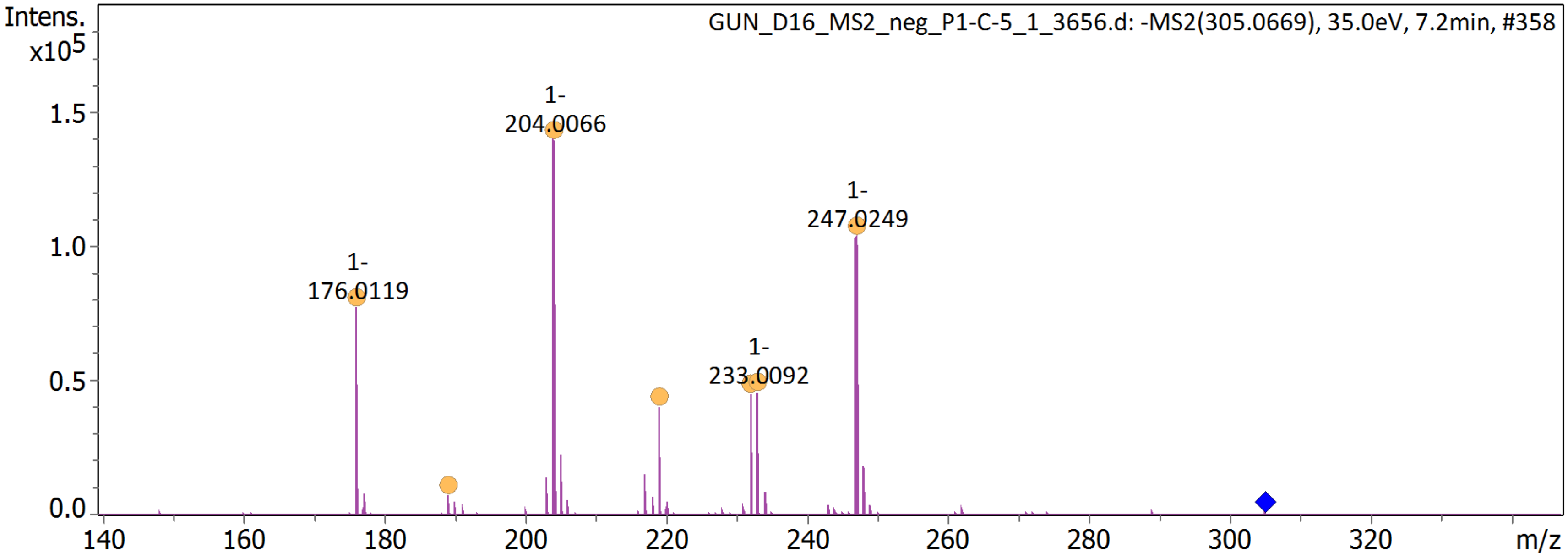

Figure S10 Negative MS/MS spectrum of **3**

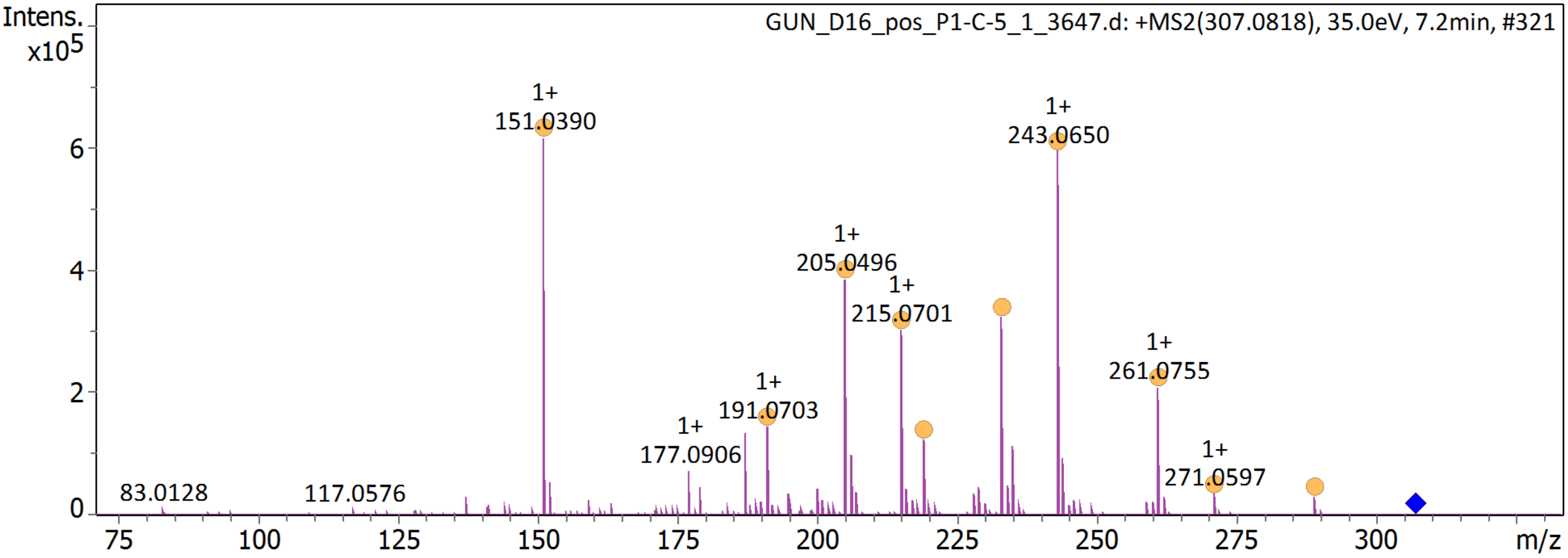

Figure S11 Positive MS/MS spectrum of **3**

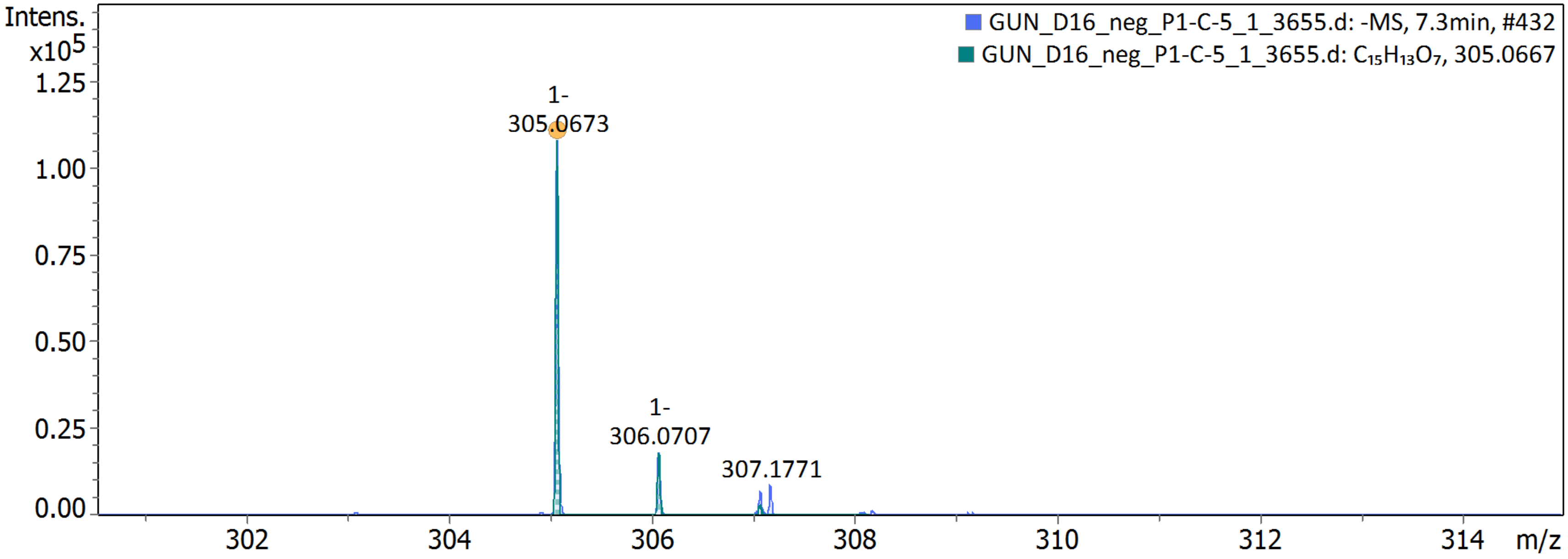

Figure S12 Negative HRESIMS spectrum of **3**

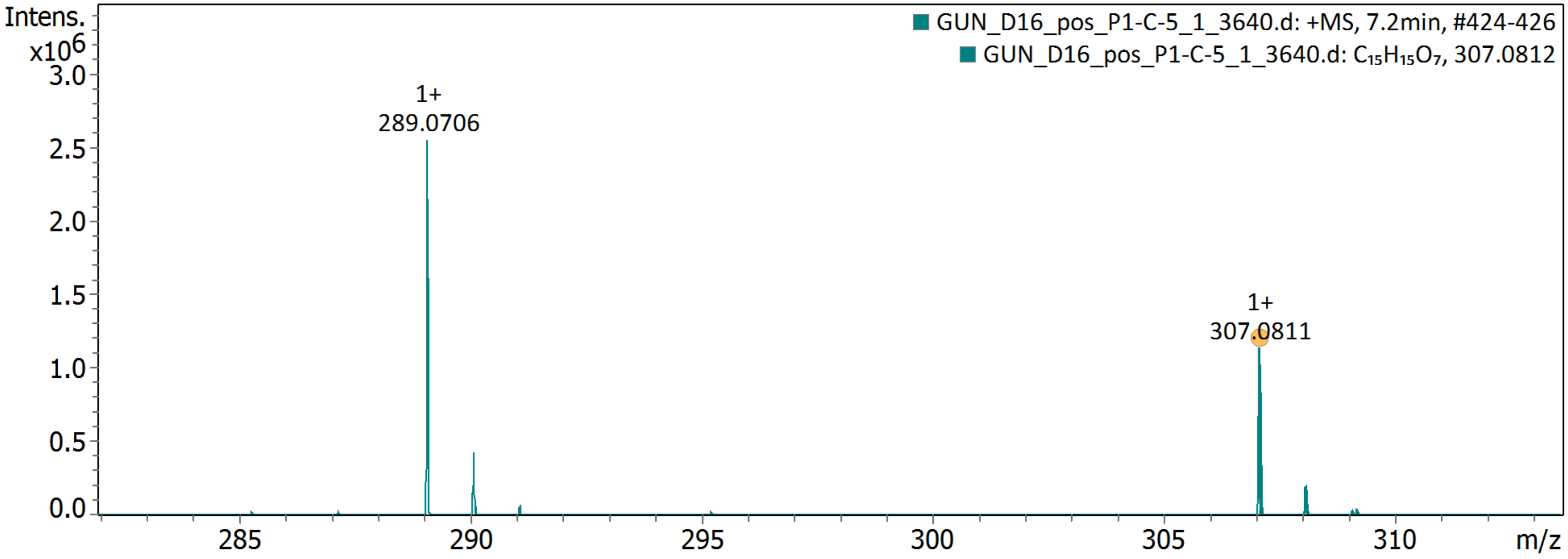

Figure S13 Positive HRESIMS spectrum of **3**

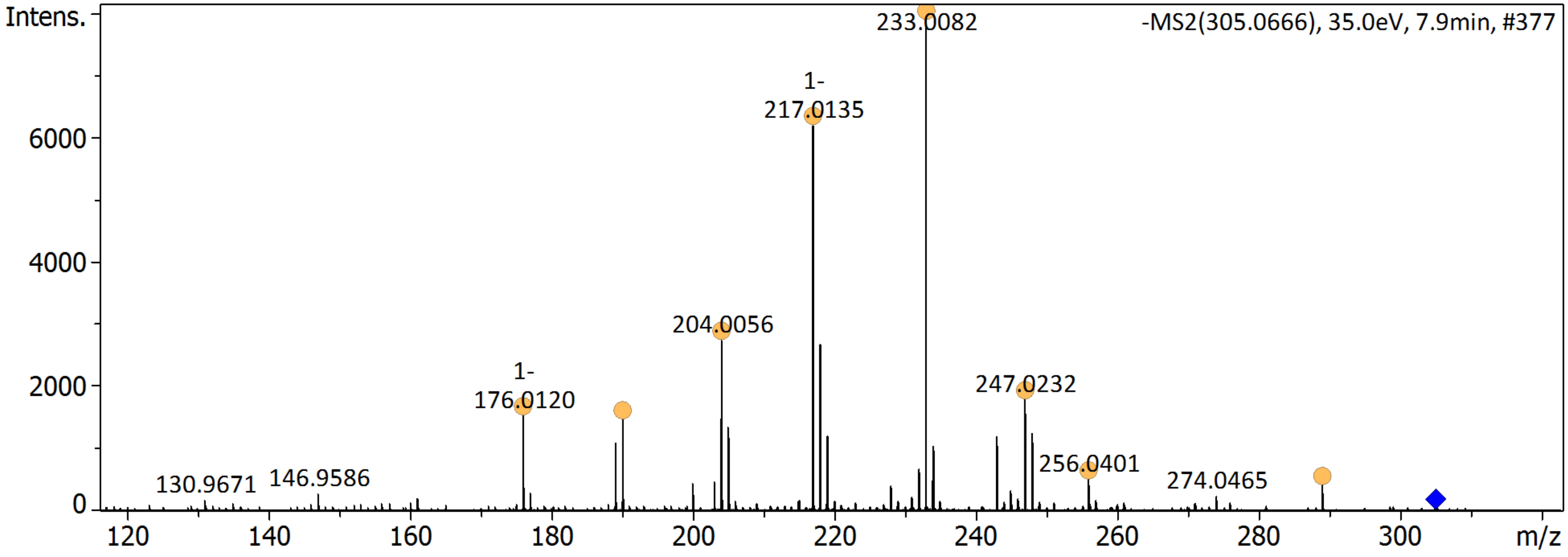

Figure S14 Negative MS/MS spectrum of **4**

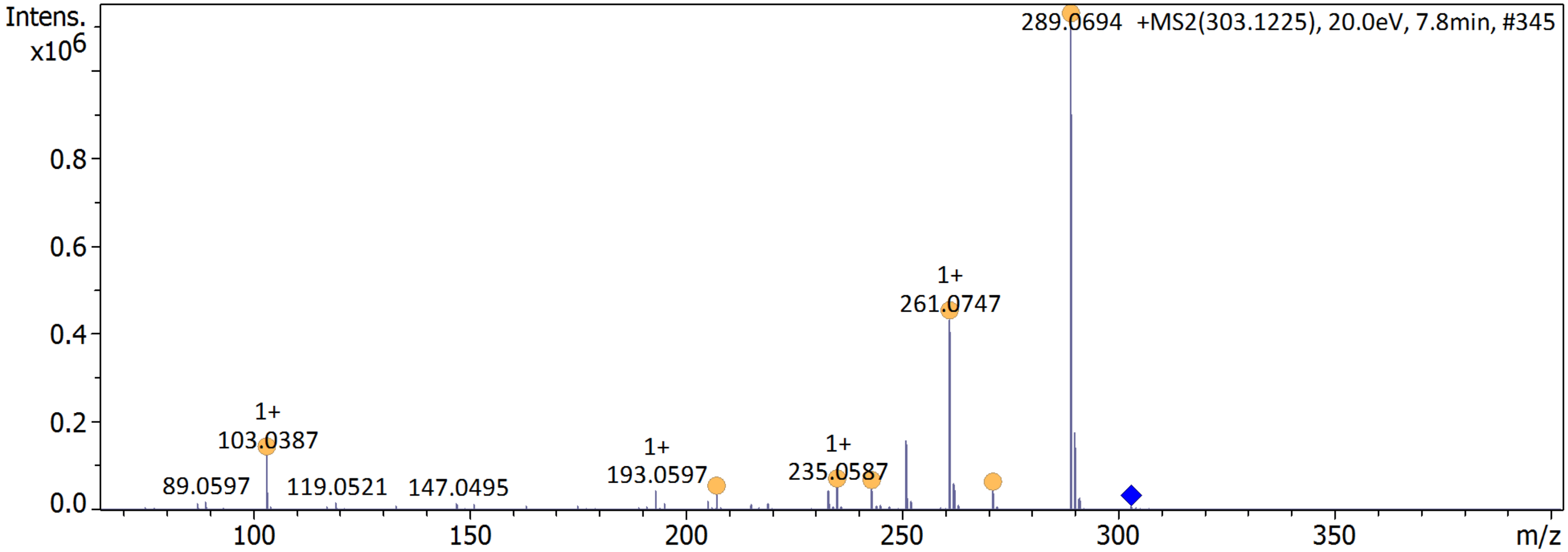

Figure S15 Positive MS/MS spectrum of **4**

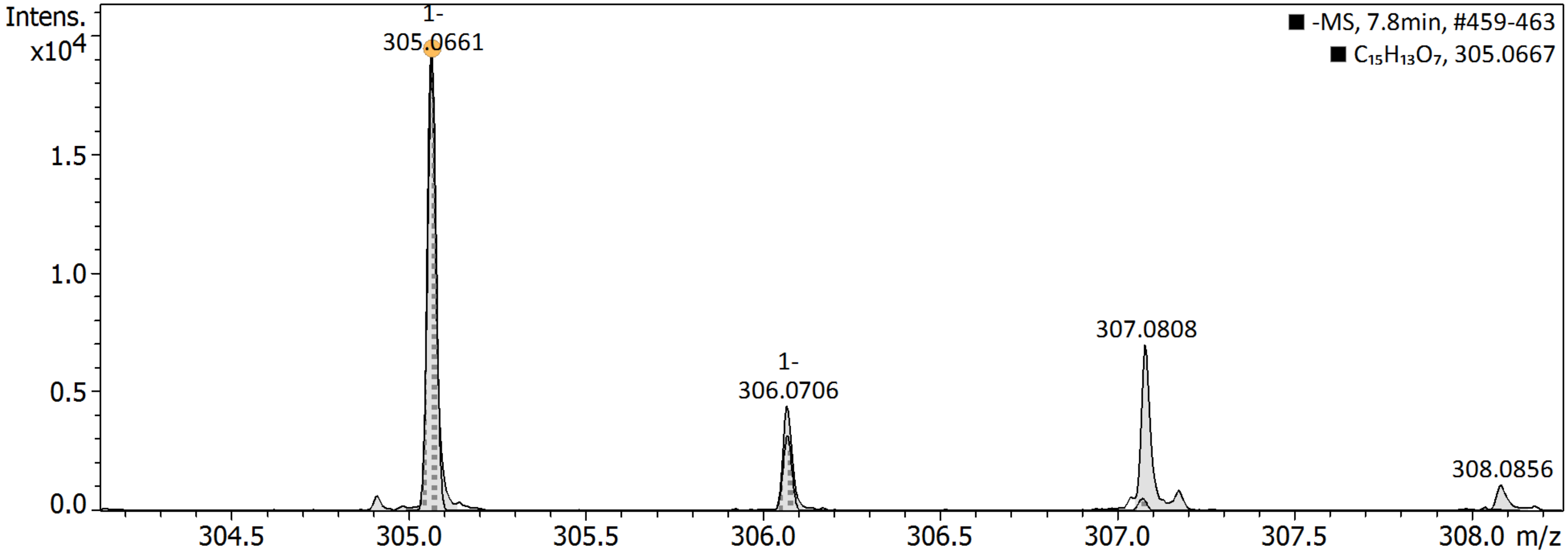

Figure S16 Negative HRESIMS spectrum of **4**

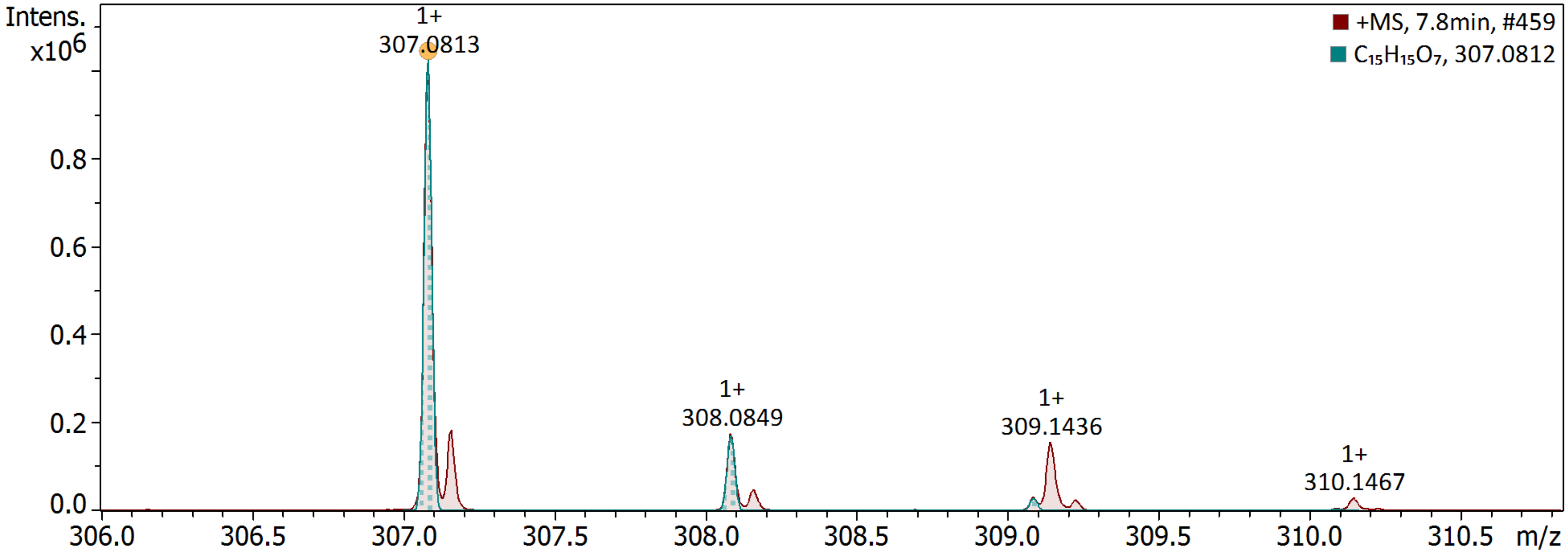

Figure S17 Positive HRESIMS spectrum of **4**

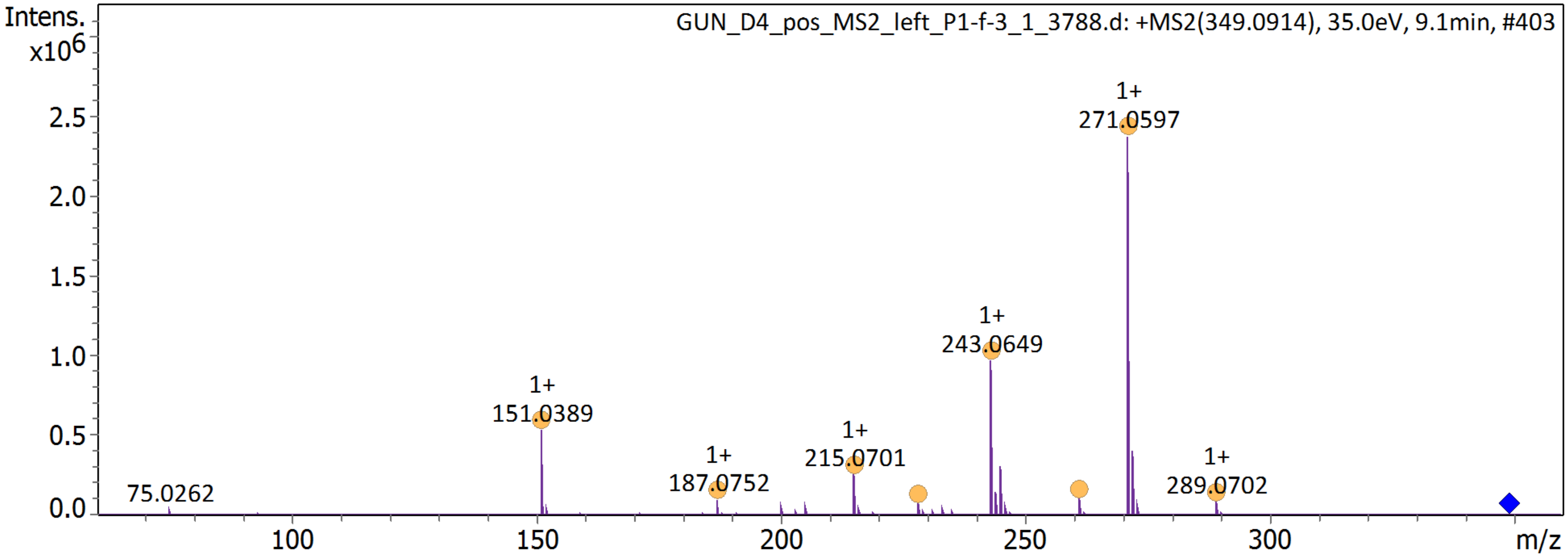

Figure S18 Positive MS/MS spectrum of **5**

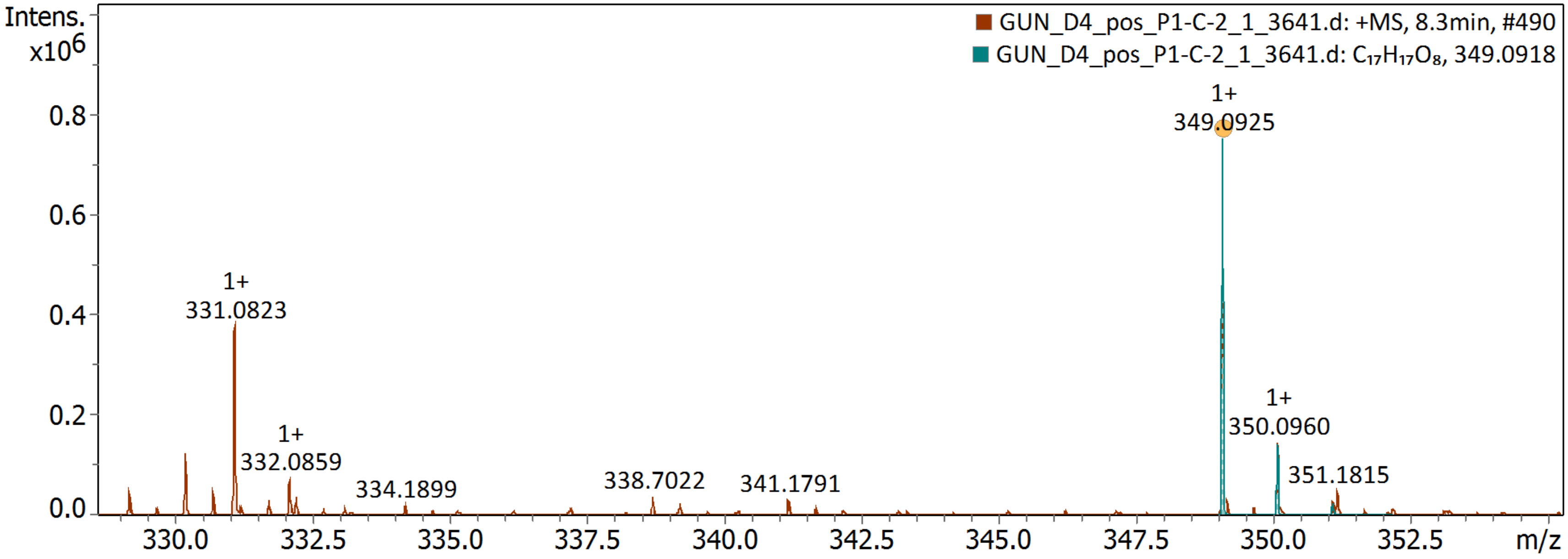

Figure S19 Positive HRESIMS spectrum of **5**

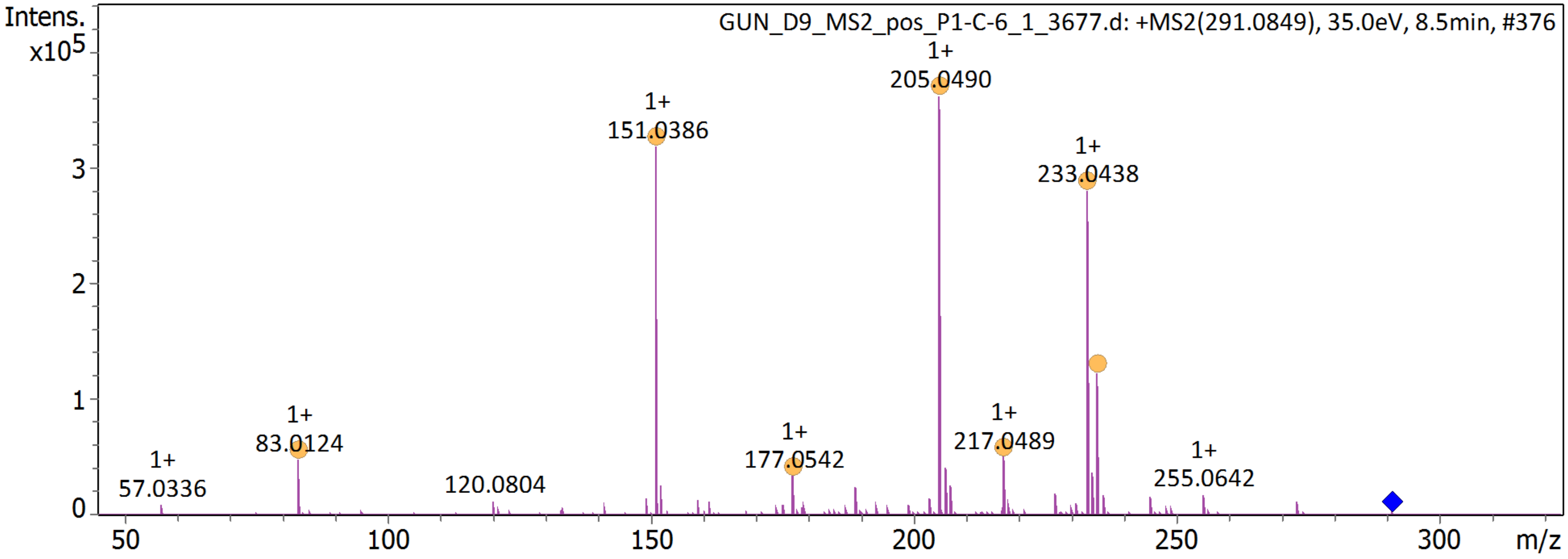

Figure S20 Positive MS/MS spectrum of **6**

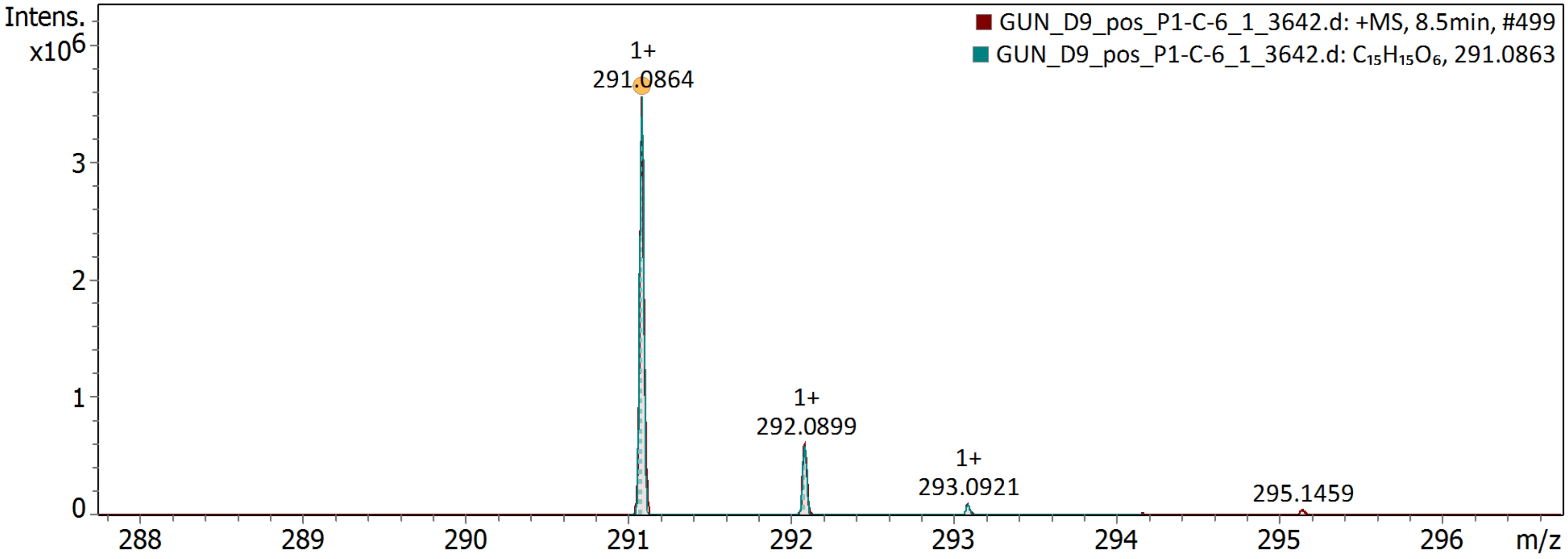

Figure S21 Positive HRESIMS spectrum of **6**

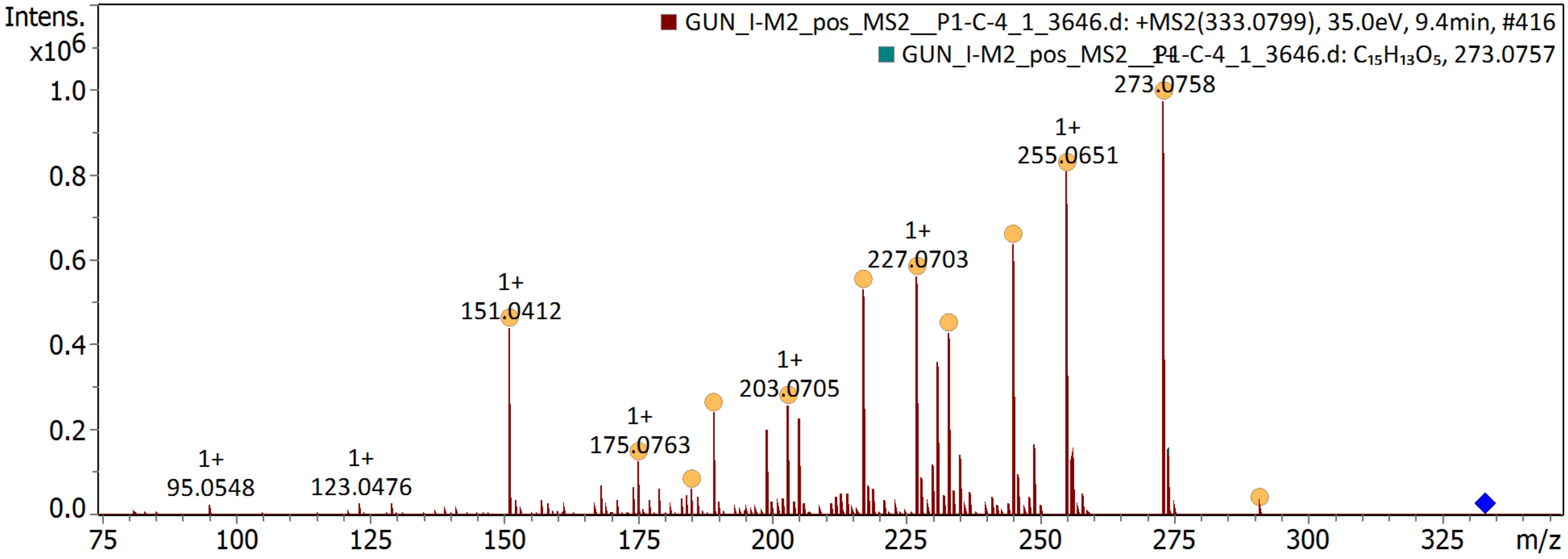

Figure S22 Positive MS/MS spectrum of **7**

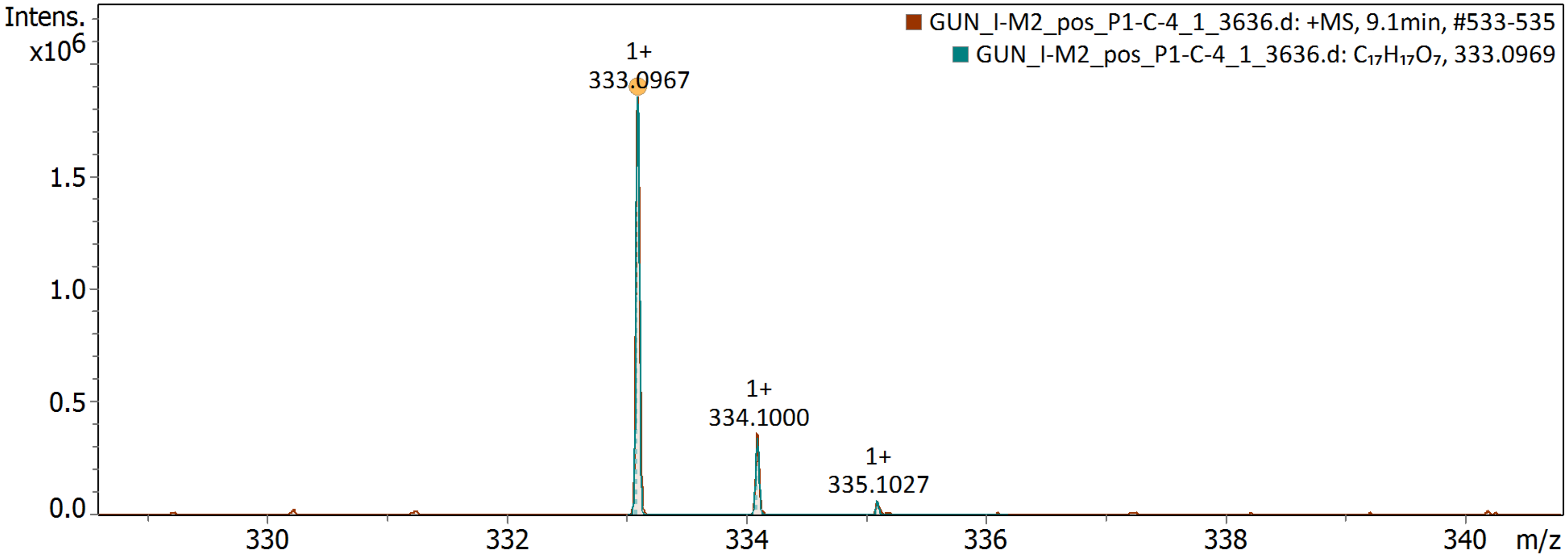

Figure S23 Positive HRESIMS spectrum of **7**

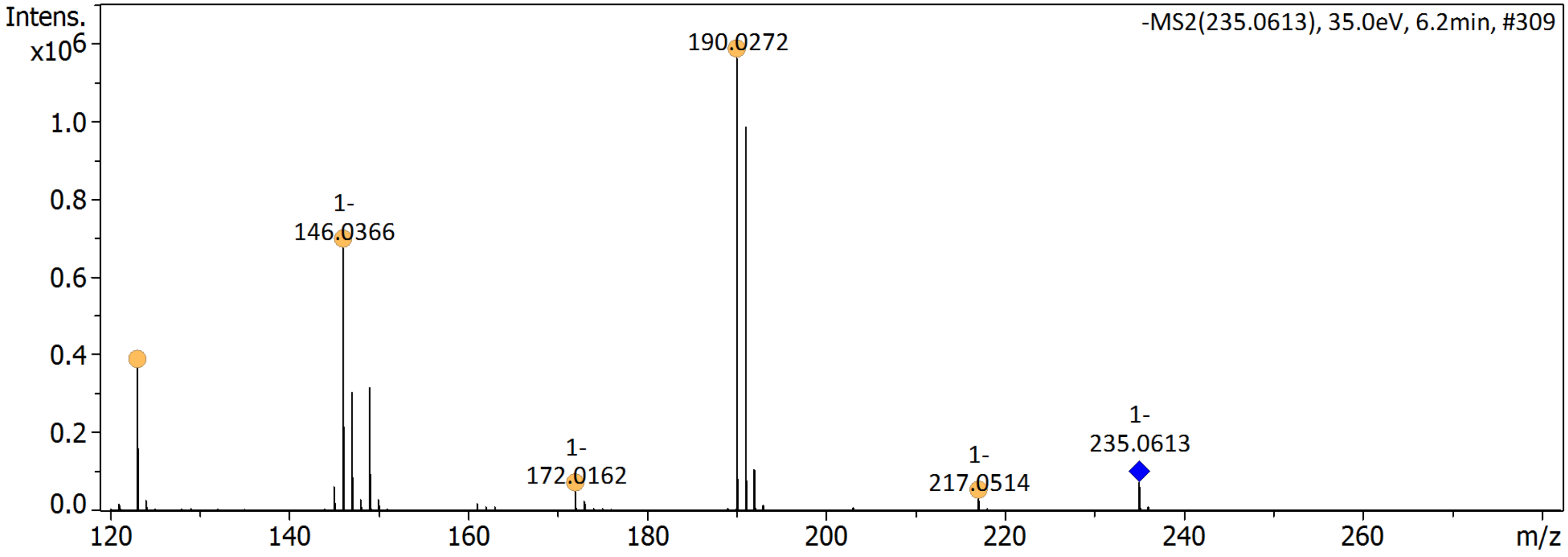

Figure S24 Negative MS/MS spectrum of **8**

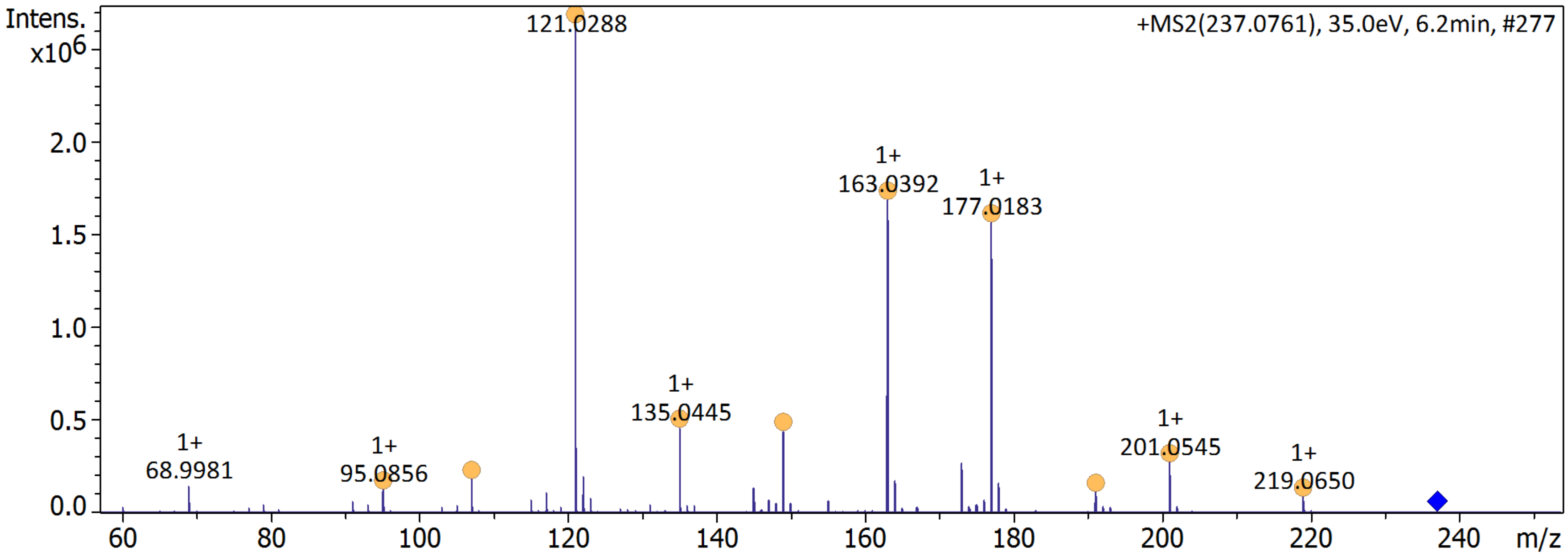

Figure S25 Positive MS/MS spectrum of **8**

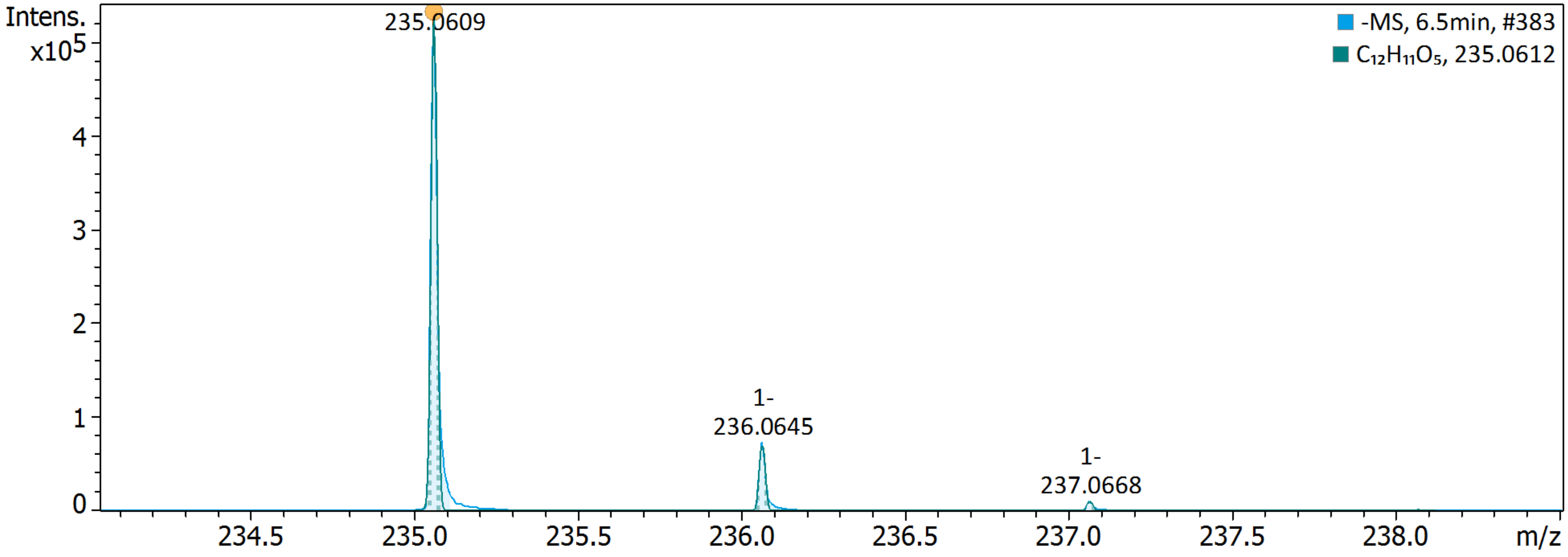

Figure S26 Negative HRESIMS spectrum of **8**

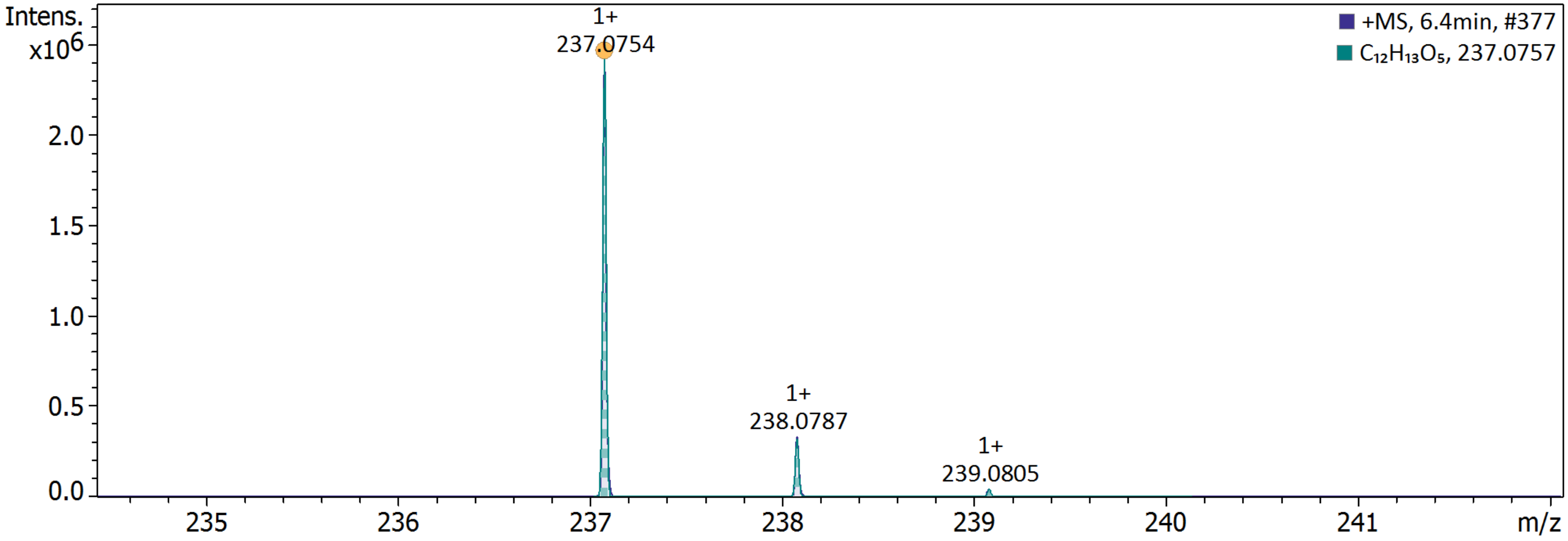

Figure S27 Positive HRESIMS spectrum of **8**

NMR data of compounds 1-8

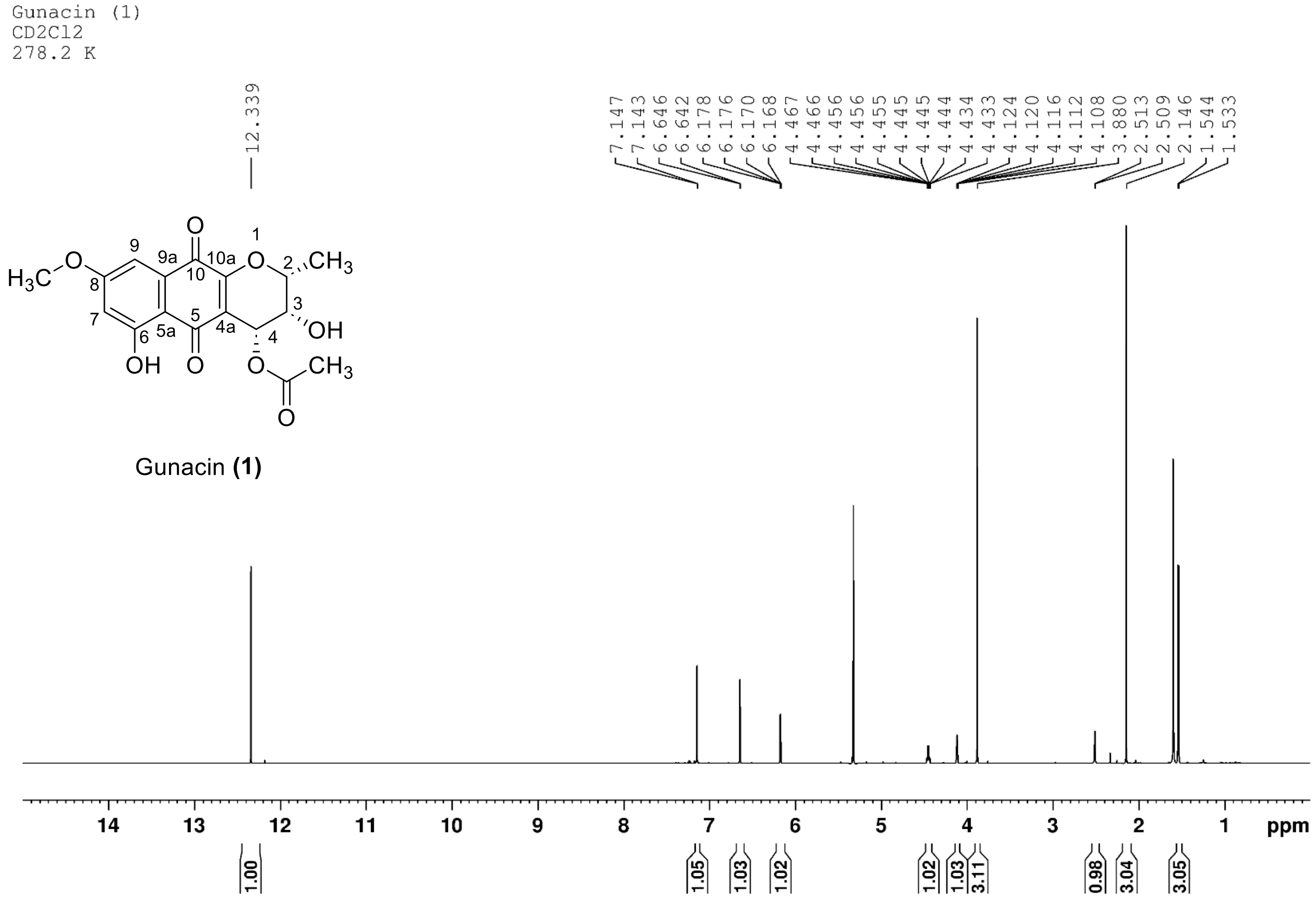

Figure S28 ^{1}H NMR spectrum of **1** (CD_2Cl_2, 278.2 K, 600.23 MHz)

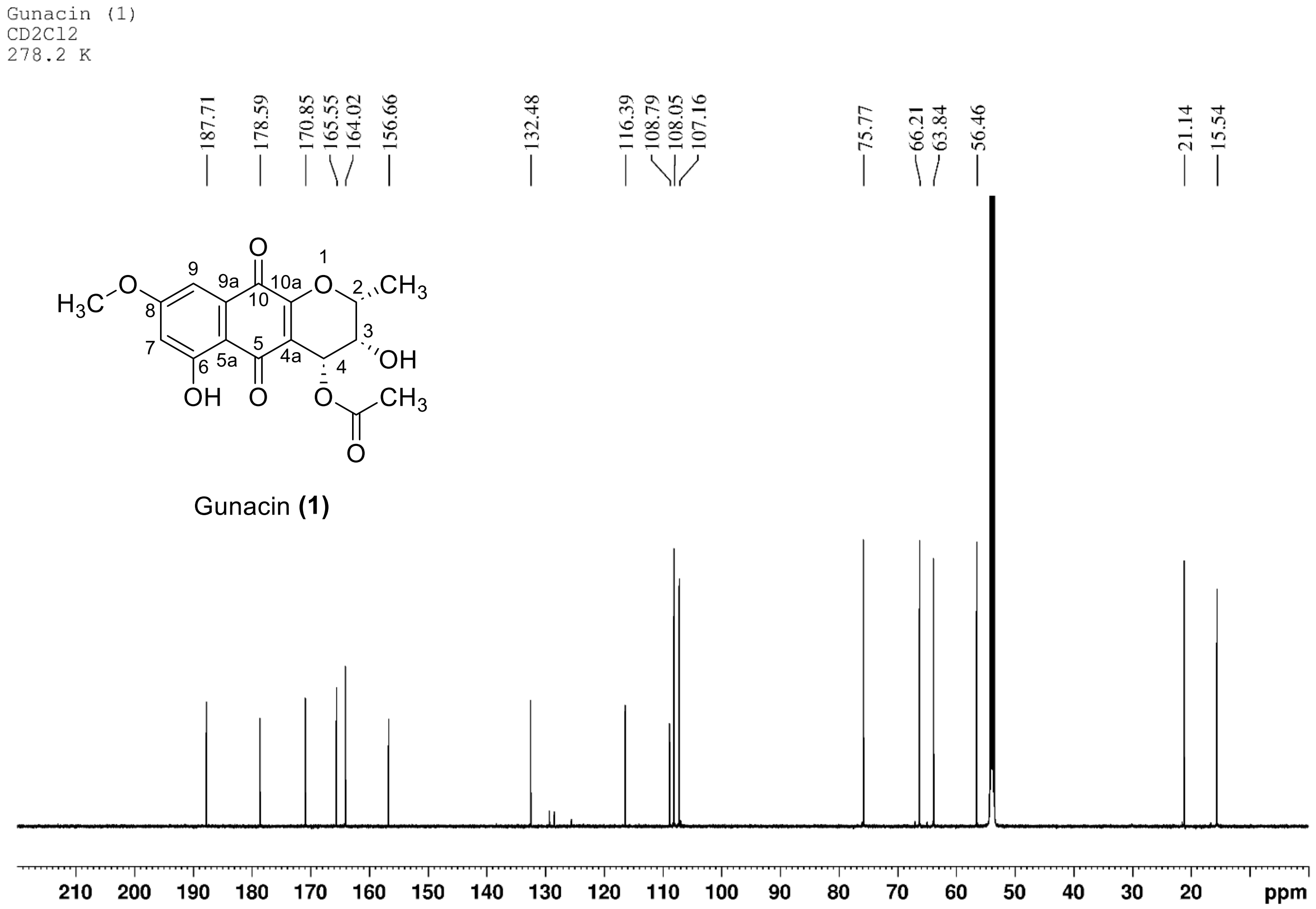

Figure S29 ^{13}C NMR spectrum of **1** (CD_2Cl_2, 278.2 K, 150.93 MHz)

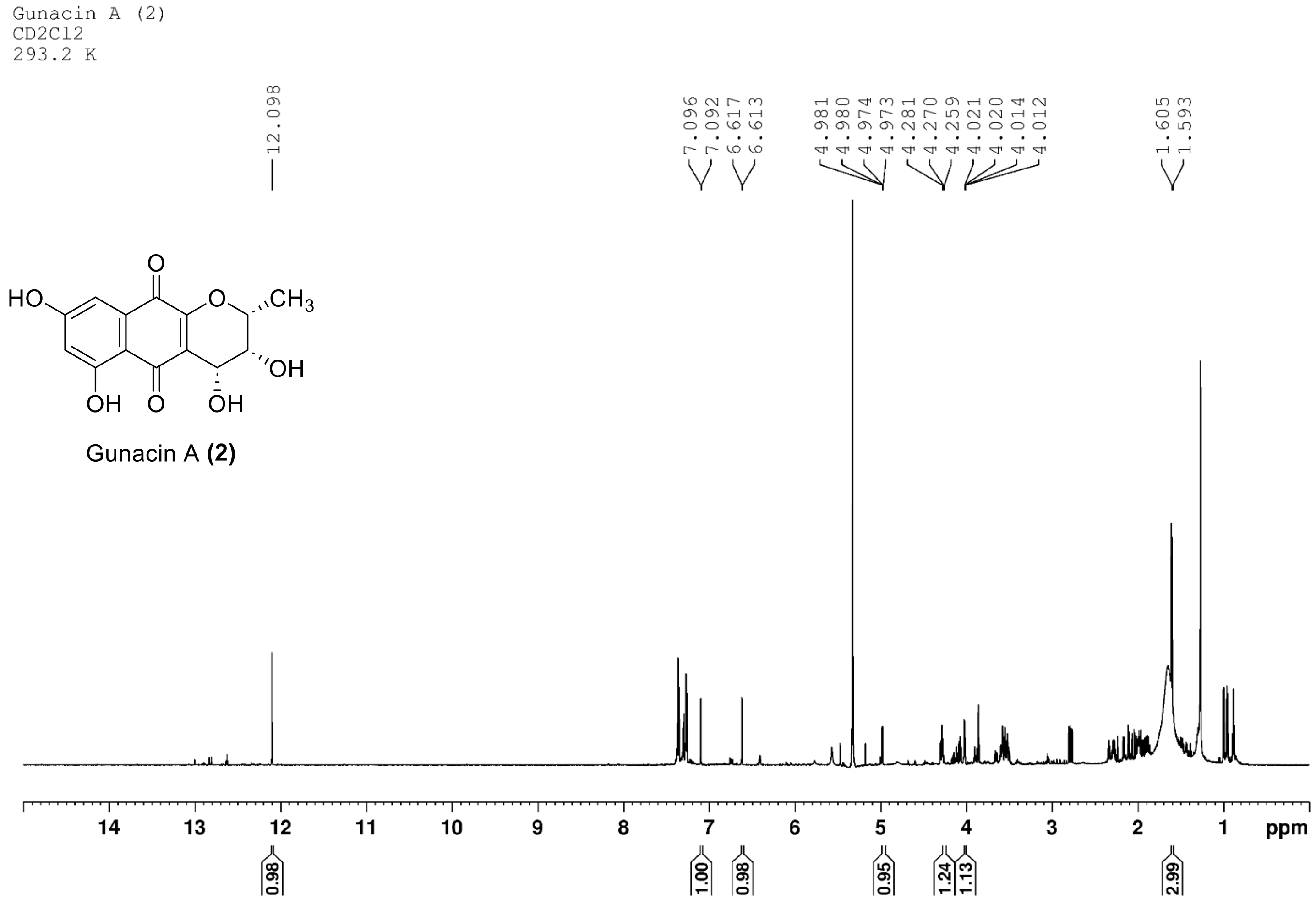

Figure S30 ^{1}H NMR spectrum of **2** (CD_2Cl_2, 293.2 K, 600.23 MHz)

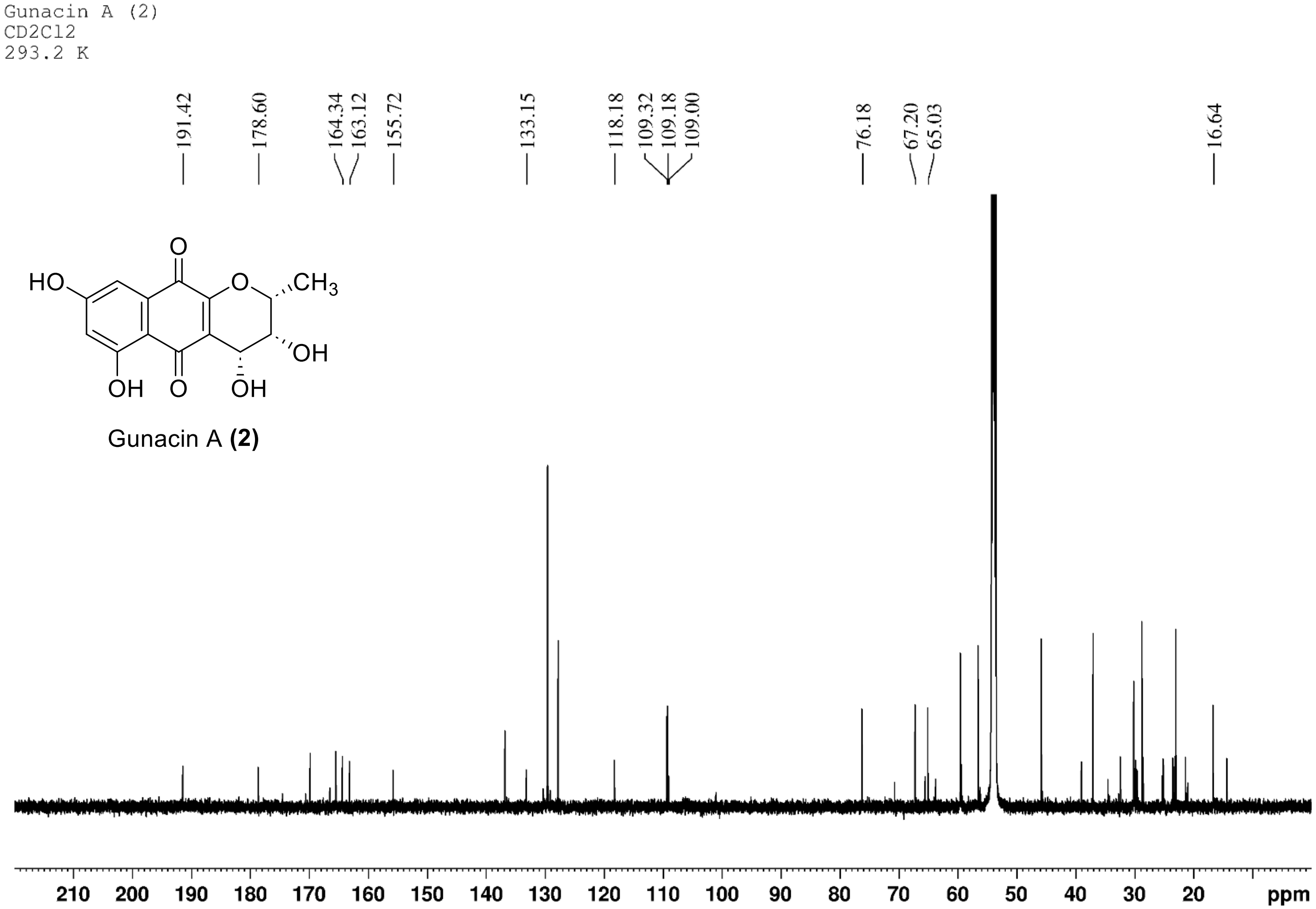

Figure S31 ^{13}C NMR spectrum of **2** (CD_2Cl_2, 293.2 K, 150.93 MHz)

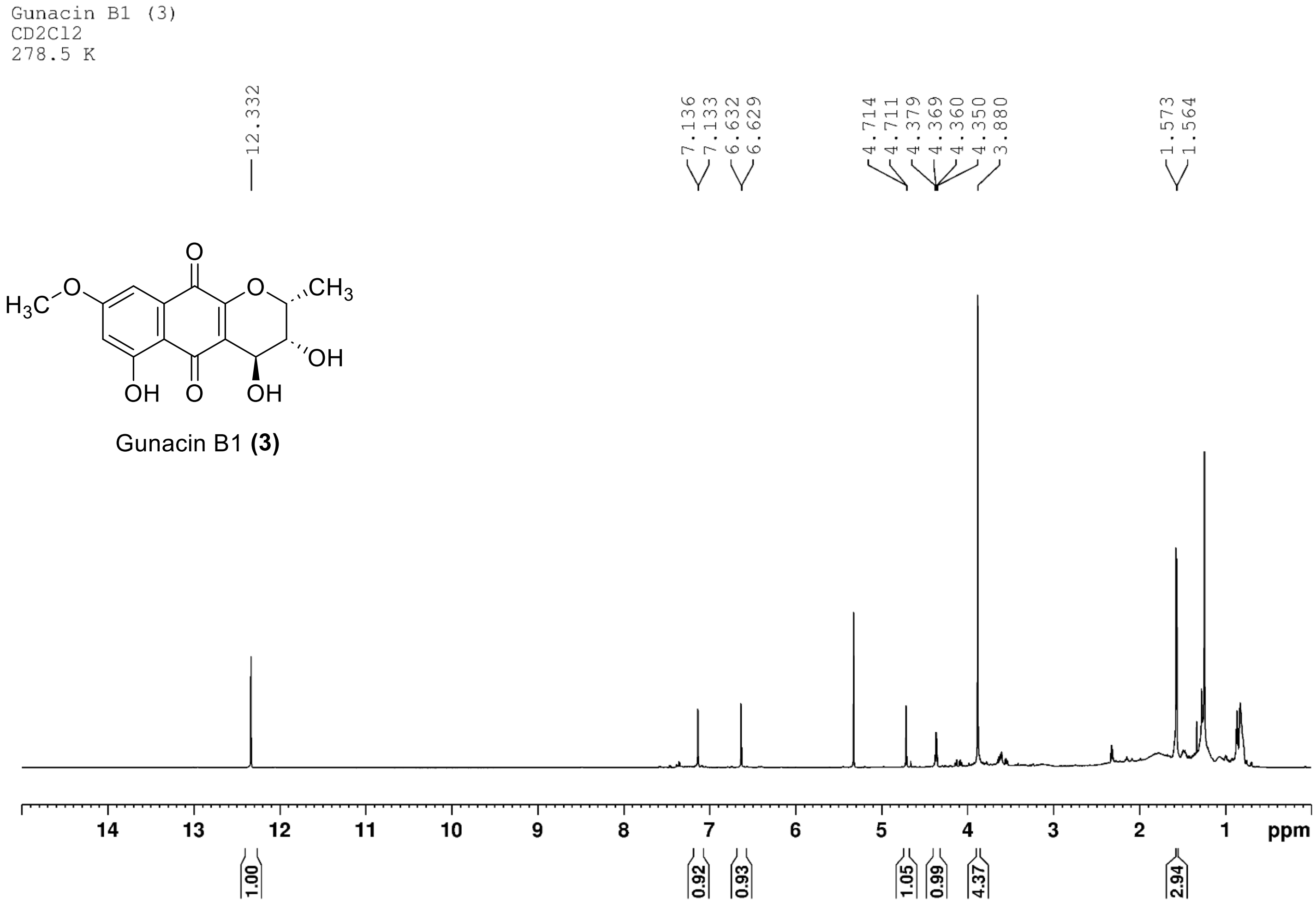

Figure S32 ^{1}H NMR spectrum of **3** (CD_2Cl_2, 278.2 K, 700.13 MHz)

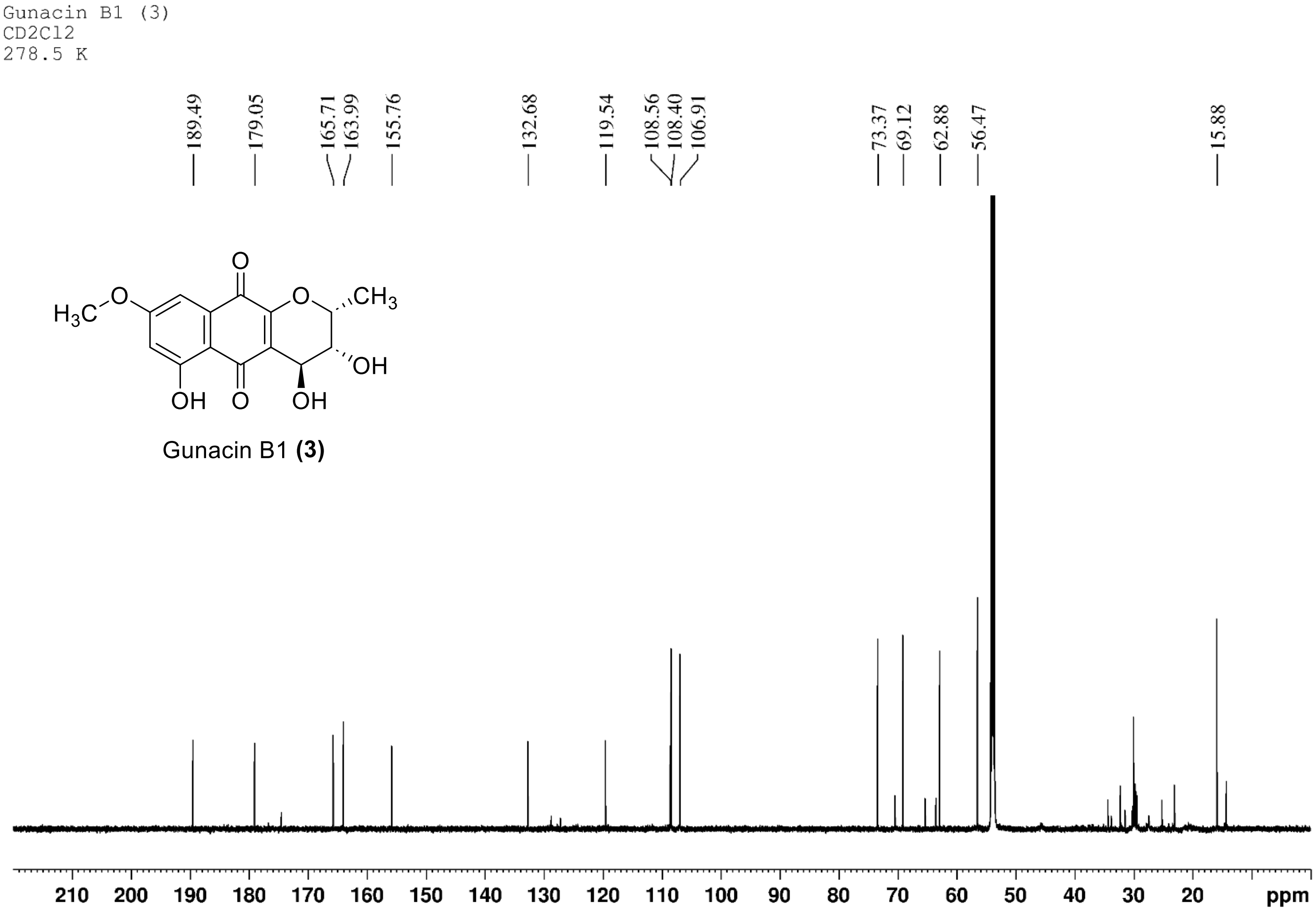

Figure S33 ^{13}C NMR spectrum of **3** (CD_2Cl_2, 278.2 K, 176.05 MHz)

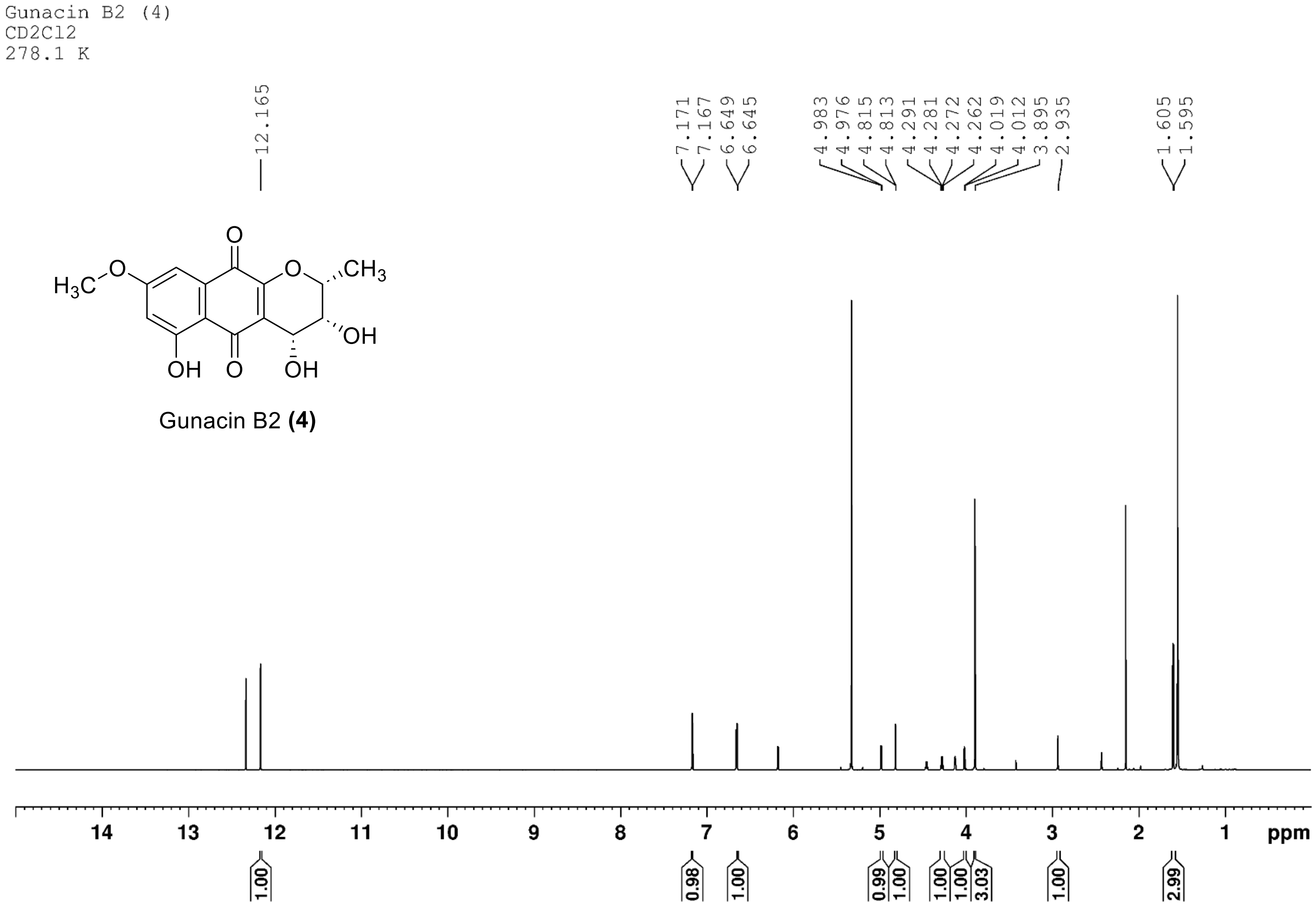

Figure S34 ^{1}H NMR spectrum of **4** (CD_2Cl_2, 293.2 K, 700.13 MHz)

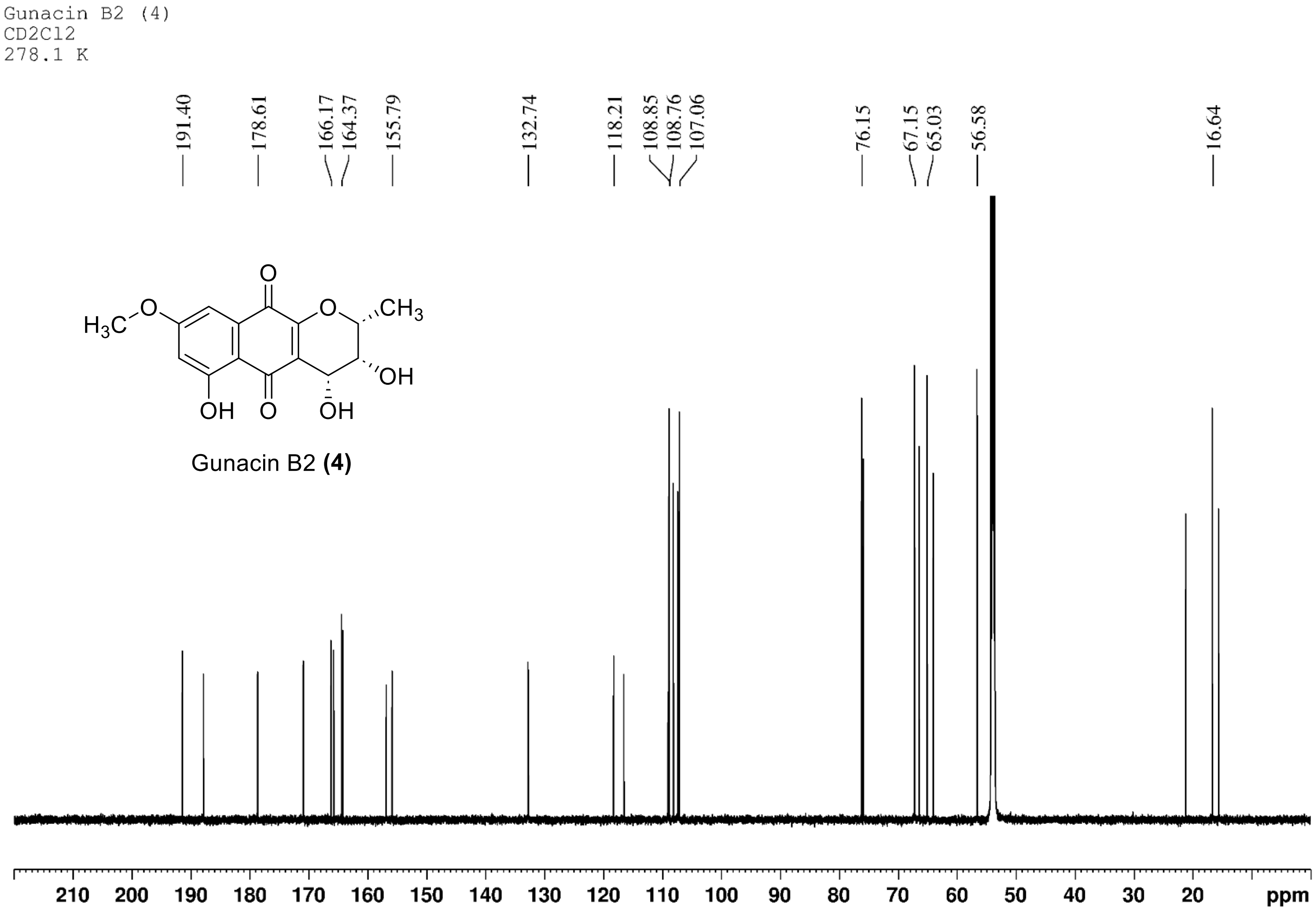

Figure S35 ^{13}C NMR spectrum of **4** (CD_2Cl_2, 293.2 K, 176.05 MHz)

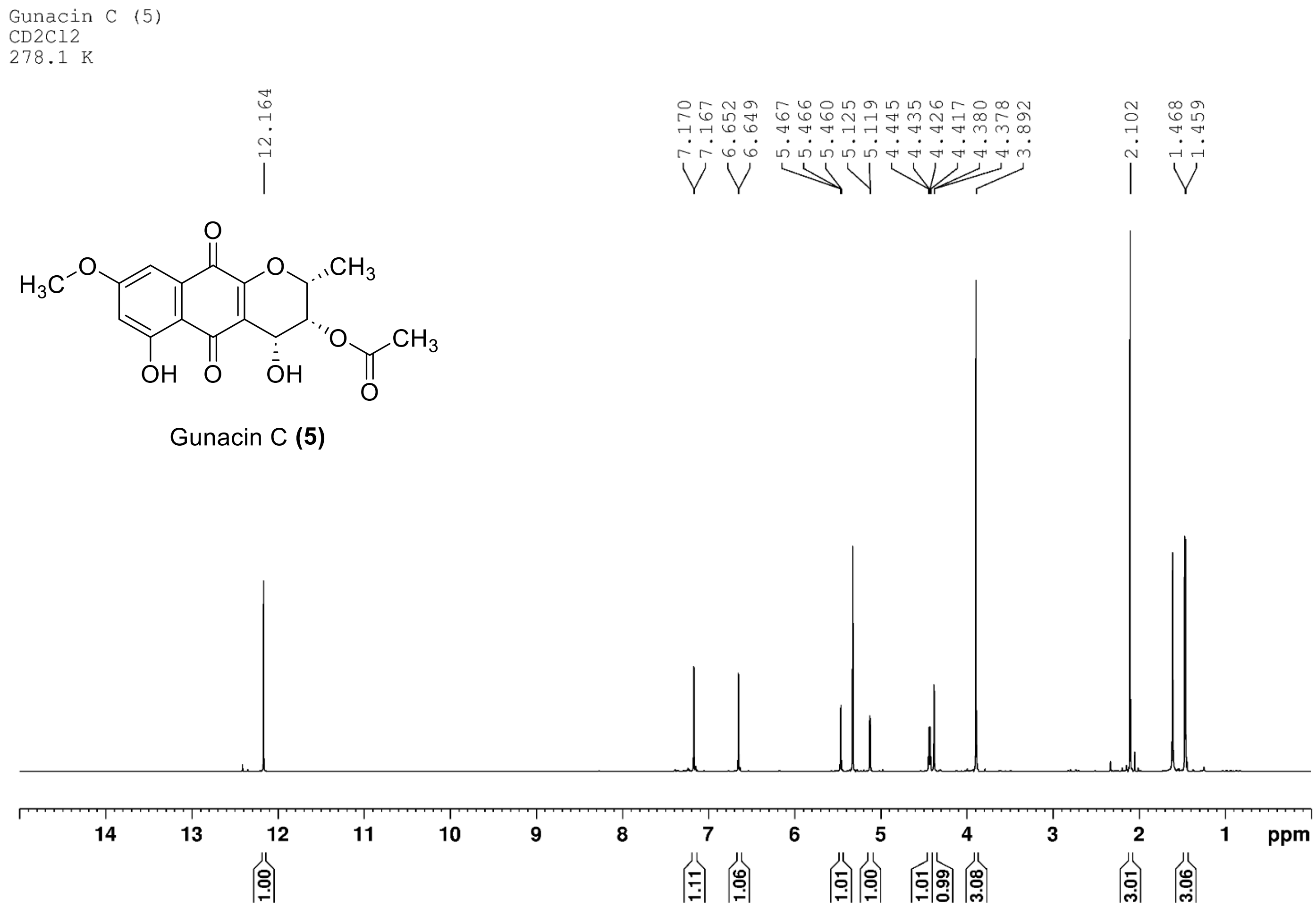

Figure S36 ^{1}H NMR spectrum of **5** (CD_2Cl_2, 278.2 K, 700.13 MHz)

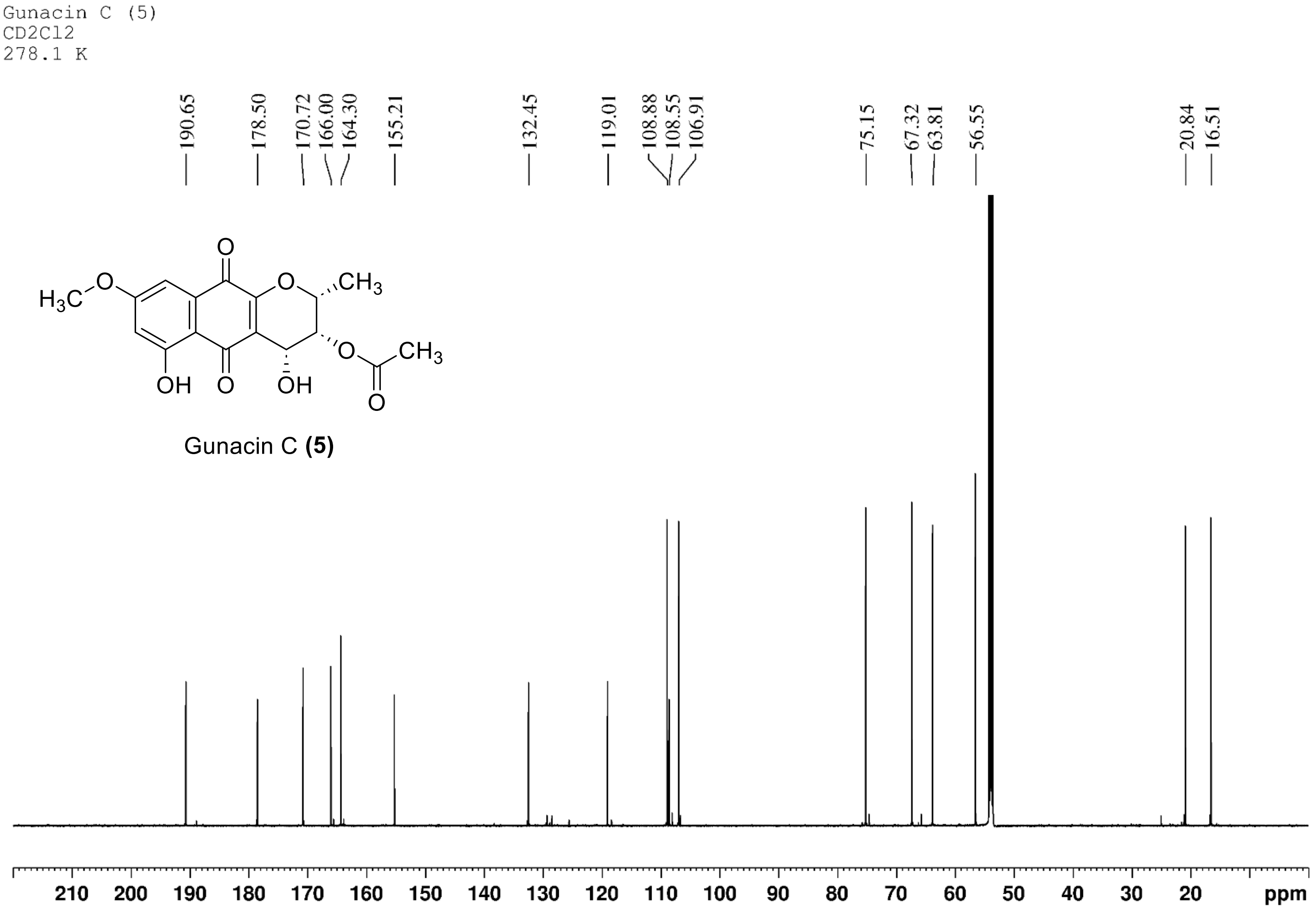

Figure S37 ^{13}C NMR spectrum of **5** (CD_2Cl_2, 278.2 K, 176.05 MHz)

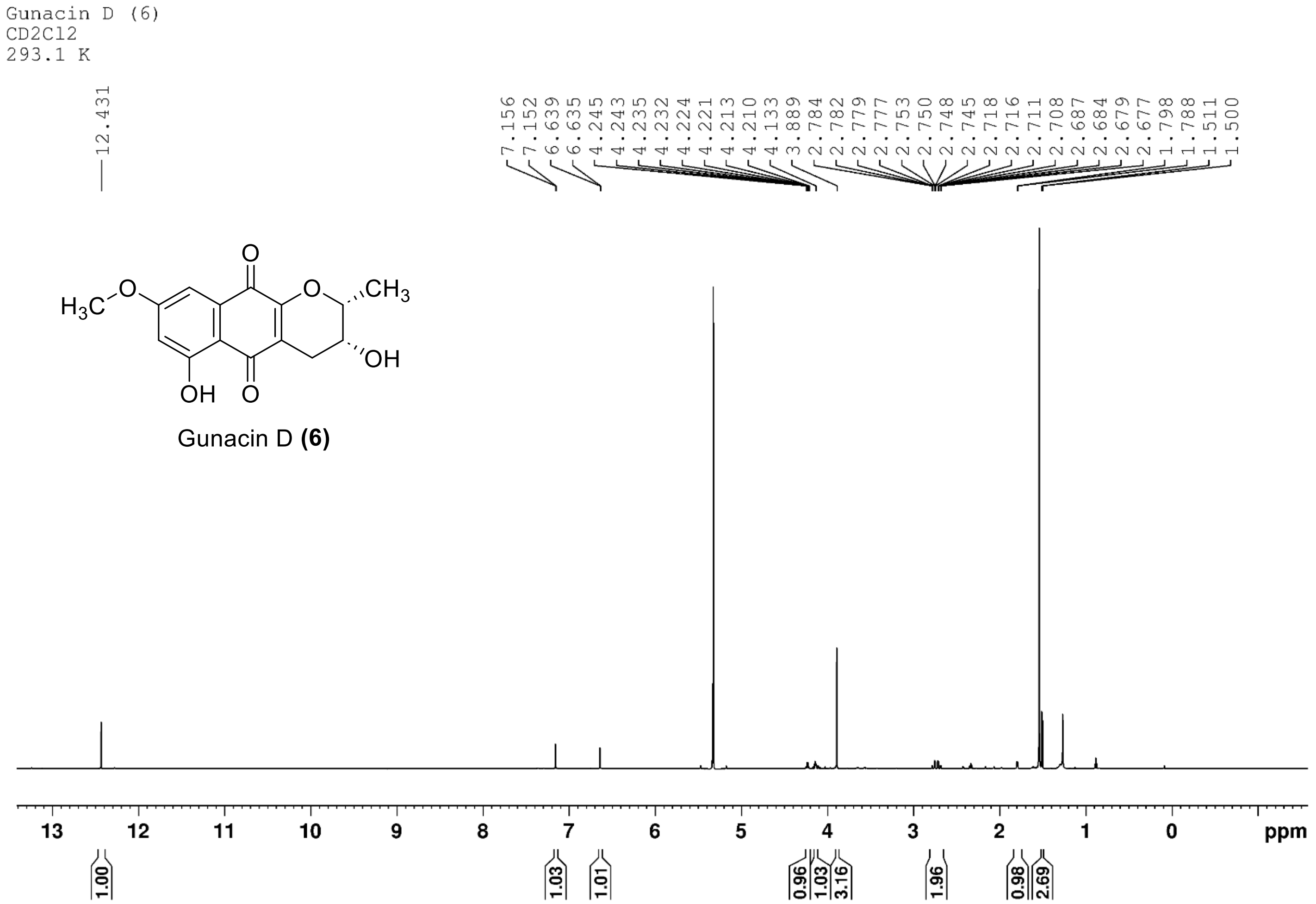

Figure S38 ^{1}H NMR spectrum of **6** (CD_2Cl_2, 293.2 K, 600.23 MHz)

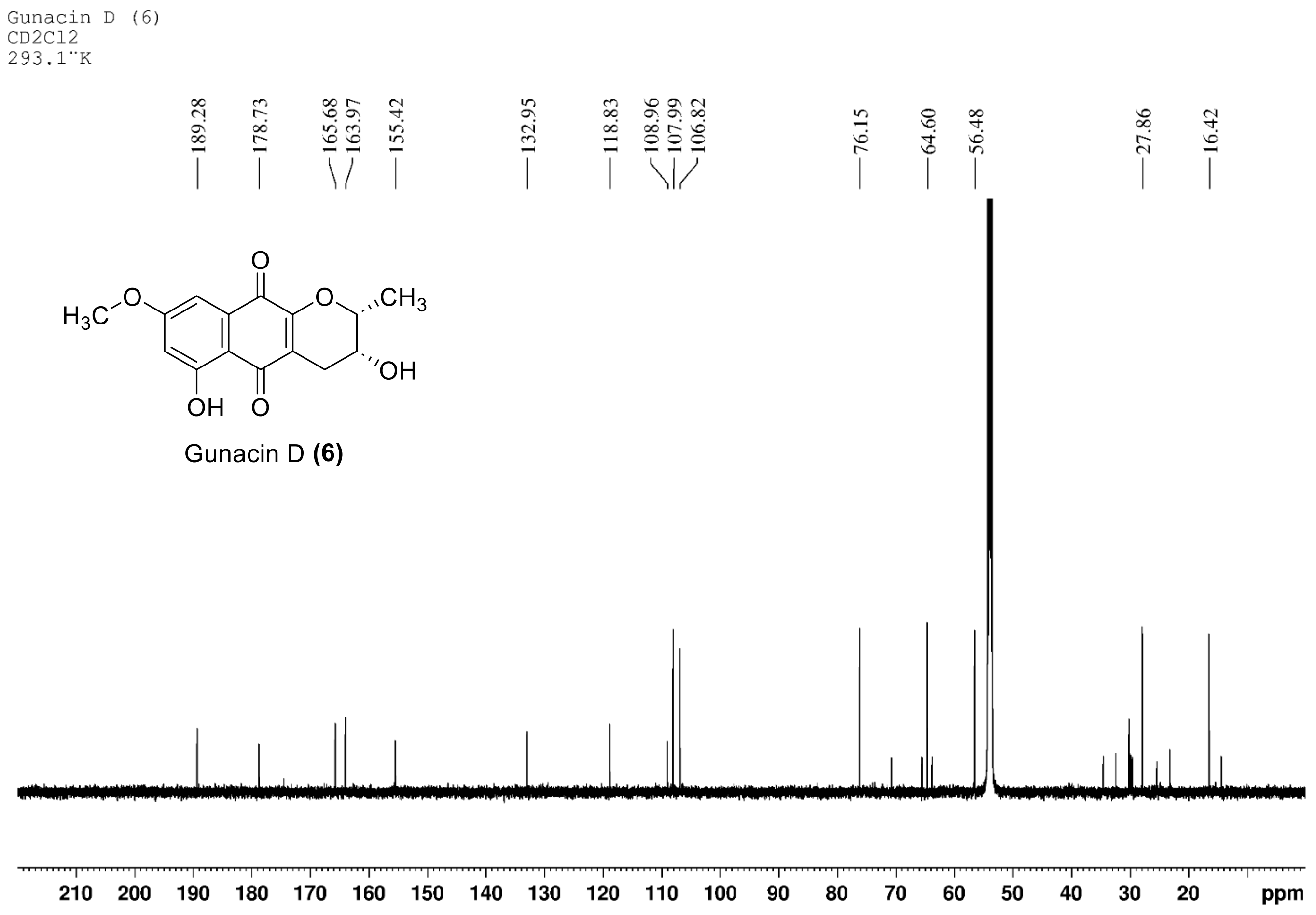

Figure S39 ^{13}C NMR spectrum of **6** (CD_2Cl_2, 293.2 K, 150.93 MHz)

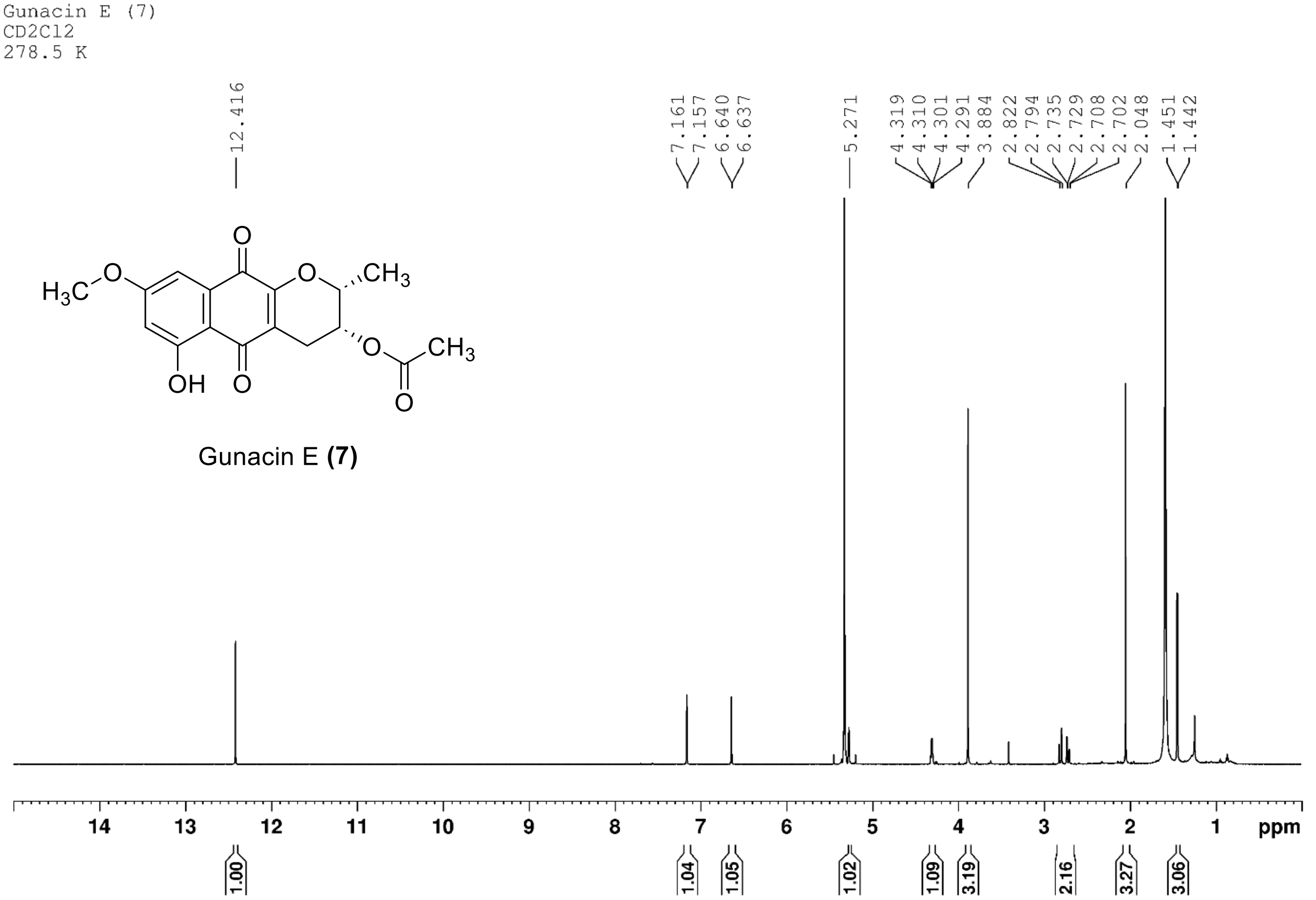

Figure S40 ^{1}H NMR spectrum of **7** (CD_2Cl_2, 278.2 K, 700.13 MHz)

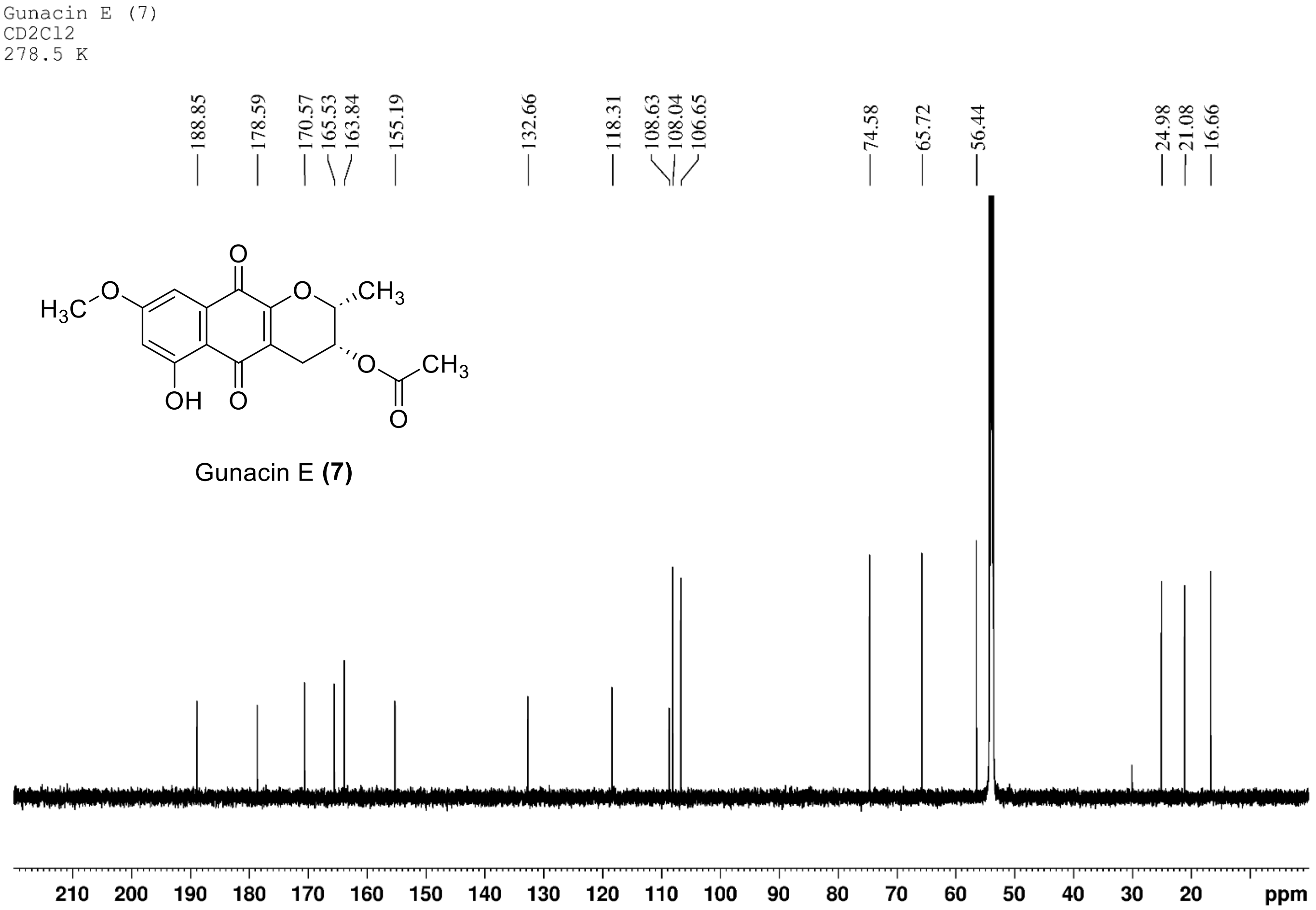

Figure S41 ^{13}C NMR spectrum of **7** (CD_2Cl_2, 278.2 K, 176.05 MHz)

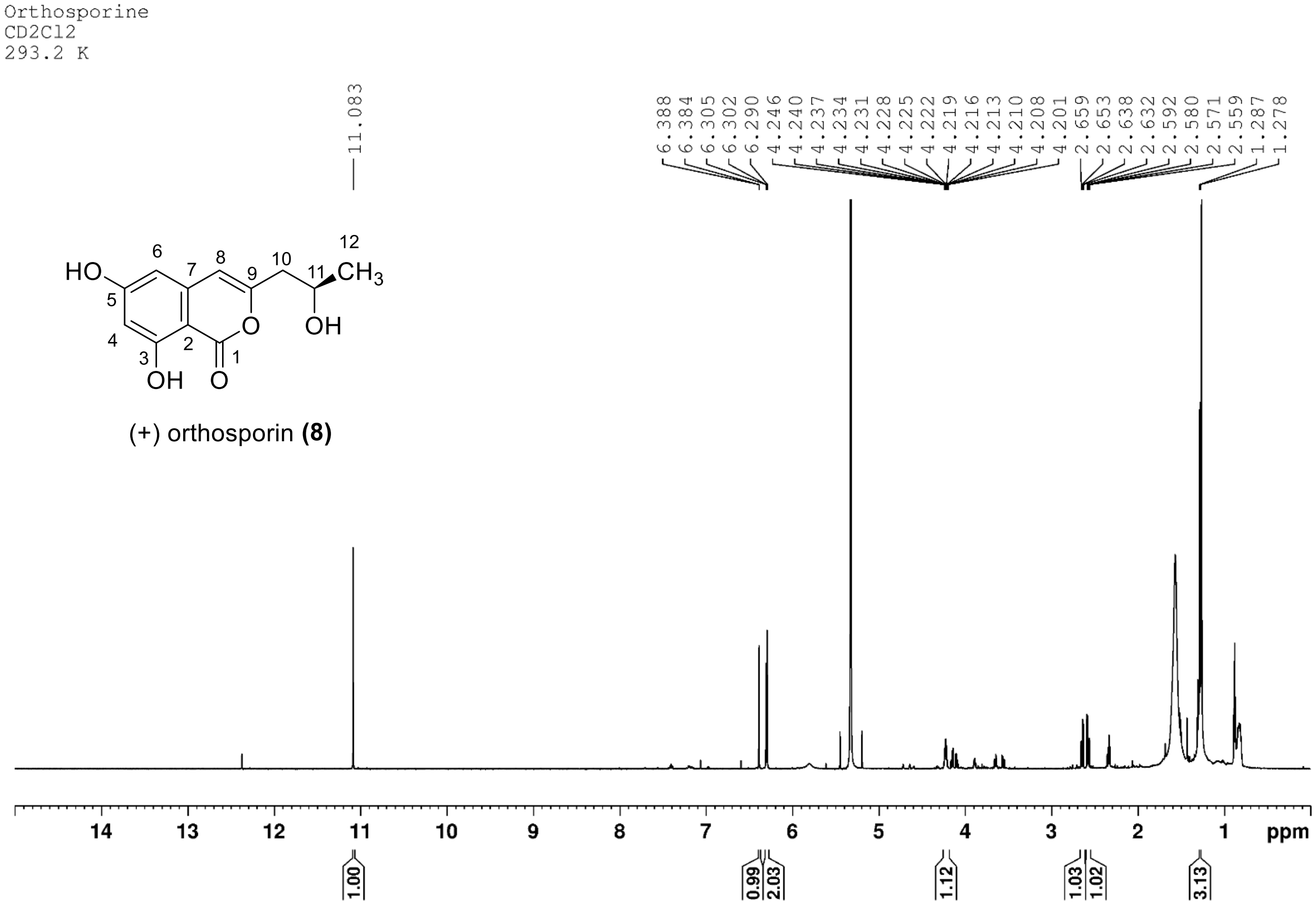

Figure S42 ^{1}H NMR spectrum of **8** (CD_2Cl_2, 293.2 K, 700.13 MHz)

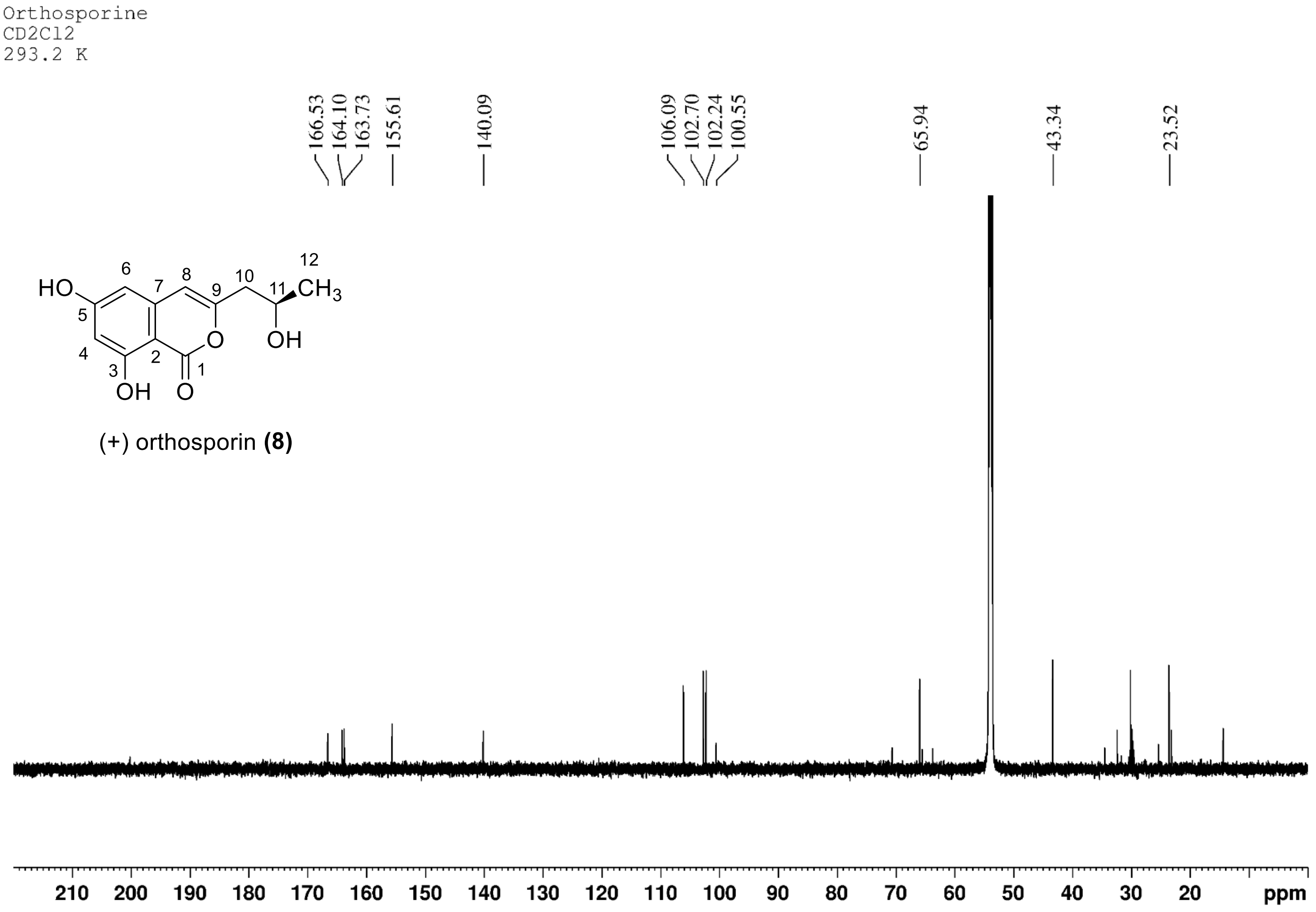

Figure S43 ^{13}C NMR spectrum of **8** (CD_2Cl_2, 293.2 K, 176.05 MHz)

Biological activity data of compounds XYZ

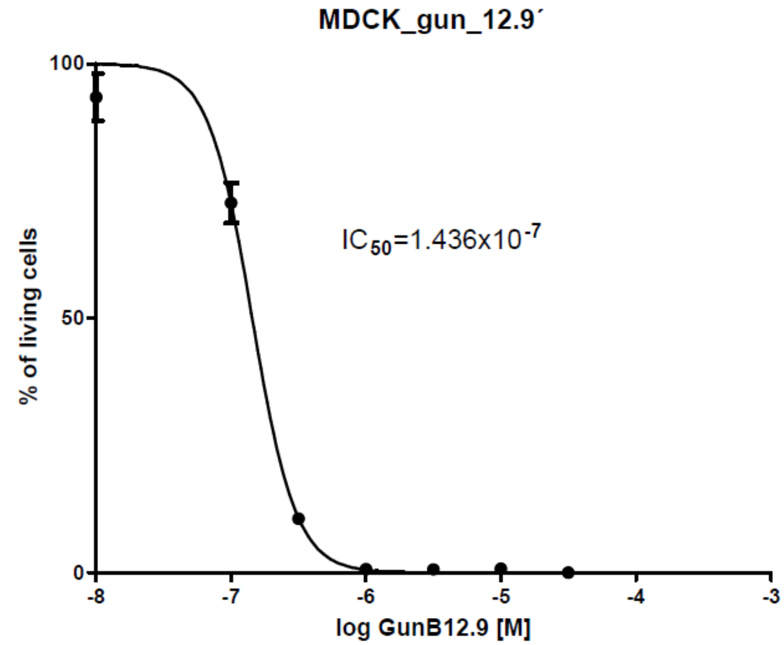

Figure S44 Cytotoxic activity of **5** against MDCK cell line

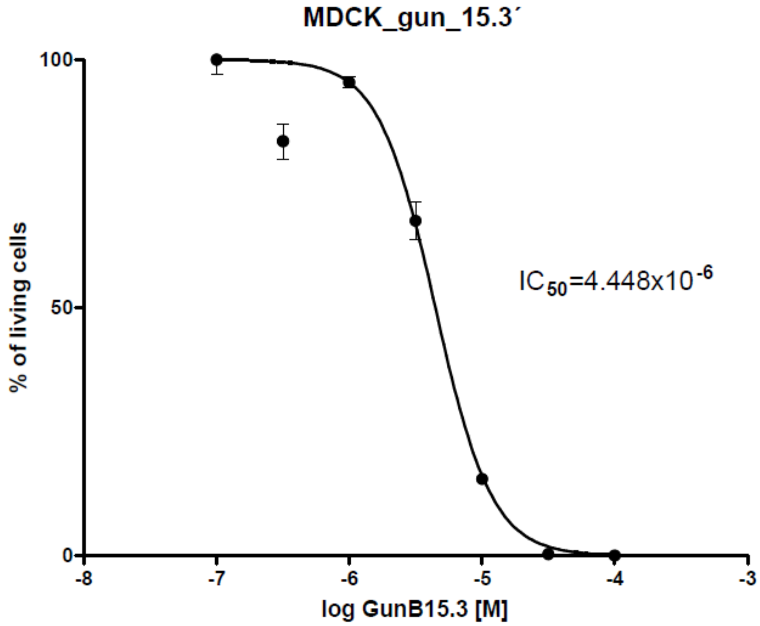

Figure S45 Cytotoxic activity of **7** against MDCK cell line

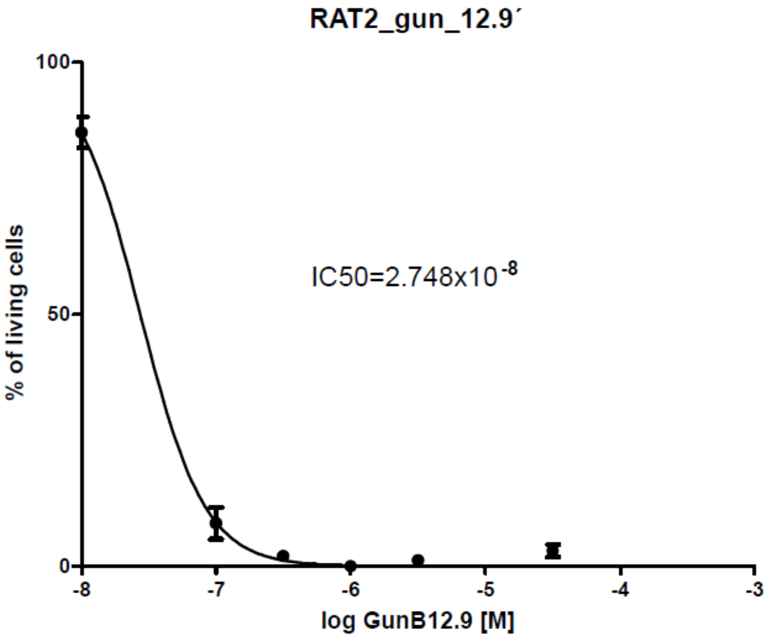

Figure S46 Cytotoxic activity of **5** against RAT2 cell line

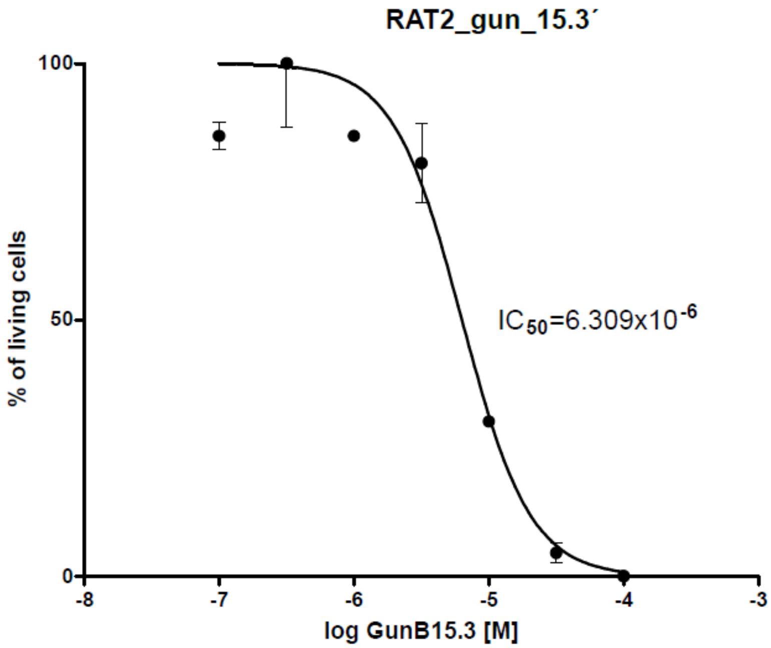

Figure S47 Cytotoxic activity of **7** against RAT2 cell line

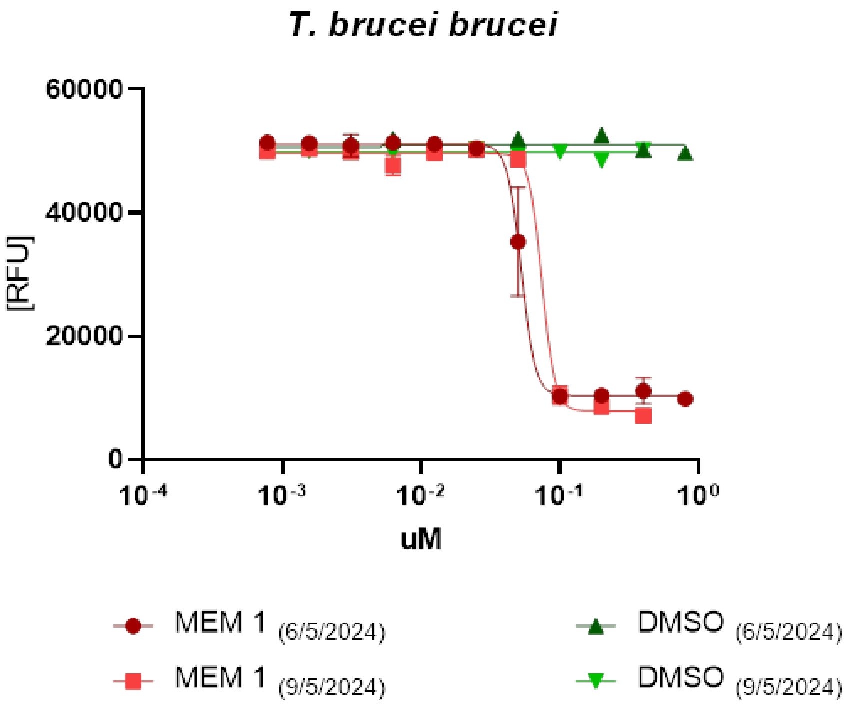

Figure S48 Antitrypanosomal activity of **1** against *Trypanosoma brucei brucei*

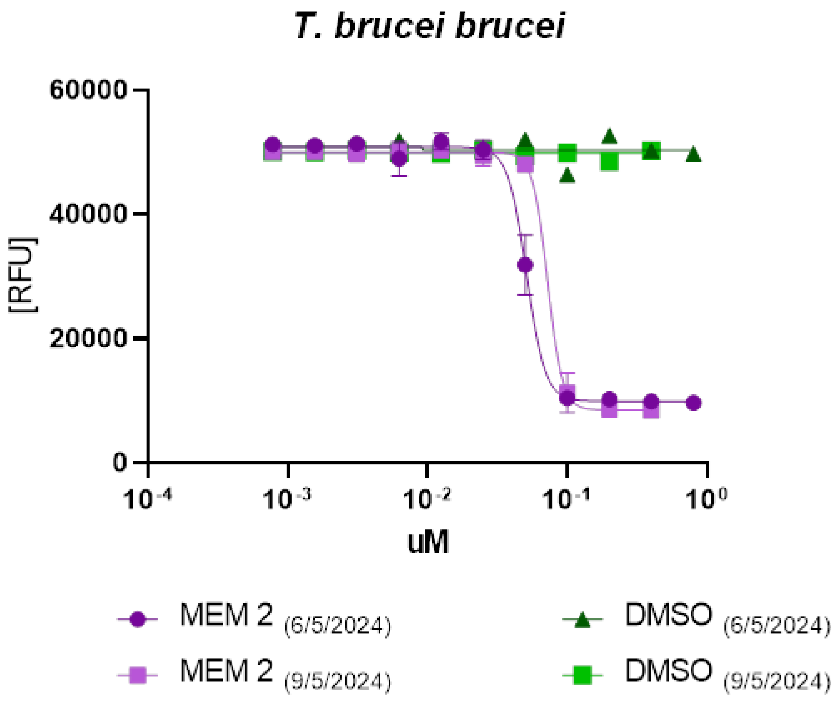

Figure S49 Antitrypanosomal activity of **5** against *Trypanosoma brucei brucei*

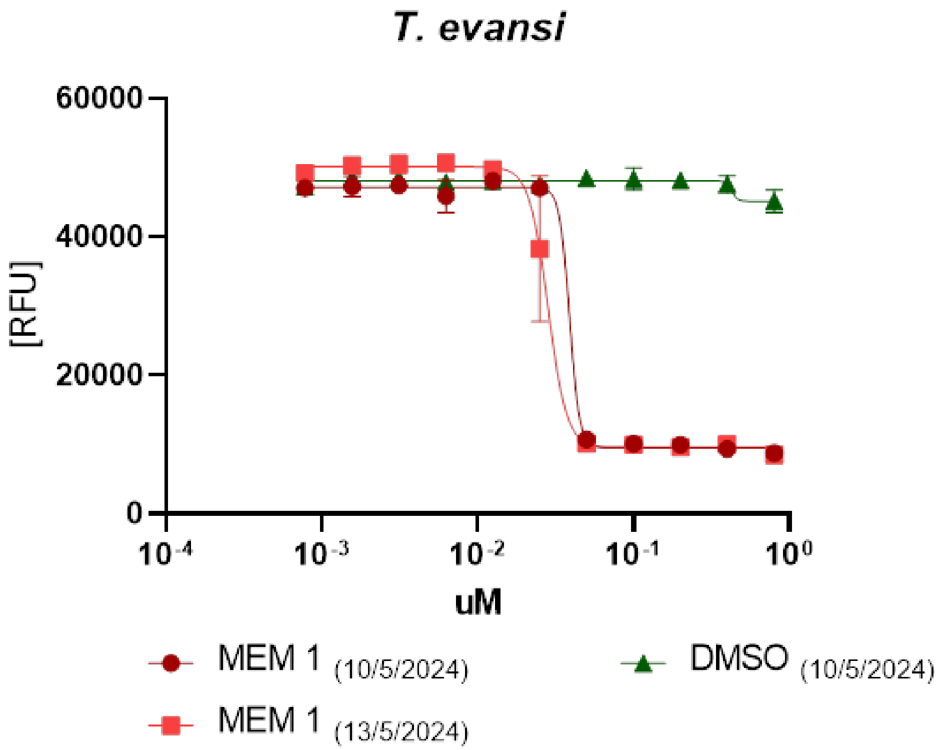

Figure S50 Antitrypanosomal activity of **1** against *Trypanosoma evansi*

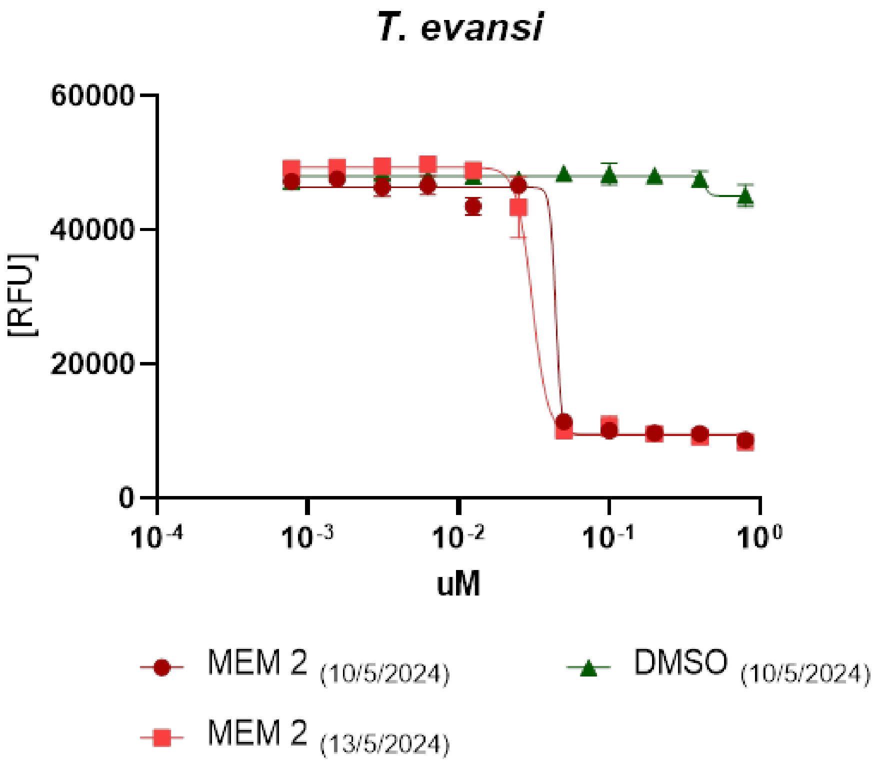

Figure S51 Antitrypanosomal activity of **5** against *Trypanosoma evansi*

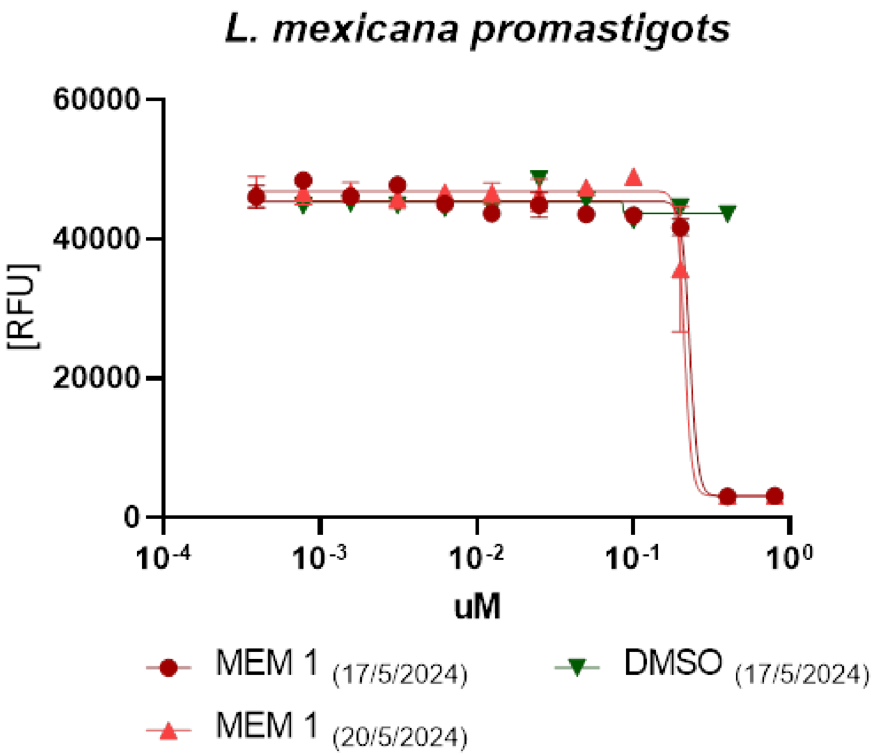

Figure S52 Antileishmanial activity of **1** against *Leishmania mexicana promastigots*

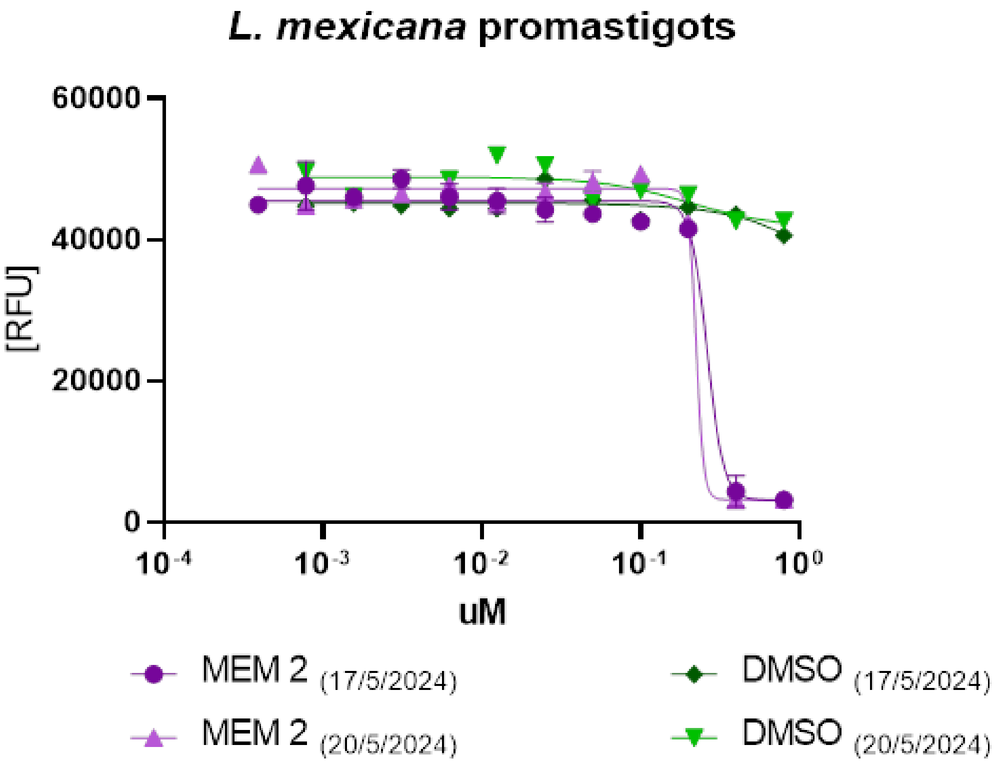

Figure S53 Antileishmanial activity of **5** against *Leishmania mexicana promastigots*

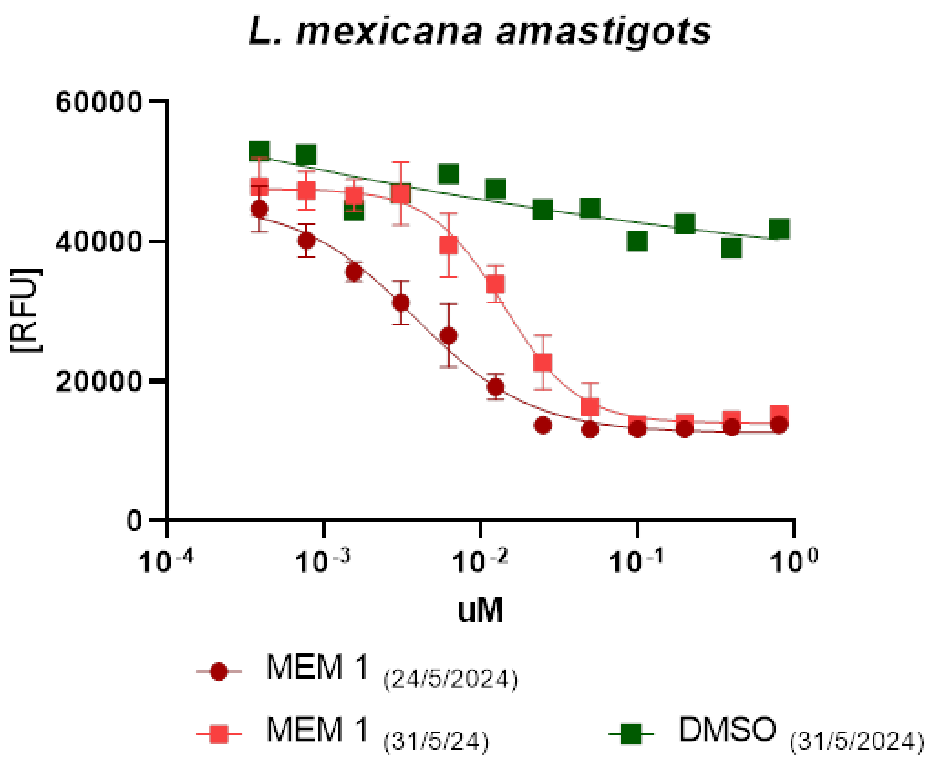

Figure S54 Antileishmanial activity of **1** against *Leishmania mexicana amastigots*

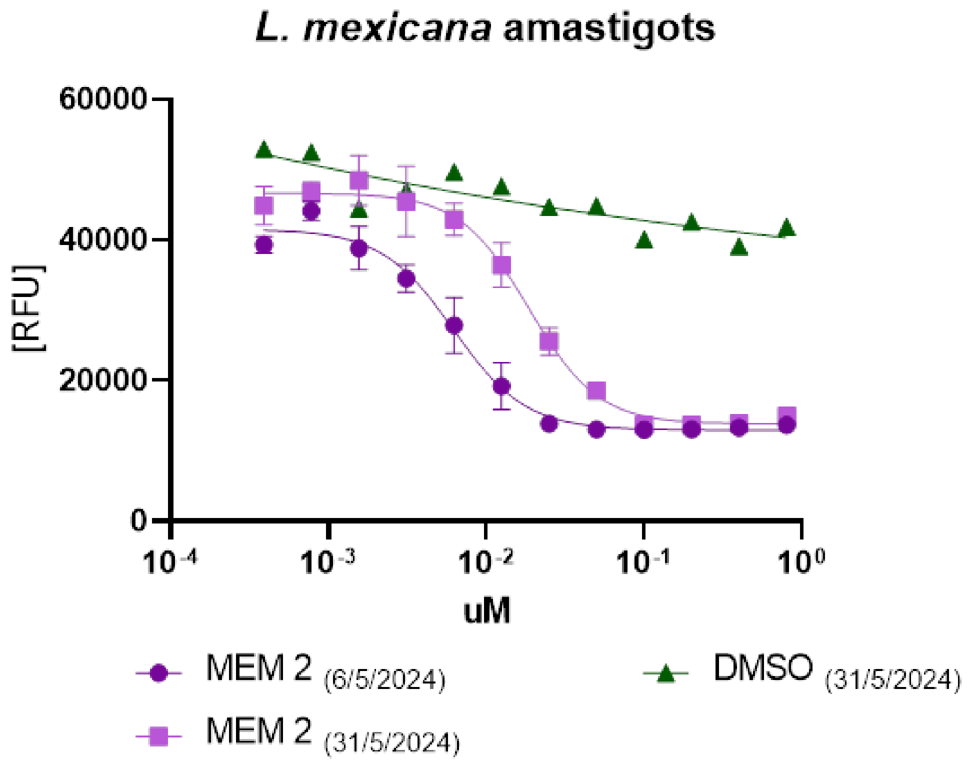

Figure S55 Antileishmanial activity of **5** against *Leishmania mexicana amastigots*

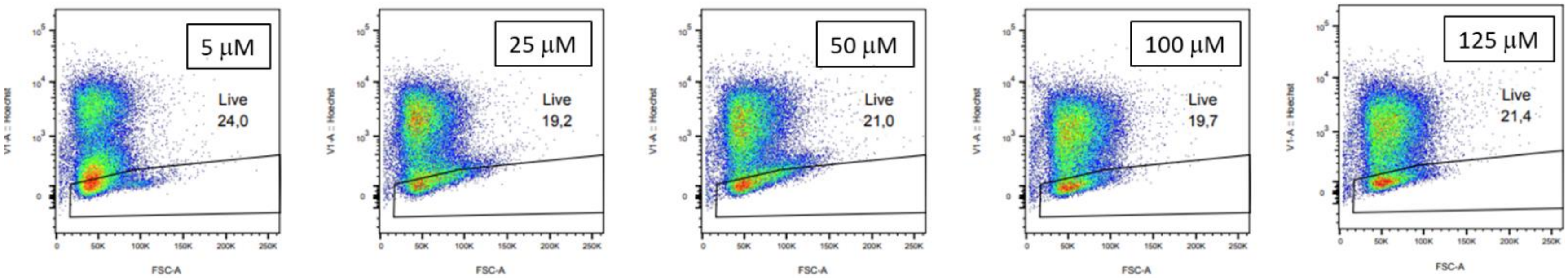

Figure S56 Cytotoxic activity of 5 against Jurkat cell line

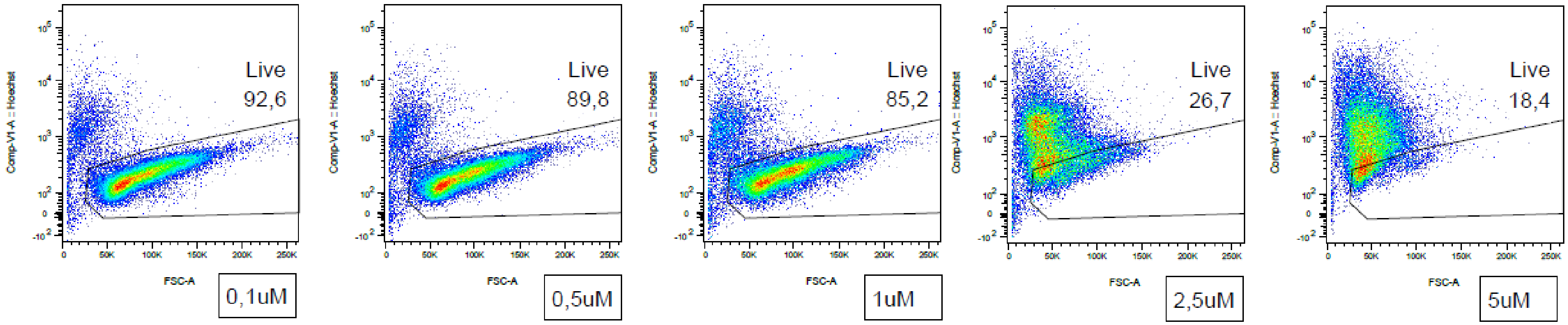

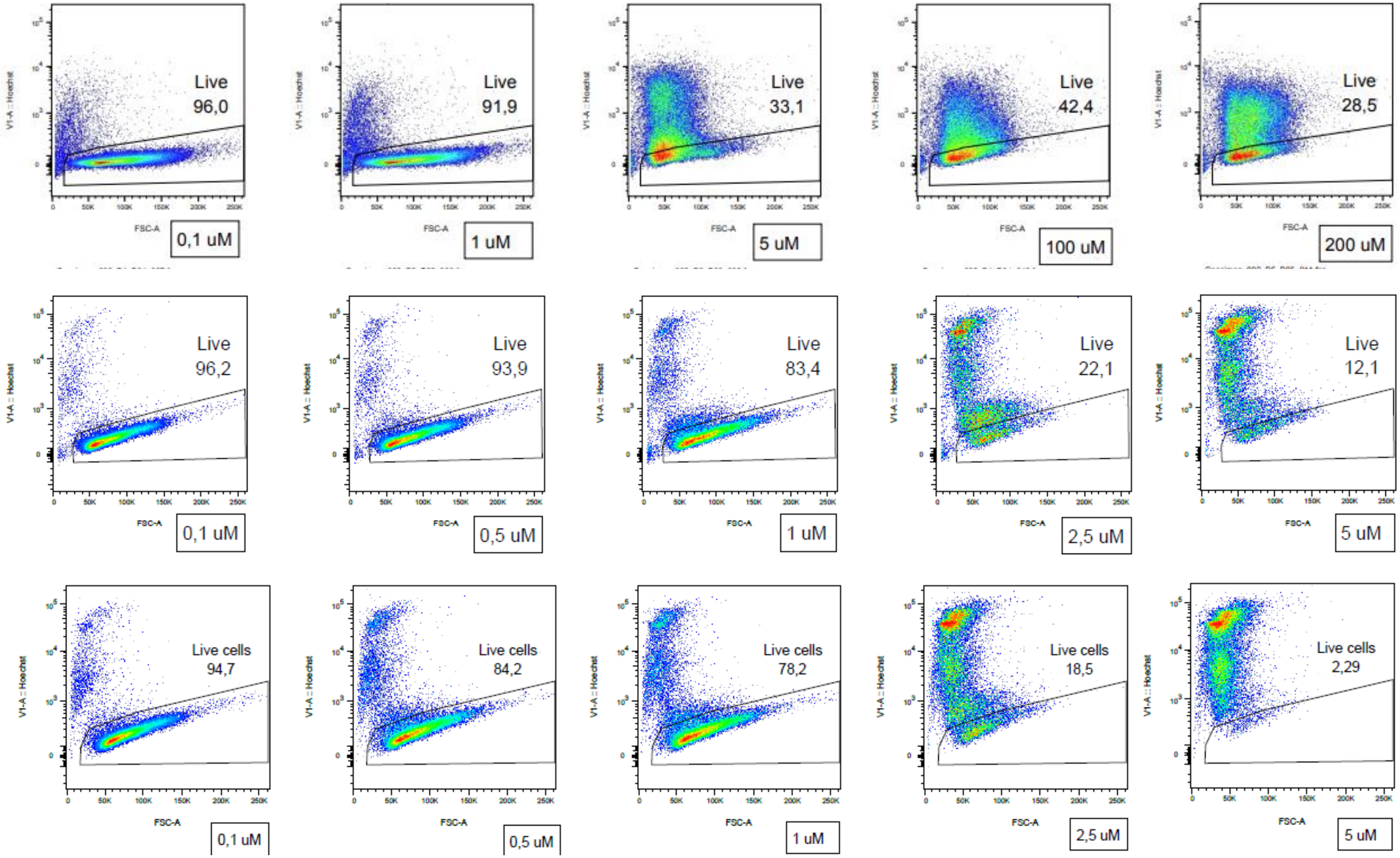

Figure S57 Cytotoxic activity of **5** against Jurkat cell line

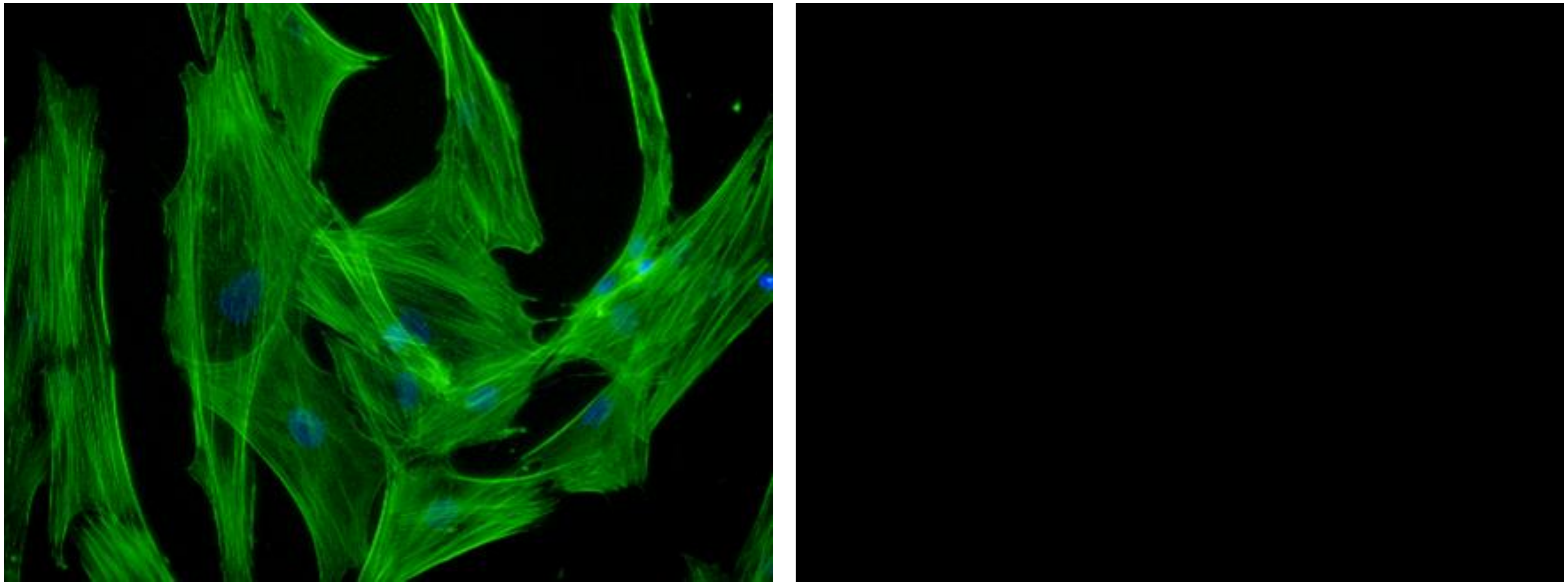

Figure S58 Effect on fibroblasts morphology of **5** at 5μM. Mitochondria are visualized, the actin cytoskeleton with Phalloidin (green), and nuclei with DAPI (blue).

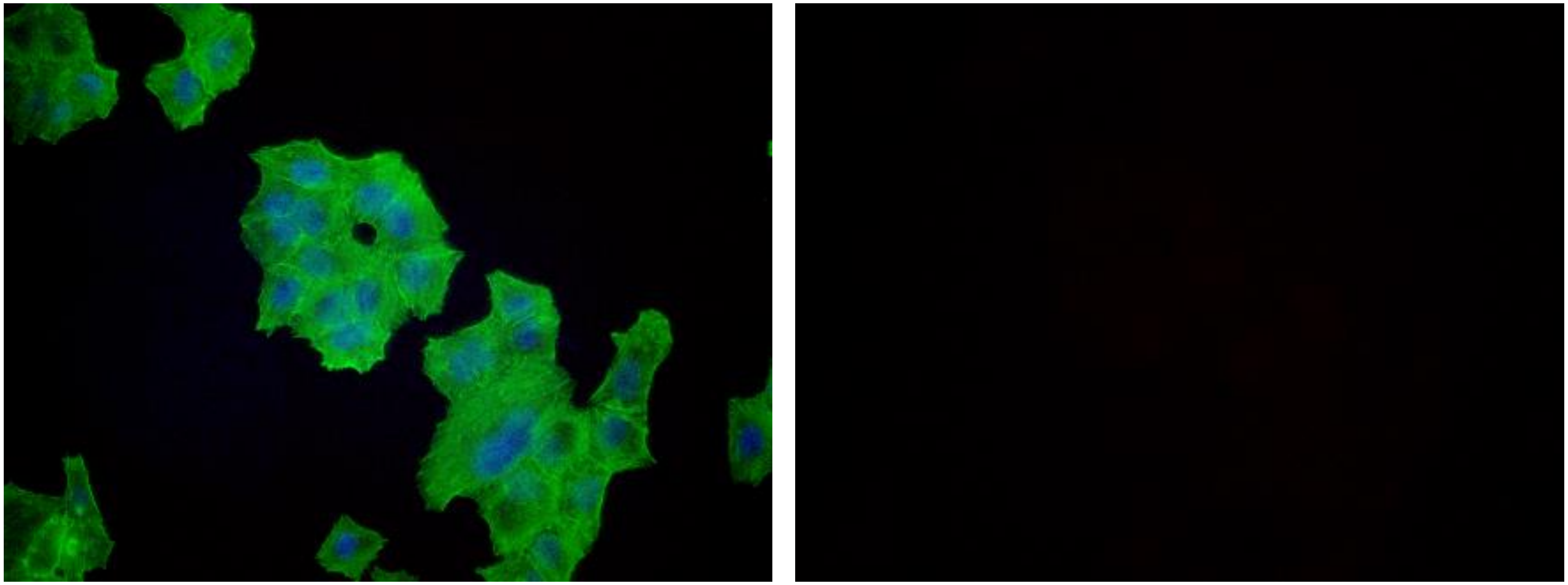

Figure S59 Effect on HeLa morphology of **5** at 125μM. Mitochondria are visualized using the actin cytoskeleton with Phalloidin (green), and nuclei with DAPI (blue).

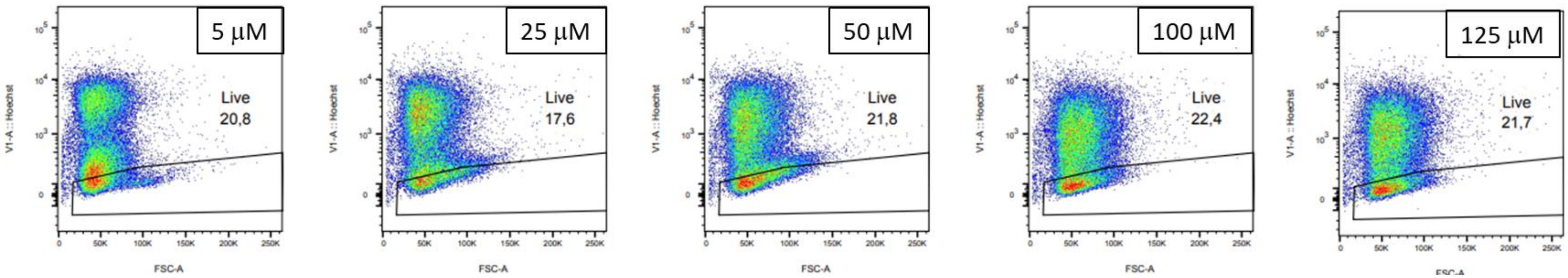

Figure S60 Cytotoxic activity of **1** against Jurkat cell line

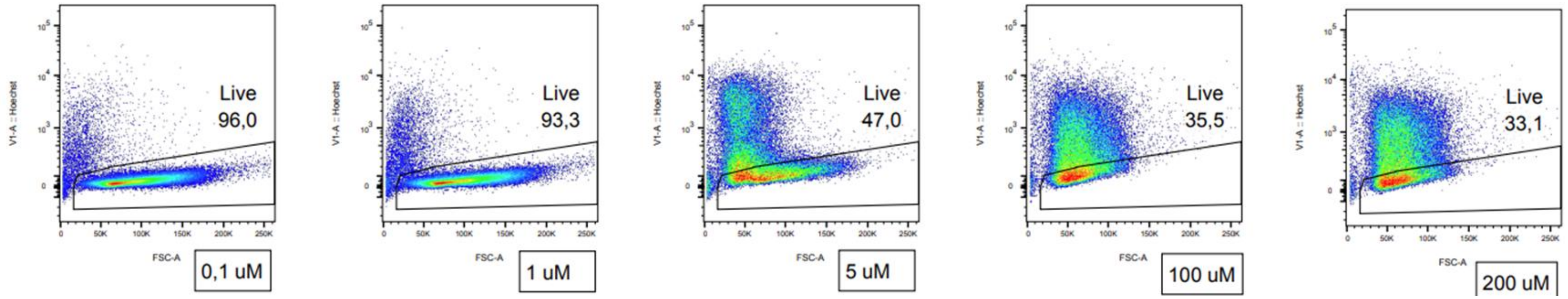

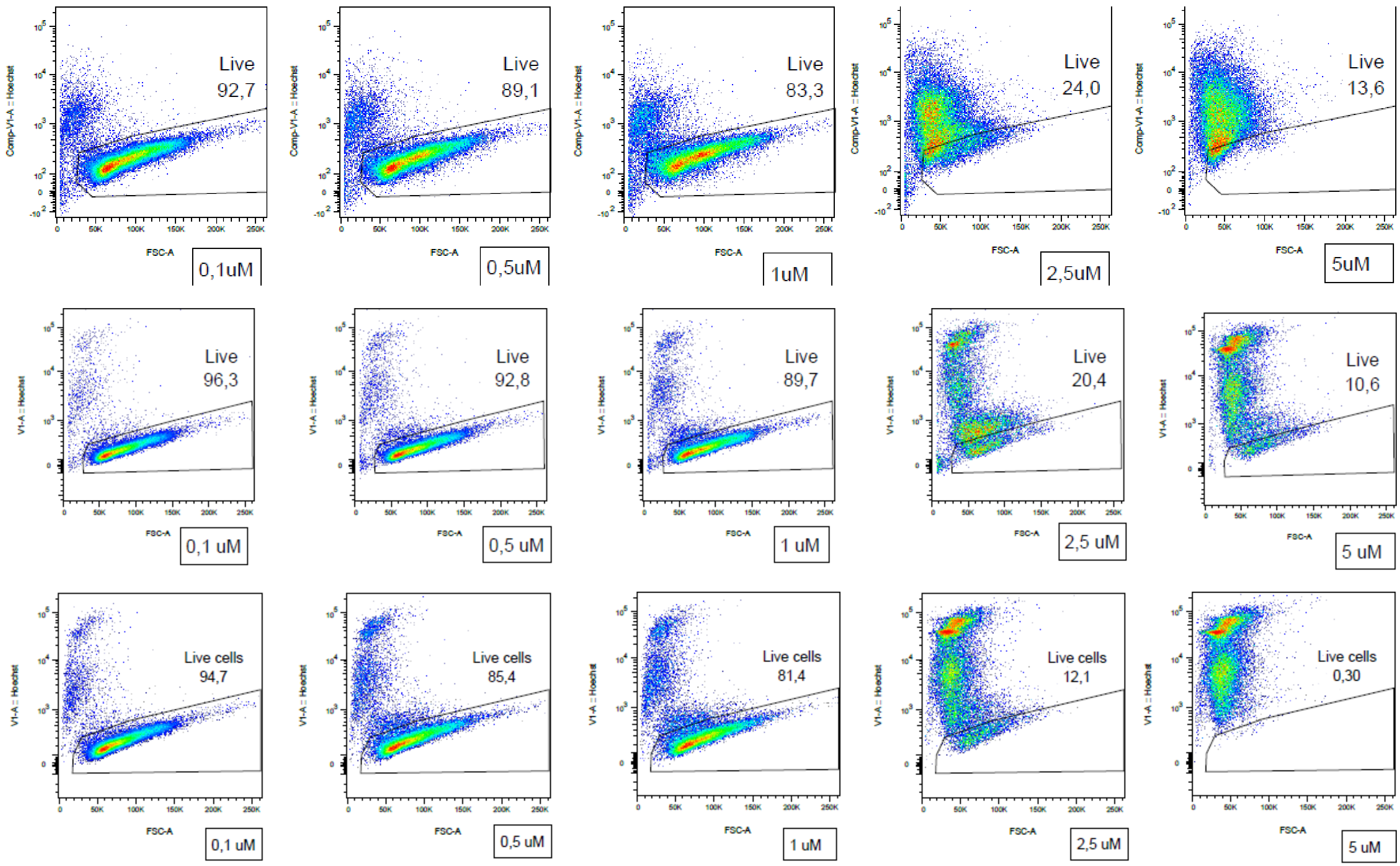

Figure 61 Cytotoxic activity of **1** against Jurkat cell line

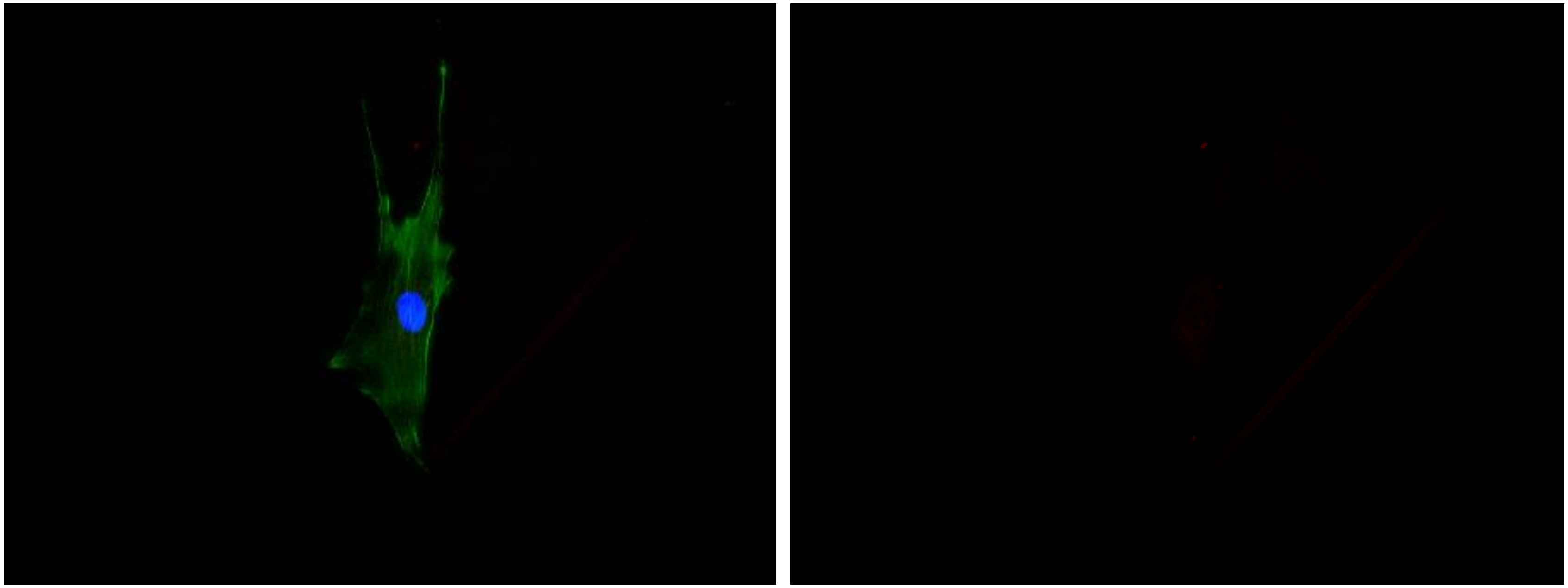

Figure S62 Effect on fibroblasts morphology of **1** at 5μM. Mitochondria are visualized using the actin cytoskeleton with Phalloidin (green), and nuclei with DAPI (blue).

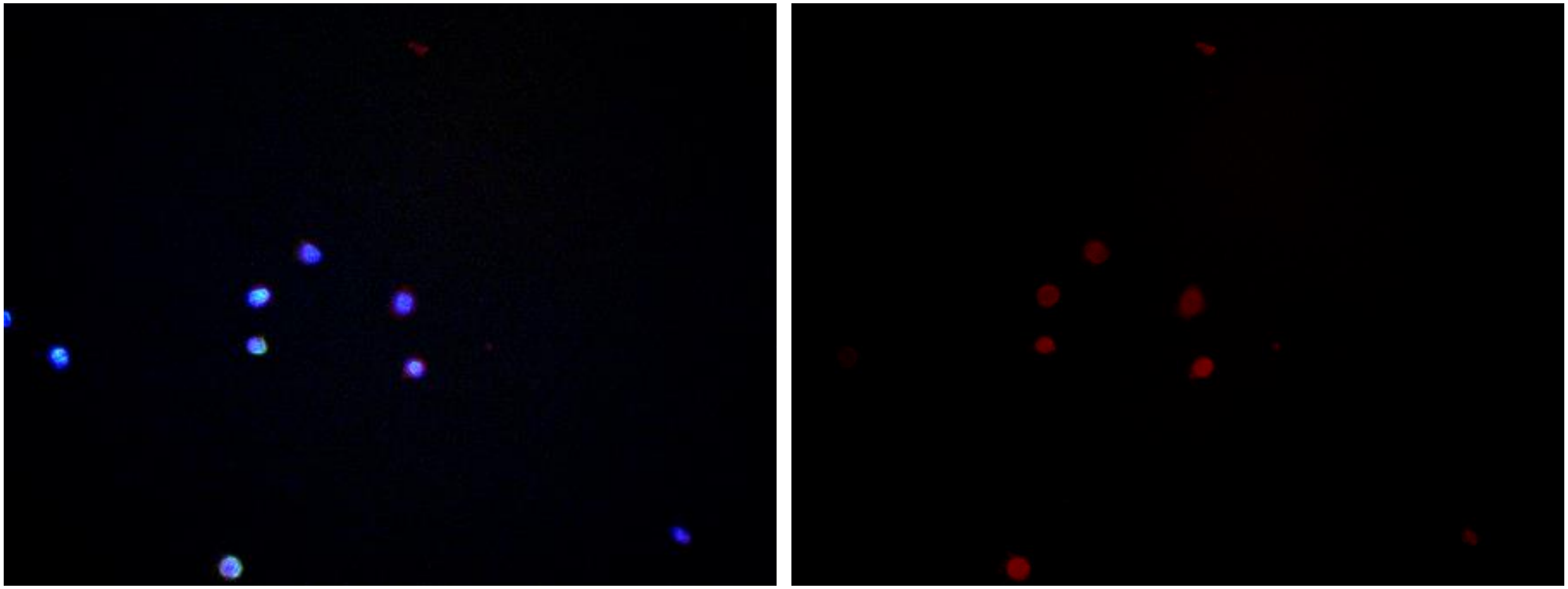

Figure S63 Effect on HeLa morphology of **1** at 125μM. Mitochondria are visualized using MitoTracker Red CMXRos (red), the actin cytoskeleton with Phalloidin (green), and nuclei with DAPI (blue).

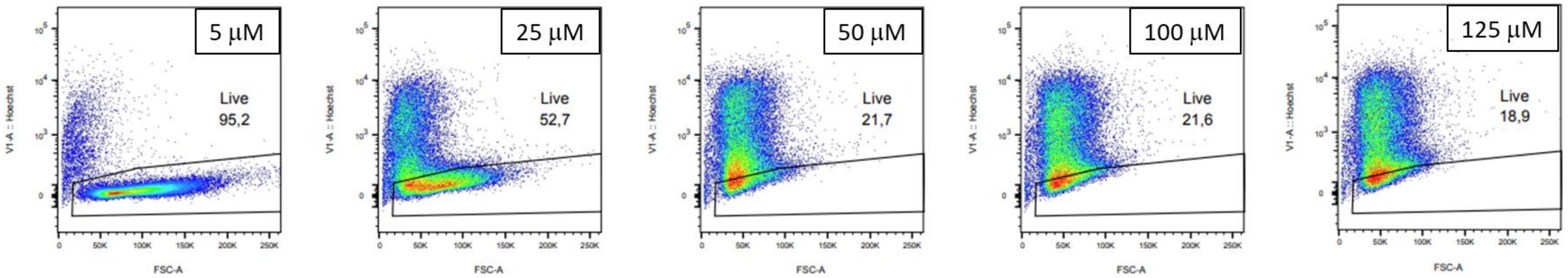

Figure S64 Cytotoxic activity of **3** against Jurkat cell line

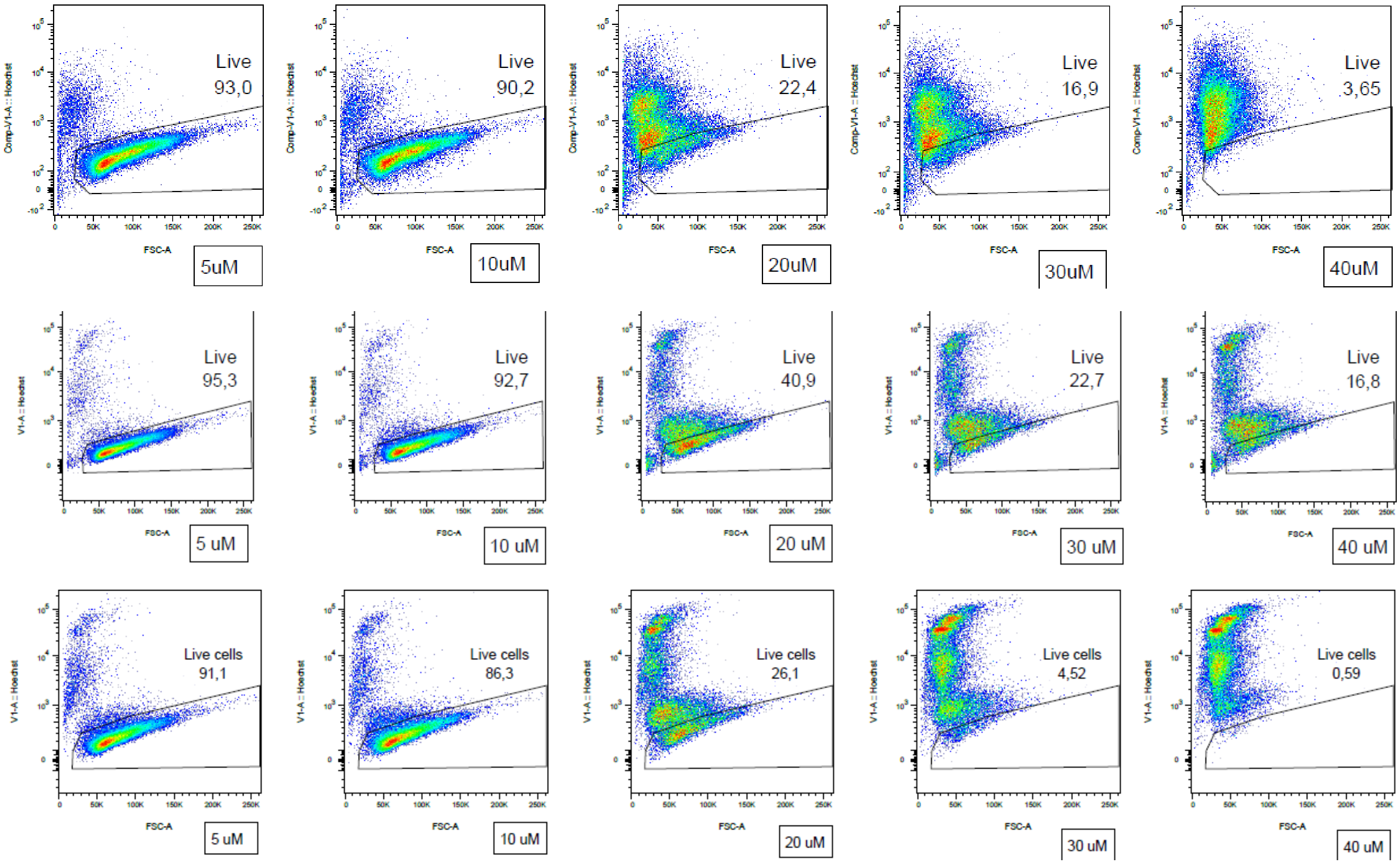

Figure S65 Cytotoxic activity of **3** against Jurkat cell line

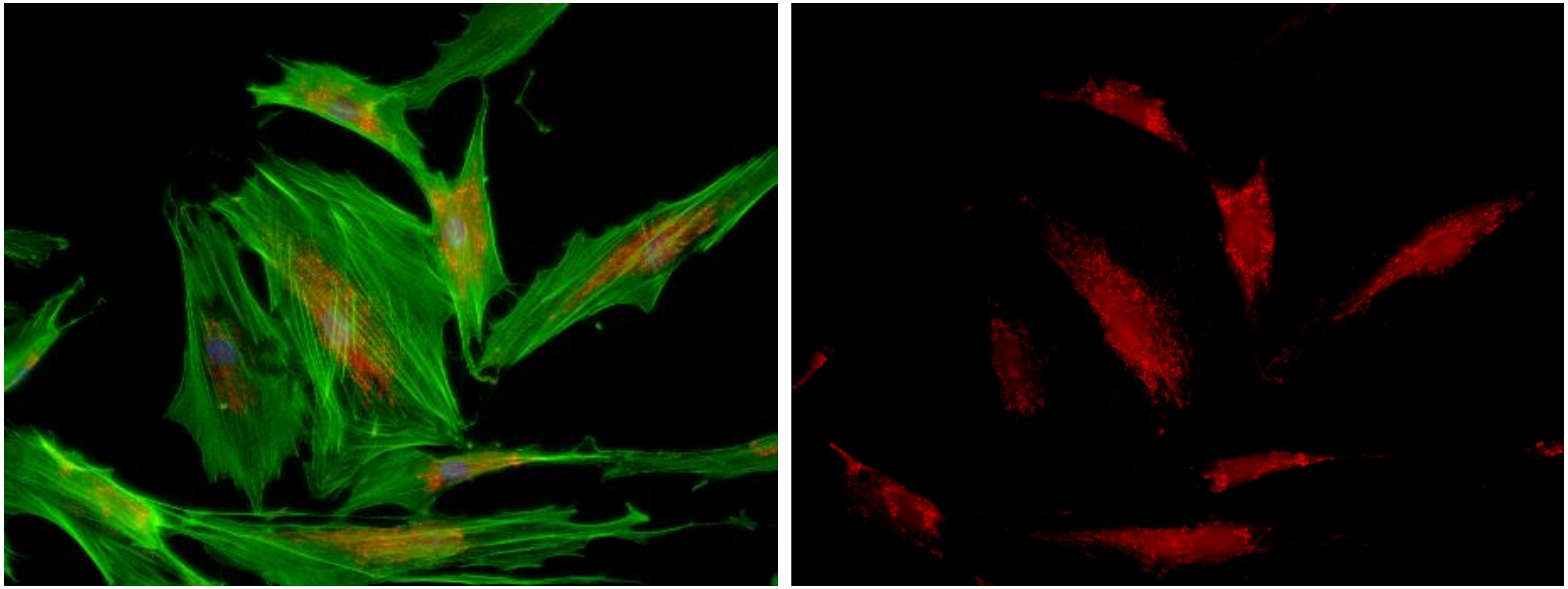

Figure S66 Effect on fibroblasts morphology of **3** at 125μM. Mitochondria are visualized using MitoTracker Red CMXRos (red), the actin cytoskeleton with Phalloidin (green), and nuclei with DAPI (blue).

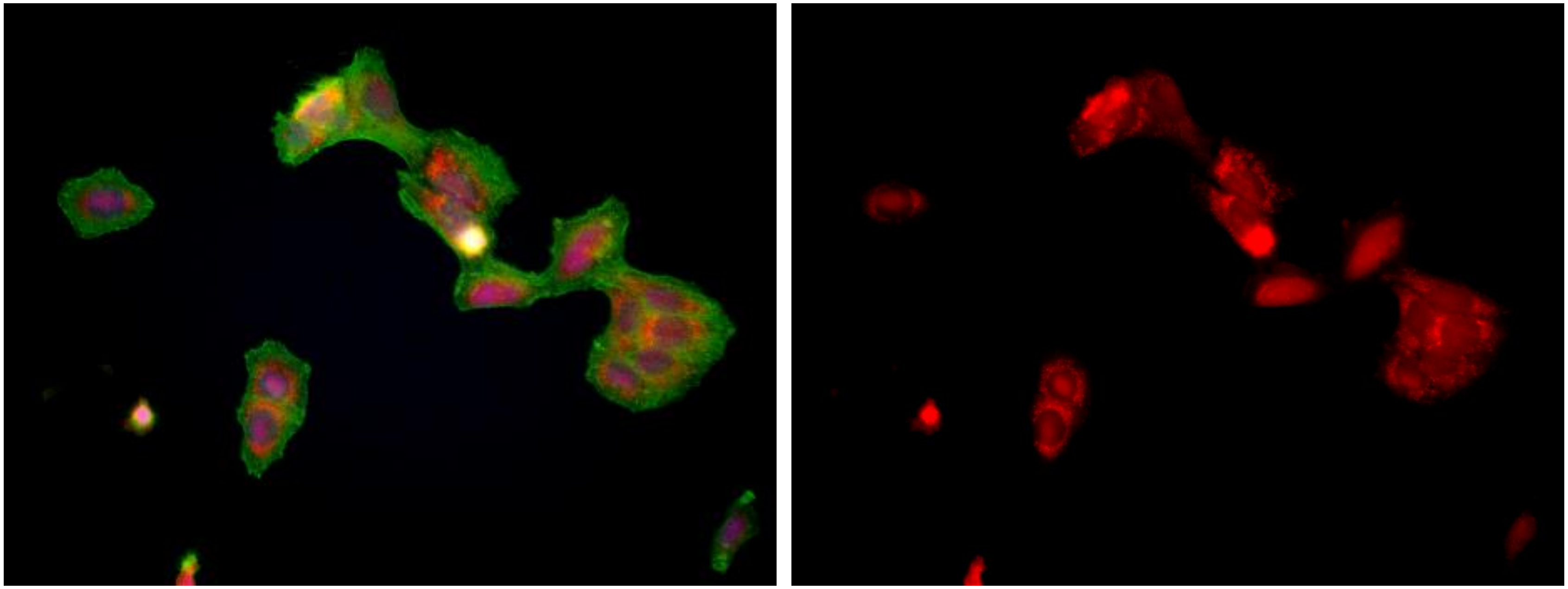

Figure S67 Effect on HeLa cells morphology of **3** at 25μM. Mitochondria are visualized using MitoTracker Red CMXRos (red), the actin cytoskeleton with Phalloidin (green), and nuclei with DAPI (blue).

D15.3

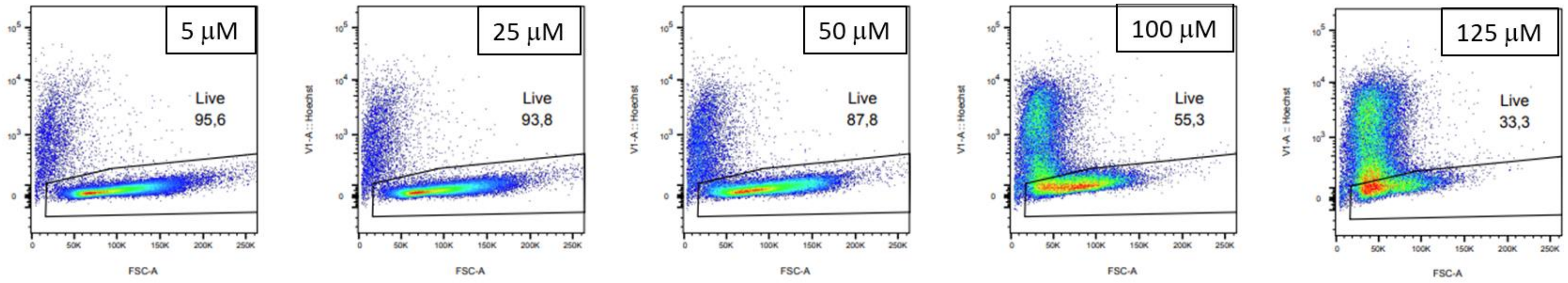

Figure 68 Cytotoxic activity of **7** against Jurkat cell line

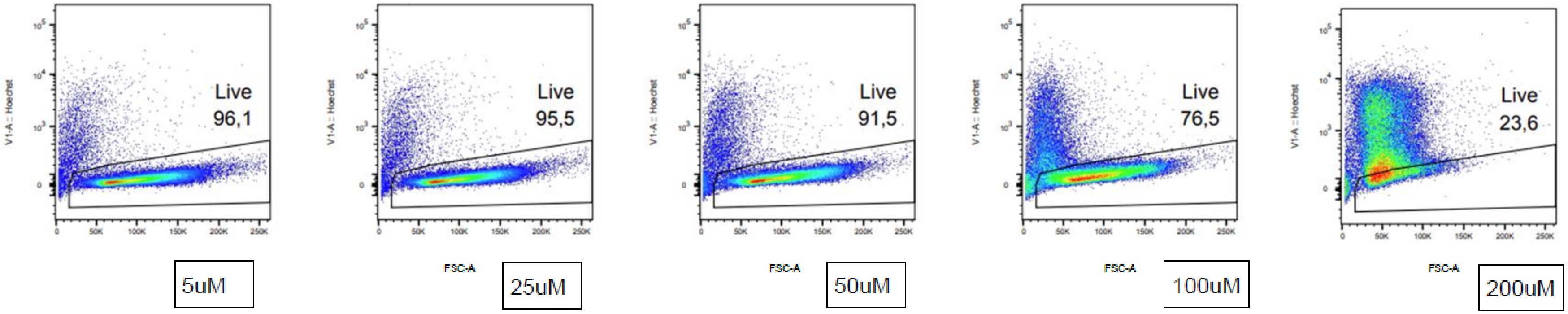

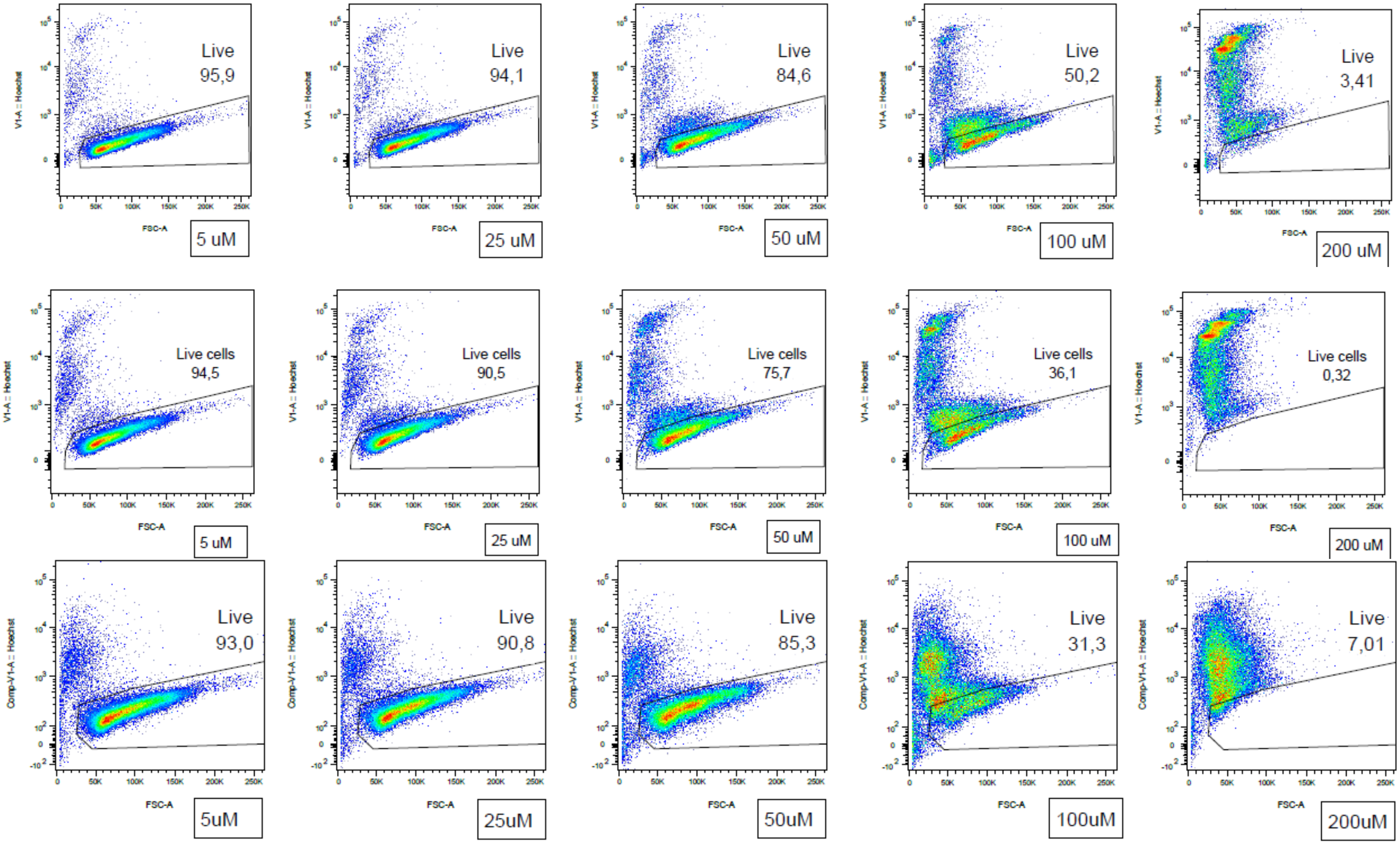

Figure 69 Cytotoxic activity of **7** against Jurkat cell line

25 mM, 125 kompletně mrtvo

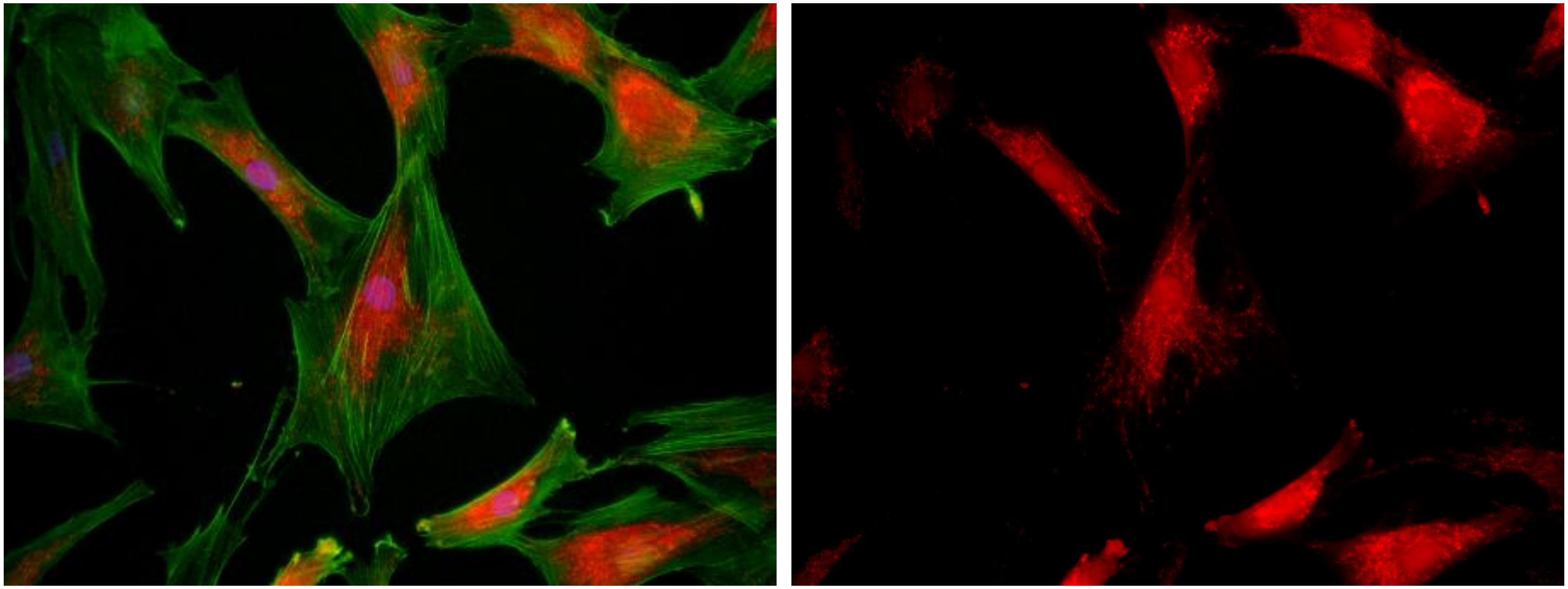

Figure S70 Effect on fibroblasts morphology of **7** at 25µM. Mitochondria are visualized using MitoTracker Red CMXRos (red), the actin cytoskeleton with Phalloidin (green), and nuclei with DAPI (blue).

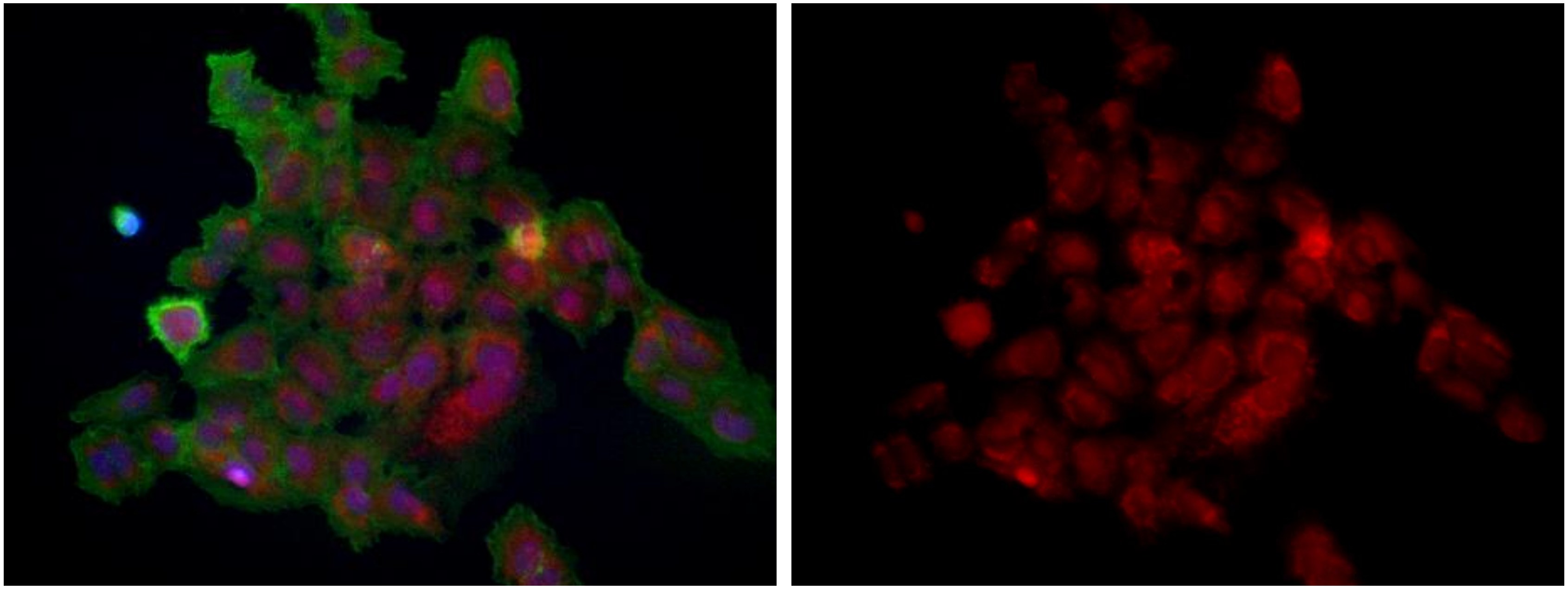

Figure S71 Effect on HeLa cells morphology of **3** at 125μM. Mitochondria are visualized using MitoTracker Red CMXRos (red), the actin cytoskeleton with Phalloidin (green), and nuclei with DAPI (blue).

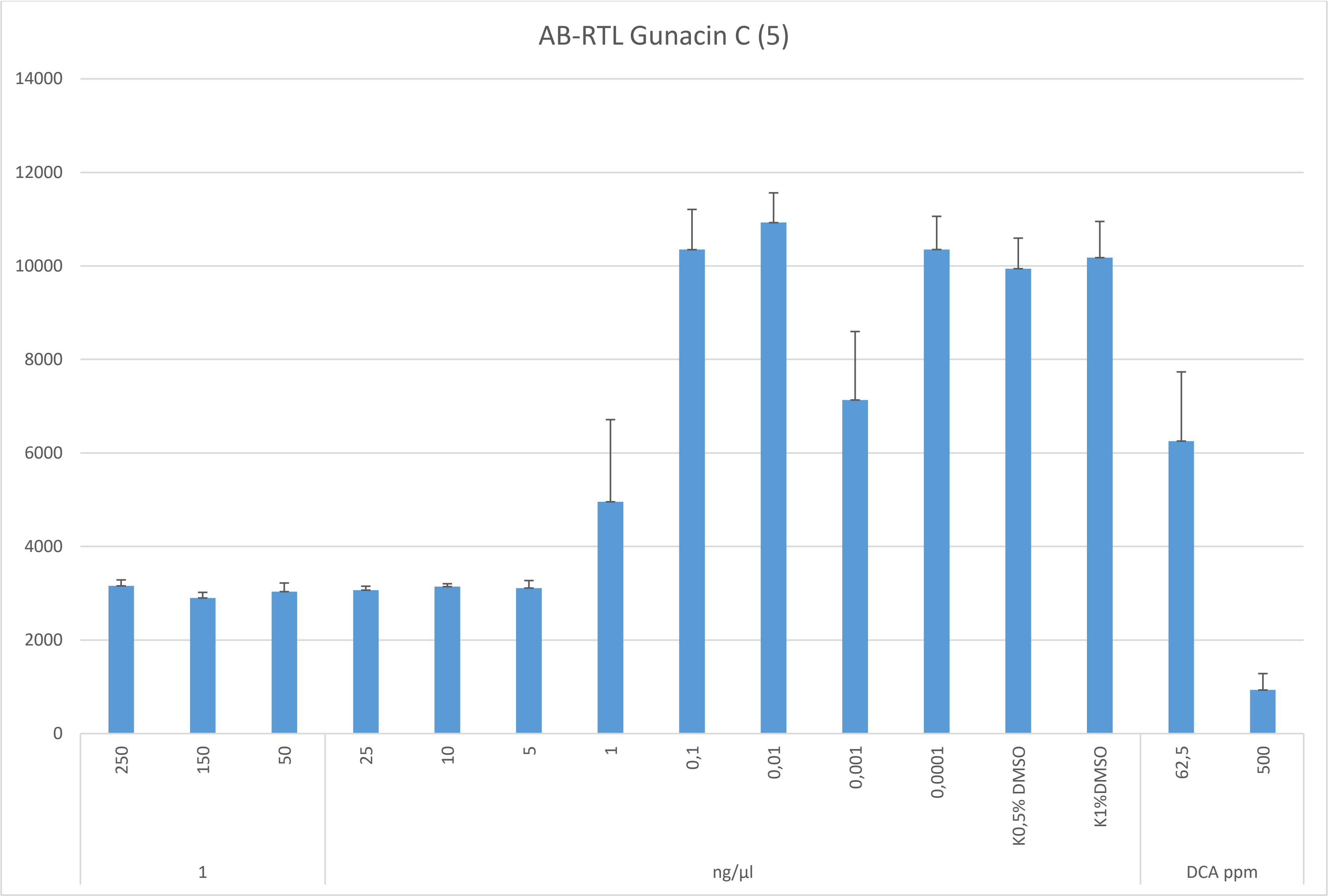

Figure S72 RTL-W1 cell line viability after treatment with **5** and almarBlue (AB) fluorescent indicator.

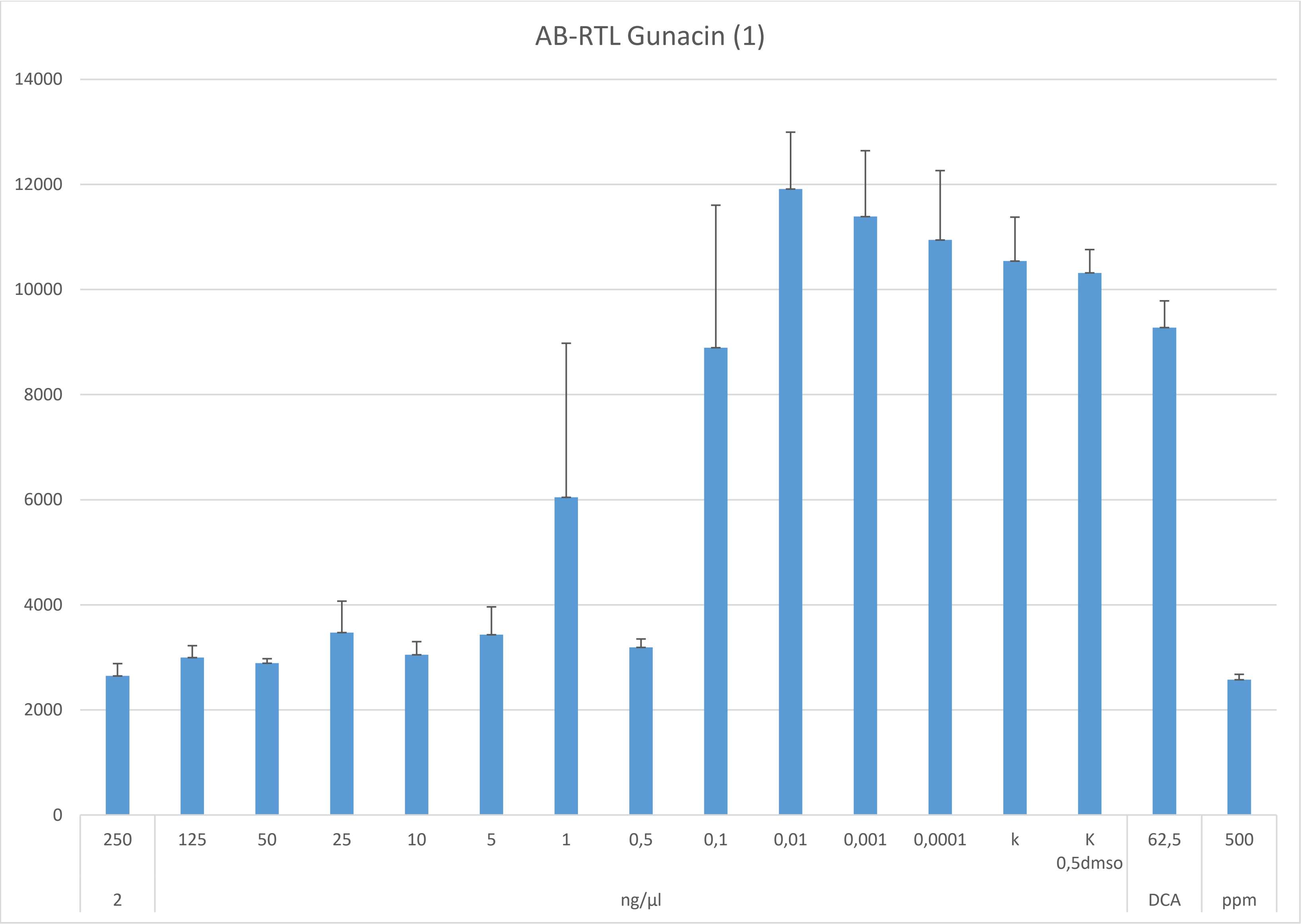

Figure S73 RTL-W1 cell line viability after treatment with **1** and almarBlue (AB) fluorescent indicator.

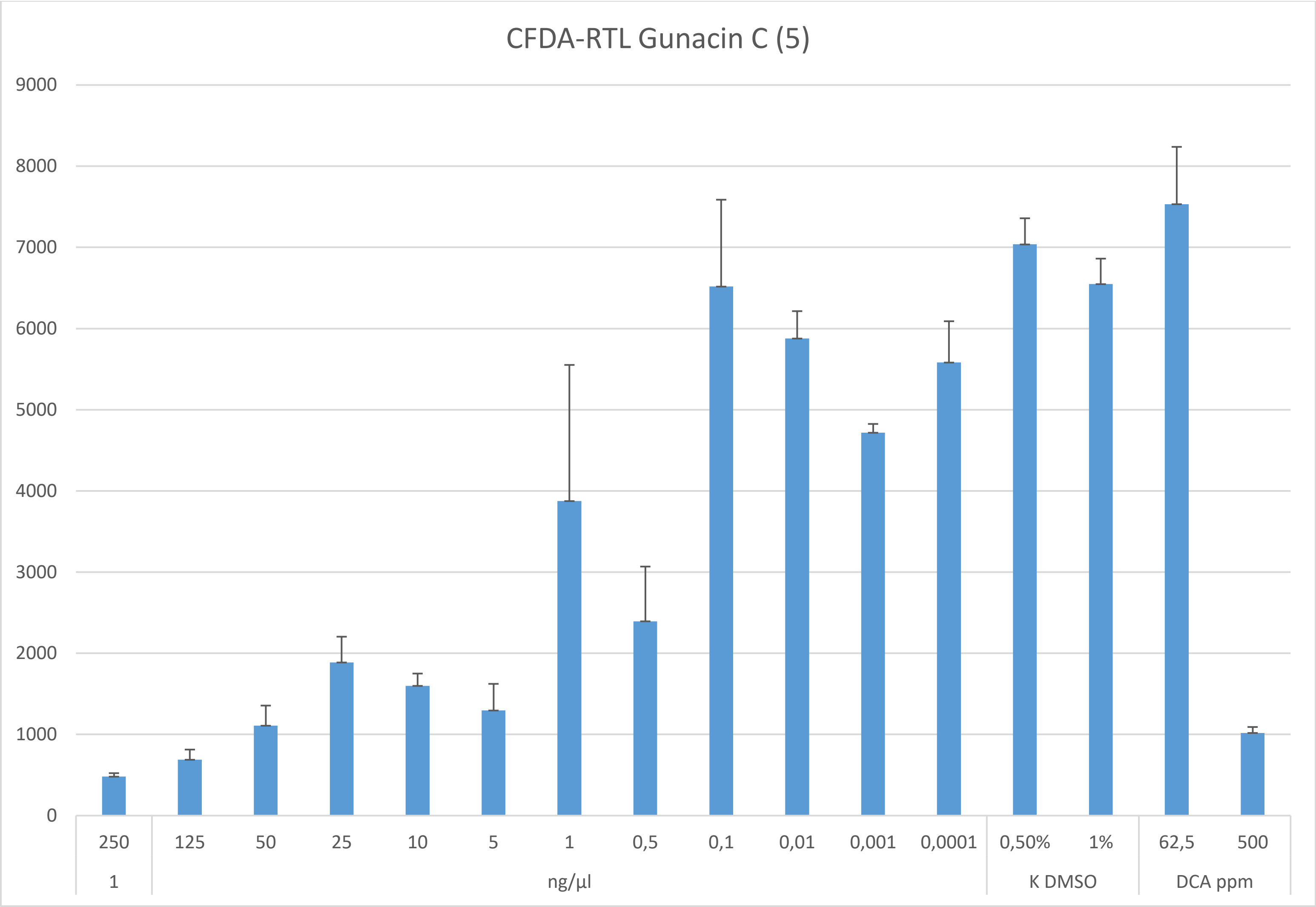

Figure S74 RTL-W1 cell line viability after treatment with **5** and 5-Carboxyfluorescein Diacetate Acetoxymethyl Ester (CFDA-AM) fluorescent indicator

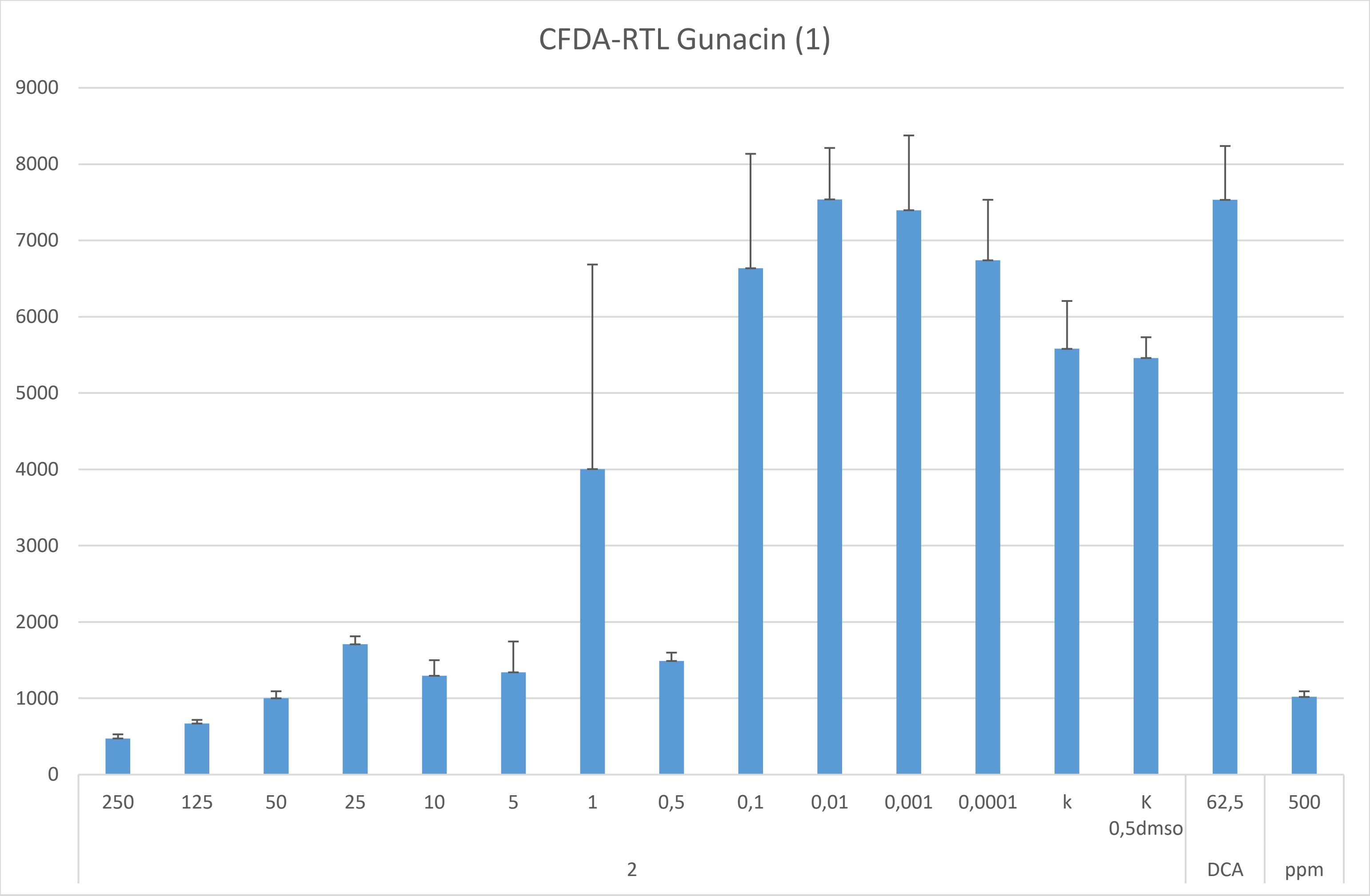

Figure S75 RTL-W1 cell line viability after treatment with **1** and 5-Carboxyfluorescein Diacetate Acetoxymethyl Ester (CFDA-AM) fluorescent indicator

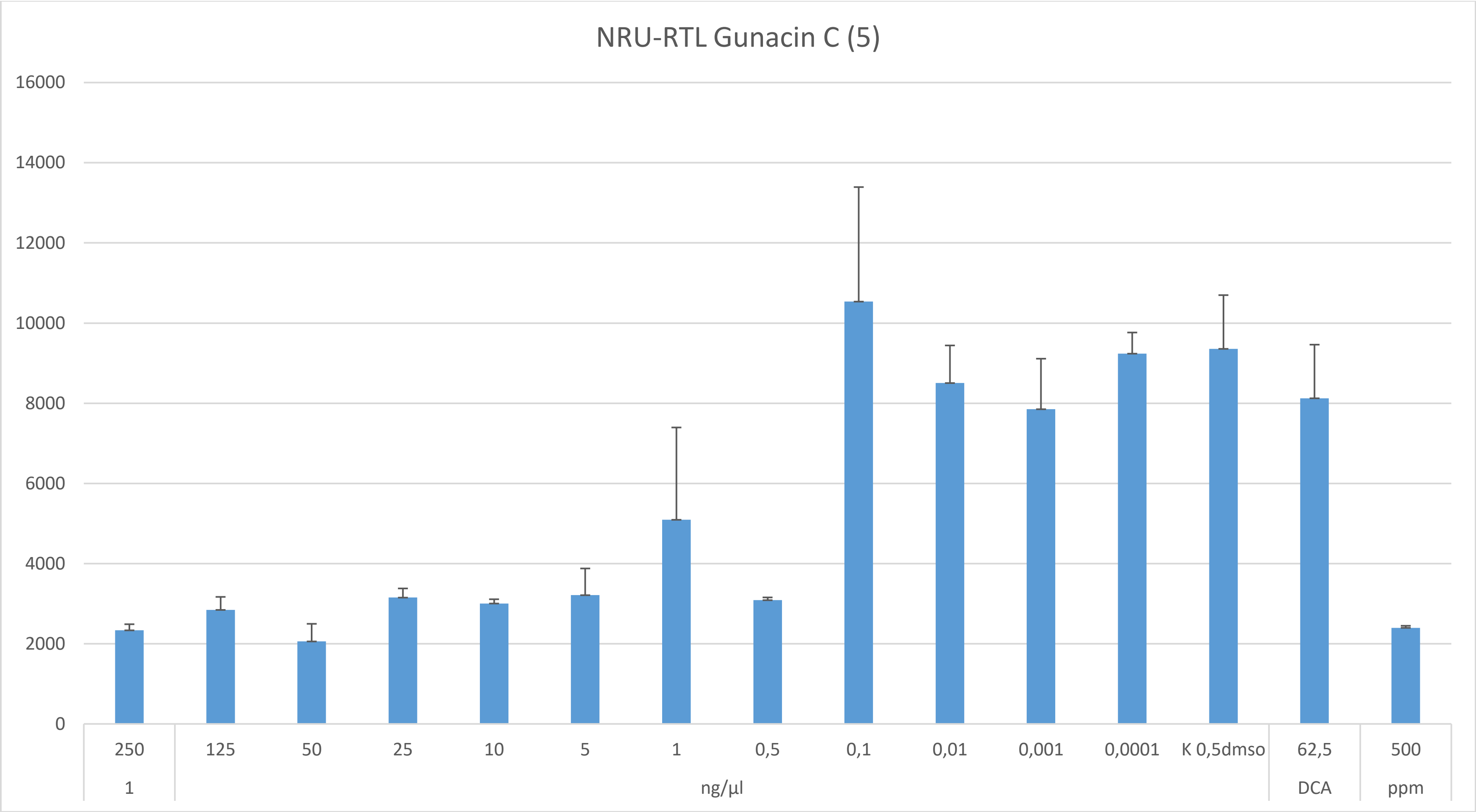

Figure S76 RTL-W1 cell line viability after treatment with **5** and neutral red (NR) fluorescent indicator

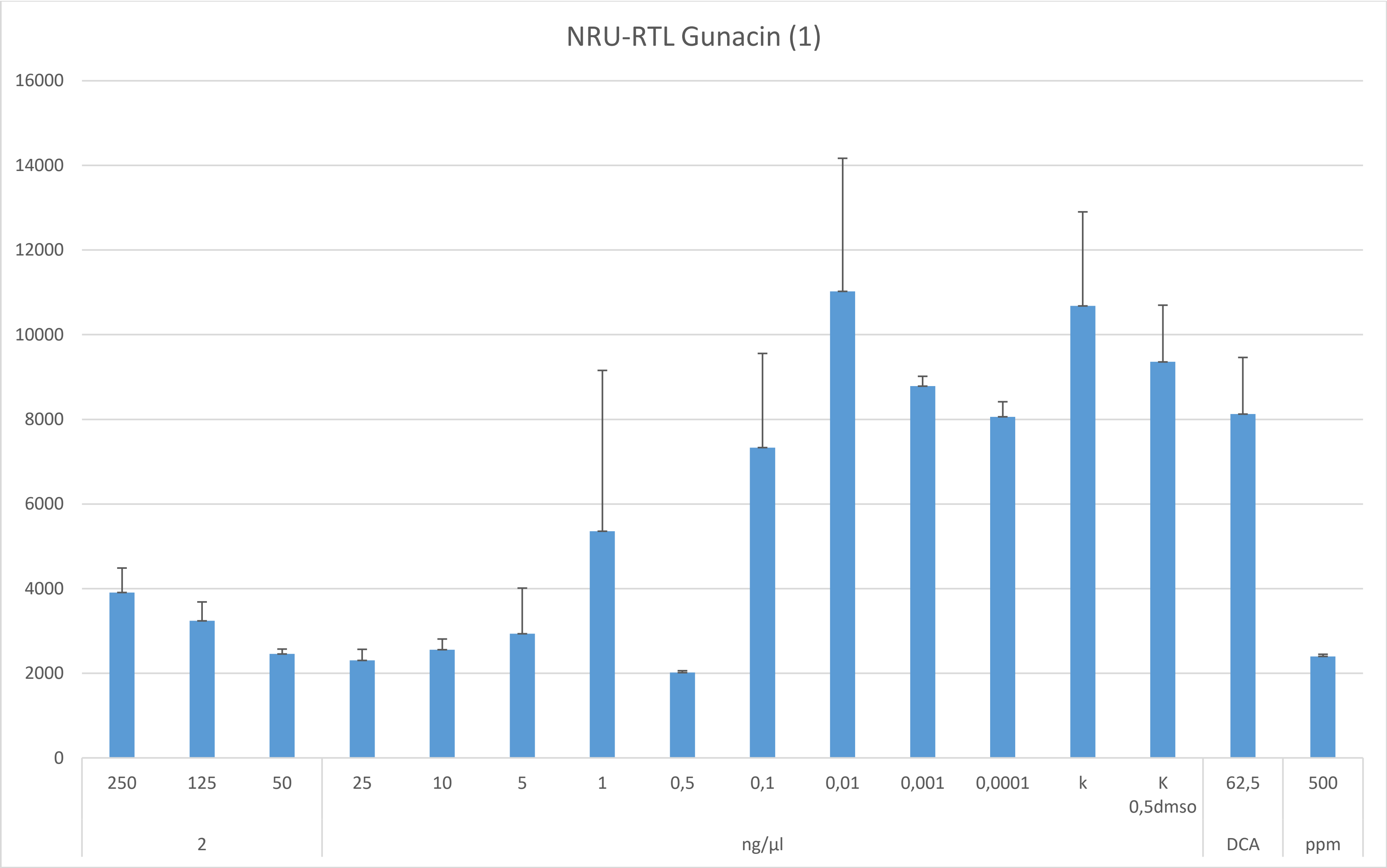

Figure S77 RTL-W1 cell line viability after treatment with **1** and neutral red (NR) fluorescent indicator
